# Supplementary material for: Short-term vital parameter forecasting in the intensive care unit: A benchmark study leveraging data from patients after cardiothoracic surgery
Source: PLOS Digit Health. 2024 Sep 12;3(9):e0000598. doi: 10.1371/journal.pdig.0000598 (PMC11392423; doi:10.1371/journal.pdig.0000598)
Supplement: S1 Table — For the internal dataset, missingness is reported pre and post resampling from 1- to 5-minute frequency. (DOCX) [file pdig.0000598.s002.docx]

**S1 Table:** Fraction of missing values per vital parameter. For the internal dataset, missingness is reported pre and post resampling from 1- to 5-minute frequency.

|  | **Internal data  (pre resampling)** | **Internal data  (post resampling)** | **External test data (eICU)** |
| --- | --- | --- | --- |
| BP Systolic (mmHg) | 0.061 | 0.027 | 0.213 |
| BP Diastolic (mmHg) | 0.063 | 0.027 | 0.214 |
| BP Mean (mmHg) | 0.057 | 0.027 | 0.213 |
| Central venous pressure (mmHg) | 0.029 | 0.025 | 0.522 |
| Oxygen saturation (%) | 0.033 | 0.018 | 0.058 |
| Heart rate (1/min) | 0.033 | 0.031 | 0.002 |
