# Supplementary material for: Short-term vital parameter forecasting in the intensive care unit: A benchmark study leveraging data from patients after cardiothoracic surgery
Source: PLOS Digit Health. 2024 Sep 12;3(9):e0000598. doi: 10.1371/journal.pdig.0000598 (PMC11392423; doi:10.1371/journal.pdig.0000598)
Supplement: S2 Table — BP = Blood pressure. SpO2 = Peripheral oxygen saturation. ETS = Exponential smoothing. ARIMA = Autoregressive integrated moving average. AR NNet = Autoregressive neural network. GRU = Gated recurrent unit. (DOCX) [file pdig.0000598.s003.docx]

**S2 Table:** Root mean squared error (RMSE), mean absolute error (MAE), and mean absolute percentage error (MAPE) per vital parameter, forecast model, forecast horizon and dataset (internal and external test sets). BP = Blood pressure. SpO2 = Peripheral oxygen saturation. ETS = Exponential smoothing. ARIMA = Autoregressive integrated moving average. AR NNet = Autoregressive neural network. GRU = Gated recurrent unit.

| **Dataset** | **Vital parameter** | **Model** | **Minutes ahead** | **RMSE** | **MAE** | **MAPE (%)** |
| --- | --- | --- | --- | --- | --- | --- |
| External test set (eICU) | Central venous pressure | AR NNet | 5 | 2.5499 | 1.4376 | 0.9104 |
| External test set (eICU) | Central venous pressure | AR NNet | 10 | 2.9107 | 1.7212 | 1.0898 |
| External test set (eICU) | Central venous pressure | AR NNet | 15 | 3.1372 | 1.9111 | 1.2102 |
| External test set (eICU) | Central venous pressure | AR NNet | 20 | 3.2995 | 2.0565 | 1.3022 |
| External test set (eICU) | Central venous pressure | AR NNet | 25 | 3.4382 | 2.1776 | 1.3790 |
| External test set (eICU) | Central venous pressure | AR NNet | 30 | 3.5543 | 2.2825 | 1.4455 |
| External test set (eICU) | Central venous pressure | AR NNet | 35 | 3.6499 | 2.3751 | 1.5042 |
| External test set (eICU) | Central venous pressure | AR NNet | 40 | 3.7318 | 2.4543 | 1.5545 |
| External test set (eICU) | Central venous pressure | AR NNet | 45 | 3.7975 | 2.5240 | 1.5987 |
| External test set (eICU) | Central venous pressure | AR NNet | 50 | 3.8617 | 2.5880 | 1.6393 |
| External test set (eICU) | Central venous pressure | AR NNet | 55 | 3.9248 | 2.6472 | 1.6769 |
| External test set (eICU) | Central venous pressure | AR NNet | 60 | 3.9798 | 2.7011 | 1.7111 |
| External test set (eICU) | Central venous pressure | AR NNet | 65 | 4.0344 | 2.7517 | 1.7432 |
| External test set (eICU) | Central venous pressure | AR NNet | 70 | 4.0833 | 2.7989 | 1.7731 |
| External test set (eICU) | Central venous pressure | AR NNet | 75 | 4.1294 | 2.8428 | 1.8010 |
| External test set (eICU) | Central venous pressure | AR NNet | 80 | 4.1674 | 2.8832 | 1.8266 |
| External test set (eICU) | Central venous pressure | AR NNet | 85 | 4.2051 | 2.9214 | 1.8509 |
| External test set (eICU) | Central venous pressure | AR NNet | 90 | 4.2400 | 2.9575 | 1.8738 |
| External test set (eICU) | Central venous pressure | AR NNet | 95 | 4.2776 | 2.9918 | 1.8956 |
| External test set (eICU) | Central venous pressure | AR NNet | 100 | 4.3095 | 3.0237 | 1.9158 |
| External test set (eICU) | Central venous pressure | AR NNet | 105 | 4.3482 | 3.0555 | 1.9360 |
| External test set (eICU) | Central venous pressure | AR NNet | 110 | 4.3790 | 3.0850 | 1.9546 |
| External test set (eICU) | Central venous pressure | AR NNet | 115 | 4.4108 | 3.1132 | 1.9725 |
| External test set (eICU) | Central venous pressure | AR NNet | 120 | 4.4366 | 3.1397 | 1.9892 |
| External test set (eICU) | Central venous pressure | ARIMA | 5 | 2.4903 | 1.2210 | 0.7723 |
| External test set (eICU) | Central venous pressure | ARIMA | 10 | 2.9214 | 1.5982 | 1.0110 |
| External test set (eICU) | Central venous pressure | ARIMA | 15 | 3.1755 | 1.8310 | 1.1586 |
| External test set (eICU) | Central venous pressure | ARIMA | 20 | 3.3540 | 2.0018 | 1.2669 |
| External test set (eICU) | Central venous pressure | ARIMA | 25 | 3.5249 | 2.1467 | 1.3587 |
| External test set (eICU) | Central venous pressure | ARIMA | 30 | 3.6507 | 2.2667 | 1.4348 |
| External test set (eICU) | Central venous pressure | ARIMA | 35 | 3.7734 | 2.3730 | 1.5023 |
| External test set (eICU) | Central venous pressure | ARIMA | 40 | 3.8688 | 2.4648 | 1.5606 |
| External test set (eICU) | Central venous pressure | ARIMA | 45 | 3.9536 | 2.5489 | 1.6141 |
| External test set (eICU) | Central venous pressure | ARIMA | 50 | 4.0382 | 2.6259 | 1.6630 |
| External test set (eICU) | Central venous pressure | ARIMA | 55 | 4.1063 | 2.6965 | 1.7078 |
| External test set (eICU) | Central venous pressure | ARIMA | 60 | 4.1750 | 2.7620 | 1.7494 |
| External test set (eICU) | Central venous pressure | ARIMA | 65 | 4.2437 | 2.8247 | 1.7891 |
| External test set (eICU) | Central venous pressure | ARIMA | 70 | 4.3095 | 2.8831 | 1.8260 |
| External test set (eICU) | Central venous pressure | ARIMA | 75 | 4.3700 | 2.9384 | 1.8612 |
| External test set (eICU) | Central venous pressure | ARIMA | 80 | 4.4241 | 2.9893 | 1.8935 |
| External test set (eICU) | Central venous pressure | ARIMA | 85 | 4.4816 | 3.0387 | 1.9249 |
| External test set (eICU) | Central venous pressure | ARIMA | 90 | 4.5339 | 3.0842 | 1.9538 |
| External test set (eICU) | Central venous pressure | ARIMA | 95 | 4.5806 | 3.1267 | 1.9808 |
| External test set (eICU) | Central venous pressure | ARIMA | 100 | 4.6248 | 3.1674 | 2.0067 |
| External test set (eICU) | Central venous pressure | ARIMA | 105 | 4.6723 | 3.2069 | 2.0317 |
| External test set (eICU) | Central venous pressure | ARIMA | 110 | 4.7126 | 3.2433 | 2.0548 |
| External test set (eICU) | Central venous pressure | ARIMA | 115 | 4.7538 | 3.2784 | 2.0771 |
| External test set (eICU) | Central venous pressure | ARIMA | 120 | 4.7936 | 3.3127 | 2.0988 |
| External test set (eICU) | Central venous pressure | ETS | 5 | 2.6082 | 1.1627 | 0.7365 |
| External test set (eICU) | Central venous pressure | ETS | 10 | 3.0653 | 1.5312 | 0.9699 |
| External test set (eICU) | Central venous pressure | ETS | 15 | 3.3598 | 1.7748 | 1.1245 |
| External test set (eICU) | Central venous pressure | ETS | 20 | 3.5811 | 1.9636 | 1.2442 |
| External test set (eICU) | Central venous pressure | ETS | 25 | 3.7582 | 2.1196 | 1.3431 |
| External test set (eICU) | Central venous pressure | ETS | 30 | 3.9098 | 2.2546 | 1.4285 |
| External test set (eICU) | Central venous pressure | ETS | 35 | 4.0426 | 2.3723 | 1.5032 |
| External test set (eICU) | Central venous pressure | ETS | 40 | 4.1617 | 2.4776 | 1.5699 |
| External test set (eICU) | Central venous pressure | ETS | 45 | 4.2711 | 2.5751 | 1.6317 |
| External test set (eICU) | Central venous pressure | ETS | 50 | 4.3692 | 2.6624 | 1.6871 |
| External test set (eICU) | Central venous pressure | ETS | 55 | 4.4599 | 2.7453 | 1.7396 |
| External test set (eICU) | Central venous pressure | ETS | 60 | 4.5436 | 2.8204 | 1.7872 |
| External test set (eICU) | Central venous pressure | ETS | 65 | 4.6218 | 2.8922 | 1.8326 |
| External test set (eICU) | Central venous pressure | ETS | 70 | 4.6967 | 2.9594 | 1.8751 |
| External test set (eICU) | Central venous pressure | ETS | 75 | 4.7682 | 3.0228 | 1.9153 |
| External test set (eICU) | Central venous pressure | ETS | 80 | 4.8344 | 3.0821 | 1.9530 |
| External test set (eICU) | Central venous pressure | ETS | 85 | 4.8984 | 3.1380 | 1.9885 |
| External test set (eICU) | Central venous pressure | ETS | 90 | 4.9565 | 3.1902 | 2.0217 |
| External test set (eICU) | Central venous pressure | ETS | 95 | 5.0085 | 3.2389 | 2.0525 |
| External test set (eICU) | Central venous pressure | ETS | 100 | 5.0609 | 3.2850 | 2.0818 |
| External test set (eICU) | Central venous pressure | ETS | 105 | 5.1093 | 3.3283 | 2.1091 |
| External test set (eICU) | Central venous pressure | ETS | 110 | 5.1558 | 3.3692 | 2.1350 |
| External test set (eICU) | Central venous pressure | ETS | 115 | 5.2026 | 3.4080 | 2.1595 |
| External test set (eICU) | Central venous pressure | ETS | 120 | 5.2448 | 3.4443 | 2.1824 |
| External test set (eICU) | Central venous pressure | GRU | 5 | 2.0887 | 1.1110 | 0.7027 |
| External test set (eICU) | Central venous pressure | GRU | 10 | 2.5330 | 1.4613 | 0.9249 |
| External test set (eICU) | Central venous pressure | GRU | 15 | 2.7948 | 1.6845 | 1.0664 |
| External test set (eICU) | Central venous pressure | GRU | 20 | 2.9856 | 1.8510 | 1.1719 |
| External test set (eICU) | Central venous pressure | GRU | 25 | 3.1380 | 1.9886 | 1.2590 |
| External test set (eICU) | Central venous pressure | GRU | 30 | 3.2650 | 2.1043 | 1.3322 |
| External test set (eICU) | Central venous pressure | GRU | 35 | 3.3714 | 2.2036 | 1.3949 |
| External test set (eICU) | Central venous pressure | GRU | 40 | 3.4634 | 2.2874 | 1.4481 |
| External test set (eICU) | Central venous pressure | GRU | 45 | 3.5454 | 2.3646 | 1.4969 |
| External test set (eICU) | Central venous pressure | GRU | 50 | 3.6195 | 2.4342 | 1.5409 |
| External test set (eICU) | Central venous pressure | GRU | 55 | 3.6845 | 2.4951 | 1.5794 |
| External test set (eICU) | Central venous pressure | GRU | 60 | 3.7467 | 2.5524 | 1.6156 |
| External test set (eICU) | Central venous pressure | GRU | 65 | 3.8037 | 2.6072 | 1.6503 |
| External test set (eICU) | Central venous pressure | GRU | 70 | 3.8537 | 2.6546 | 1.6804 |
| External test set (eICU) | Central venous pressure | GRU | 75 | 3.9045 | 2.7010 | 1.7096 |
| External test set (eICU) | Central venous pressure | GRU | 80 | 3.9474 | 2.7428 | 1.7363 |
| External test set (eICU) | Central venous pressure | GRU | 85 | 3.9917 | 2.7837 | 1.7621 |
| External test set (eICU) | Central venous pressure | GRU | 90 | 4.0313 | 2.8211 | 1.7857 |
| External test set (eICU) | Central venous pressure | GRU | 95 | 4.0705 | 2.8575 | 1.8087 |
| External test set (eICU) | Central venous pressure | GRU | 100 | 4.1051 | 2.8914 | 1.8300 |
| External test set (eICU) | Central venous pressure | GRU | 105 | 4.1392 | 2.9233 | 1.8501 |
| External test set (eICU) | Central venous pressure | GRU | 110 | 4.1679 | 2.9513 | 1.8679 |
| External test set (eICU) | Central venous pressure | GRU | 115 | 4.1993 | 2.9801 | 1.8860 |
| External test set (eICU) | Central venous pressure | GRU | 120 | 4.2280 | 3.0071 | 1.9031 |
| External test set (eICU) | Central venous pressure | Naive | 5 | 2.2021 | 1.0333 | 0.6532 |
| External test set (eICU) | Central venous pressure | Naive | 10 | 2.7620 | 1.4409 | 0.9113 |
| External test set (eICU) | Central venous pressure | Naive | 15 | 3.0889 | 1.6969 | 1.0737 |
| External test set (eICU) | Central venous pressure | Naive | 20 | 3.3195 | 1.8901 | 1.1962 |
| External test set (eICU) | Central venous pressure | Naive | 25 | 3.4987 | 2.0476 | 1.2961 |
| External test set (eICU) | Central venous pressure | Naive | 30 | 3.6473 | 2.1819 | 1.3810 |
| External test set (eICU) | Central venous pressure | Naive | 35 | 3.7751 | 2.2988 | 1.4551 |
| External test set (eICU) | Central venous pressure | Naive | 40 | 3.8879 | 2.4015 | 1.5202 |
| External test set (eICU) | Central venous pressure | Naive | 45 | 3.9893 | 2.4961 | 1.5801 |
| External test set (eICU) | Central venous pressure | Naive | 50 | 4.0790 | 2.5801 | 1.6334 |
| External test set (eICU) | Central venous pressure | Naive | 55 | 4.1617 | 2.6604 | 1.6842 |
| External test set (eICU) | Central venous pressure | Naive | 60 | 4.2346 | 2.7313 | 1.7291 |
| External test set (eICU) | Central venous pressure | Naive | 65 | 4.3035 | 2.7998 | 1.7725 |
| External test set (eICU) | Central venous pressure | Naive | 70 | 4.3682 | 2.8632 | 1.8125 |
| External test set (eICU) | Central venous pressure | Naive | 75 | 4.4275 | 2.9228 | 1.8503 |
| External test set (eICU) | Central venous pressure | Naive | 80 | 4.4836 | 2.9783 | 1.8856 |
| External test set (eICU) | Central venous pressure | Naive | 85 | 4.5384 | 3.0304 | 1.9187 |
| External test set (eICU) | Central venous pressure | Naive | 90 | 4.5855 | 3.0786 | 1.9494 |
| External test set (eICU) | Central venous pressure | Naive | 95 | 4.6288 | 3.1239 | 1.9780 |
| External test set (eICU) | Central venous pressure | Naive | 100 | 4.6690 | 3.1662 | 2.0048 |
| External test set (eICU) | Central venous pressure | Naive | 105 | 4.7093 | 3.2065 | 2.0303 |
| External test set (eICU) | Central venous pressure | Naive | 110 | 4.7462 | 3.2441 | 2.0540 |
| External test set (eICU) | Central venous pressure | Naive | 115 | 4.7824 | 3.2797 | 2.0765 |
| External test set (eICU) | Central venous pressure | Naive | 120 | 4.8153 | 3.3118 | 2.0967 |
| External test set (eICU) | Central venous pressure | Theta | 5 | 3.6141 | 1.3205 | 0.8377 |
| External test set (eICU) | Central venous pressure | Theta | 10 | 3.9734 | 1.6977 | 1.0768 |
| External test set (eICU) | Central venous pressure | Theta | 15 | 4.2110 | 1.9480 | 1.2357 |
| External test set (eICU) | Central venous pressure | Theta | 20 | 4.3953 | 2.1443 | 1.3602 |
| External test set (eICU) | Central venous pressure | Theta | 25 | 4.5436 | 2.3076 | 1.4638 |
| External test set (eICU) | Central venous pressure | Theta | 30 | 4.6767 | 2.4515 | 1.5548 |
| External test set (eICU) | Central venous pressure | Theta | 35 | 4.7926 | 2.5780 | 1.6350 |
| External test set (eICU) | Central venous pressure | Theta | 40 | 4.9031 | 2.6940 | 1.7085 |
| External test set (eICU) | Central venous pressure | Theta | 45 | 5.0039 | 2.8014 | 1.7766 |
| External test set (eICU) | Central venous pressure | Theta | 50 | 5.1009 | 2.9013 | 1.8400 |
| External test set (eICU) | Central venous pressure | Theta | 55 | 5.1879 | 2.9952 | 1.8996 |
| External test set (eICU) | Central venous pressure | Theta | 60 | 5.2740 | 3.0828 | 1.9551 |
| External test set (eICU) | Central venous pressure | Theta | 65 | 5.3533 | 3.1669 | 2.0084 |
| External test set (eICU) | Central venous pressure | Theta | 70 | 5.4338 | 3.2478 | 2.0596 |
| External test set (eICU) | Central venous pressure | Theta | 75 | 5.5087 | 3.3246 | 2.1084 |
| External test set (eICU) | Central venous pressure | Theta | 80 | 5.5838 | 3.3988 | 2.1556 |
| External test set (eICU) | Central venous pressure | Theta | 85 | 5.6562 | 3.4692 | 2.2003 |
| External test set (eICU) | Central venous pressure | Theta | 90 | 5.7255 | 3.5372 | 2.2436 |
| External test set (eICU) | Central venous pressure | Theta | 95 | 5.7907 | 3.6021 | 2.2848 |
| External test set (eICU) | Central venous pressure | Theta | 100 | 5.8559 | 3.6644 | 2.3243 |
| External test set (eICU) | Central venous pressure | Theta | 105 | 5.9188 | 3.7246 | 2.3624 |
| External test set (eICU) | Central venous pressure | Theta | 110 | 5.9824 | 3.7830 | 2.3995 |
| External test set (eICU) | Central venous pressure | Theta | 115 | 6.0423 | 3.8388 | 2.4348 |
| External test set (eICU) | Central venous pressure | Theta | 120 | 6.1056 | 3.8937 | 2.4696 |
| External test set (eICU) | Central venous pressure | Transformer | 5 | 2.1422 | 1.2009 | 0.7594 |
| External test set (eICU) | Central venous pressure | Transformer | 10 | 2.5887 | 1.5288 | 0.9669 |
| External test set (eICU) | Central venous pressure | Transformer | 15 | 2.8473 | 1.7367 | 1.0986 |
| External test set (eICU) | Central venous pressure | Transformer | 20 | 3.0323 | 1.8935 | 1.1980 |
| External test set (eICU) | Central venous pressure | Transformer | 25 | 3.1791 | 2.0222 | 1.2794 |
| External test set (eICU) | Central venous pressure | Transformer | 30 | 3.3028 | 2.1327 | 1.3493 |
| External test set (eICU) | Central venous pressure | Transformer | 35 | 3.4039 | 2.2258 | 1.4082 |
| External test set (eICU) | Central venous pressure | Transformer | 40 | 3.4918 | 2.3067 | 1.4594 |
| External test set (eICU) | Central venous pressure | Transformer | 45 | 3.5713 | 2.3799 | 1.5059 |
| External test set (eICU) | Central venous pressure | Transformer | 50 | 3.6419 | 2.4455 | 1.5475 |
| External test set (eICU) | Central venous pressure | Transformer | 55 | 3.7059 | 2.5054 | 1.5854 |
| External test set (eICU) | Central venous pressure | Transformer | 60 | 3.7656 | 2.5617 | 1.6211 |
| External test set (eICU) | Central venous pressure | Transformer | 65 | 3.8198 | 2.6132 | 1.6537 |
| External test set (eICU) | Central venous pressure | Transformer | 70 | 3.8699 | 2.6607 | 1.6838 |
| External test set (eICU) | Central venous pressure | Transformer | 75 | 3.9171 | 2.7057 | 1.7123 |
| External test set (eICU) | Central venous pressure | Transformer | 80 | 3.9615 | 2.7479 | 1.7390 |
| External test set (eICU) | Central venous pressure | Transformer | 85 | 4.0031 | 2.7872 | 1.7640 |
| External test set (eICU) | Central venous pressure | Transformer | 90 | 4.0416 | 2.8236 | 1.7871 |
| External test set (eICU) | Central venous pressure | Transformer | 95 | 4.0770 | 2.8581 | 1.8088 |
| External test set (eICU) | Central venous pressure | Transformer | 100 | 4.1111 | 2.8911 | 1.8297 |
| External test set (eICU) | Central venous pressure | Transformer | 105 | 4.1431 | 2.9217 | 1.8491 |
| External test set (eICU) | Central venous pressure | Transformer | 110 | 4.1736 | 2.9506 | 1.8673 |
| External test set (eICU) | Central venous pressure | Transformer | 115 | 4.2027 | 2.9782 | 1.8848 |
| External test set (eICU) | Central venous pressure | Transformer | 120 | 4.2303 | 3.0043 | 1.9012 |
| External test set (eICU) | Diastolic BP | AR NNet | 5 | 5.2629 | 3.0284 | 1.8677 |
| External test set (eICU) | Diastolic BP | AR NNet | 10 | 5.8430 | 3.5249 | 2.1724 |
| External test set (eICU) | Diastolic BP | AR NNet | 15 | 6.1776 | 3.8296 | 2.3597 |
| External test set (eICU) | Diastolic BP | AR NNet | 20 | 6.4375 | 4.0667 | 2.5061 |
| External test set (eICU) | Diastolic BP | AR NNet | 25 | 6.6414 | 4.2571 | 2.6236 |
| External test set (eICU) | Diastolic BP | AR NNet | 30 | 6.8105 | 4.4169 | 2.7221 |
| External test set (eICU) | Diastolic BP | AR NNet | 35 | 6.9823 | 4.5705 | 2.8169 |
| External test set (eICU) | Diastolic BP | AR NNet | 40 | 7.1058 | 4.6865 | 2.8885 |
| External test set (eICU) | Diastolic BP | AR NNet | 45 | 7.2197 | 4.7895 | 2.9521 |
| External test set (eICU) | Diastolic BP | AR NNet | 50 | 7.3281 | 4.8869 | 3.0123 |
| External test set (eICU) | Diastolic BP | AR NNet | 55 | 7.4216 | 4.9720 | 3.0649 |
| External test set (eICU) | Diastolic BP | AR NNet | 60 | 7.5097 | 5.0505 | 3.1136 |
| External test set (eICU) | Diastolic BP | AR NNet | 65 | 7.6103 | 5.1333 | 3.1649 |
| External test set (eICU) | Diastolic BP | AR NNet | 70 | 7.6765 | 5.1954 | 3.2034 |
| External test set (eICU) | Diastolic BP | AR NNet | 75 | 7.7422 | 5.2542 | 3.2398 |
| External test set (eICU) | Diastolic BP | AR NNet | 80 | 7.8074 | 5.3123 | 3.2758 |
| External test set (eICU) | Diastolic BP | AR NNet | 85 | 7.8666 | 5.3648 | 3.3083 |
| External test set (eICU) | Diastolic BP | AR NNet | 90 | 7.9216 | 5.4139 | 3.3387 |
| External test set (eICU) | Diastolic BP | AR NNet | 95 | 7.9832 | 5.4652 | 3.3705 |
| External test set (eICU) | Diastolic BP | AR NNet | 100 | 8.0312 | 5.5081 | 3.3970 |
| External test set (eICU) | Diastolic BP | AR NNet | 105 | 8.0784 | 5.5493 | 3.4225 |
| External test set (eICU) | Diastolic BP | AR NNet | 110 | 8.1307 | 5.5938 | 3.4500 |
| External test set (eICU) | Diastolic BP | AR NNet | 115 | 8.1785 | 5.6349 | 3.4754 |
| External test set (eICU) | Diastolic BP | AR NNet | 120 | 8.2264 | 5.6759 | 3.5008 |
| External test set (eICU) | Diastolic BP | ARIMA | 5 | 5.2141 | 2.7272 | 1.6804 |
| External test set (eICU) | Diastolic BP | ARIMA | 10 | 5.9587 | 3.4283 | 2.1117 |
| External test set (eICU) | Diastolic BP | ARIMA | 15 | 6.3530 | 3.8077 | 2.3456 |
| External test set (eICU) | Diastolic BP | ARIMA | 20 | 6.6593 | 4.0926 | 2.5214 |
| External test set (eICU) | Diastolic BP | ARIMA | 25 | 6.9069 | 4.3234 | 2.6639 |
| External test set (eICU) | Diastolic BP | ARIMA | 30 | 7.1169 | 4.5135 | 2.7814 |
| External test set (eICU) | Diastolic BP | ARIMA | 35 | 7.3269 | 4.6936 | 2.8928 |
| External test set (eICU) | Diastolic BP | ARIMA | 40 | 7.4903 | 4.8383 | 2.9821 |
| External test set (eICU) | Diastolic BP | ARIMA | 45 | 7.6363 | 4.9613 | 3.0580 |
| External test set (eICU) | Diastolic BP | ARIMA | 50 | 7.7837 | 5.0850 | 3.1347 |
| External test set (eICU) | Diastolic BP | ARIMA | 55 | 7.9116 | 5.1915 | 3.2007 |
| External test set (eICU) | Diastolic BP | ARIMA | 60 | 8.0339 | 5.2913 | 3.2627 |
| External test set (eICU) | Diastolic BP | ARIMA | 65 | 8.1694 | 5.3982 | 3.3289 |
| External test set (eICU) | Diastolic BP | ARIMA | 70 | 8.2728 | 5.4824 | 3.3810 |
| External test set (eICU) | Diastolic BP | ARIMA | 75 | 8.3678 | 5.5579 | 3.4277 |
| External test set (eICU) | Diastolic BP | ARIMA | 80 | 8.4609 | 5.6322 | 3.4740 |
| External test set (eICU) | Diastolic BP | ARIMA | 85 | 8.5453 | 5.6985 | 3.5149 |
| External test set (eICU) | Diastolic BP | ARIMA | 90 | 8.6312 | 5.7626 | 3.5546 |
| External test set (eICU) | Diastolic BP | ARIMA | 95 | 8.7255 | 5.8308 | 3.5969 |
| External test set (eICU) | Diastolic BP | ARIMA | 100 | 8.8011 | 5.8878 | 3.6323 |
| External test set (eICU) | Diastolic BP | ARIMA | 105 | 8.8714 | 5.9372 | 3.6629 |
| External test set (eICU) | Diastolic BP | ARIMA | 110 | 8.9516 | 5.9950 | 3.6986 |
| External test set (eICU) | Diastolic BP | ARIMA | 115 | 9.0235 | 6.0474 | 3.7309 |
| External test set (eICU) | Diastolic BP | ARIMA | 120 | 9.1003 | 6.1007 | 3.7639 |
| External test set (eICU) | Diastolic BP | ETS | 5 | 5.3421 | 2.6889 | 1.6573 |
| External test set (eICU) | Diastolic BP | ETS | 10 | 6.1555 | 3.3746 | 2.0790 |
| External test set (eICU) | Diastolic BP | ETS | 15 | 6.6147 | 3.7820 | 2.3300 |
| External test set (eICU) | Diastolic BP | ETS | 20 | 6.9978 | 4.1132 | 2.5341 |
| External test set (eICU) | Diastolic BP | ETS | 25 | 7.2962 | 4.3787 | 2.6978 |
| External test set (eICU) | Diastolic BP | ETS | 30 | 7.5341 | 4.5972 | 2.8324 |
| External test set (eICU) | Diastolic BP | ETS | 35 | 7.8021 | 4.8202 | 2.9700 |
| External test set (eICU) | Diastolic BP | ETS | 40 | 7.9673 | 4.9773 | 3.0668 |
| External test set (eICU) | Diastolic BP | ETS | 45 | 8.0839 | 5.0889 | 3.1353 |
| External test set (eICU) | Diastolic BP | ETS | 50 | 8.2546 | 5.2341 | 3.2248 |
| External test set (eICU) | Diastolic BP | ETS | 55 | 8.3971 | 5.3562 | 3.3003 |
| External test set (eICU) | Diastolic BP | ETS | 60 | 8.5209 | 5.4641 | 3.3668 |
| External test set (eICU) | Diastolic BP | ETS | 65 | 8.7280 | 5.6199 | 3.4631 |
| External test set (eICU) | Diastolic BP | ETS | 70 | 8.8285 | 5.7094 | 3.5182 |
| External test set (eICU) | Diastolic BP | ETS | 75 | 8.9249 | 5.7876 | 3.5667 |
| External test set (eICU) | Diastolic BP | ETS | 80 | 9.0200 | 5.8712 | 3.6185 |
| External test set (eICU) | Diastolic BP | ETS | 85 | 9.1094 | 5.9444 | 3.6638 |
| External test set (eICU) | Diastolic BP | ETS | 90 | 9.1921 | 6.0090 | 3.7037 |
| External test set (eICU) | Diastolic BP | ETS | 95 | 9.3002 | 6.0896 | 3.7535 |
| External test set (eICU) | Diastolic BP | ETS | 100 | 9.3651 | 6.1418 | 3.7858 |
| External test set (eICU) | Diastolic BP | ETS | 105 | 9.4016 | 6.1691 | 3.8025 |
| External test set (eICU) | Diastolic BP | ETS | 110 | 9.4744 | 6.2276 | 3.8385 |
| External test set (eICU) | Diastolic BP | ETS | 115 | 9.5497 | 6.2810 | 3.8715 |
| External test set (eICU) | Diastolic BP | ETS | 120 | 9.6124 | 6.3289 | 3.9009 |
| External test set (eICU) | Diastolic BP | GRU | 5 | 4.9716 | 2.6392 | 1.6279 |
| External test set (eICU) | Diastolic BP | GRU | 10 | 5.6174 | 3.2703 | 2.0168 |
| External test set (eICU) | Diastolic BP | GRU | 15 | 5.9510 | 3.6074 | 2.2255 |
| External test set (eICU) | Diastolic BP | GRU | 20 | 6.2374 | 3.8799 | 2.3944 |
| External test set (eICU) | Diastolic BP | GRU | 25 | 6.4530 | 4.0945 | 2.5273 |
| External test set (eICU) | Diastolic BP | GRU | 30 | 6.6412 | 4.2708 | 2.6366 |
| External test set (eICU) | Diastolic BP | GRU | 35 | 6.8141 | 4.4320 | 2.7368 |
| External test set (eICU) | Diastolic BP | GRU | 40 | 6.9250 | 4.5443 | 2.8063 |
| External test set (eICU) | Diastolic BP | GRU | 45 | 7.0080 | 4.6340 | 2.8615 |
| External test set (eICU) | Diastolic BP | GRU | 50 | 7.1277 | 4.7411 | 2.9281 |
| External test set (eICU) | Diastolic BP | GRU | 55 | 7.2265 | 4.8272 | 2.9811 |
| External test set (eICU) | Diastolic BP | GRU | 60 | 7.3192 | 4.9116 | 3.0335 |
| External test set (eICU) | Diastolic BP | GRU | 65 | 7.4296 | 5.0002 | 3.0886 |
| External test set (eICU) | Diastolic BP | GRU | 70 | 7.4974 | 5.0601 | 3.1254 |
| External test set (eICU) | Diastolic BP | GRU | 75 | 7.5515 | 5.1131 | 3.1582 |
| External test set (eICU) | Diastolic BP | GRU | 80 | 7.6193 | 5.1707 | 3.1936 |
| External test set (eICU) | Diastolic BP | GRU | 85 | 7.6762 | 5.2223 | 3.2254 |
| External test set (eICU) | Diastolic BP | GRU | 90 | 7.7320 | 5.2664 | 3.2525 |
| External test set (eICU) | Diastolic BP | GRU | 95 | 7.7925 | 5.3195 | 3.2853 |
| External test set (eICU) | Diastolic BP | GRU | 100 | 7.8342 | 5.3582 | 3.3091 |
| External test set (eICU) | Diastolic BP | GRU | 105 | 7.8657 | 5.3827 | 3.3237 |
| External test set (eICU) | Diastolic BP | GRU | 110 | 7.9117 | 5.4255 | 3.3504 |
| External test set (eICU) | Diastolic BP | GRU | 115 | 7.9518 | 5.4612 | 3.3724 |
| External test set (eICU) | Diastolic BP | GRU | 120 | 8.0020 | 5.5026 | 3.3977 |
| External test set (eICU) | Diastolic BP | Naive | 5 | 5.4698 | 2.6123 | 1.6101 |
| External test set (eICU) | Diastolic BP | Naive | 10 | 6.3847 | 3.4035 | 2.0961 |
| External test set (eICU) | Diastolic BP | Naive | 15 | 6.7964 | 3.8150 | 2.3494 |
| External test set (eICU) | Diastolic BP | Naive | 20 | 7.1515 | 4.1494 | 2.5552 |
| External test set (eICU) | Diastolic BP | Naive | 25 | 7.4290 | 4.4170 | 2.7202 |
| External test set (eICU) | Diastolic BP | Naive | 30 | 7.6601 | 4.6385 | 2.8566 |
| External test set (eICU) | Diastolic BP | Naive | 35 | 7.9248 | 4.8715 | 3.0001 |
| External test set (eICU) | Diastolic BP | Naive | 40 | 8.0877 | 5.0341 | 3.1004 |
| External test set (eICU) | Diastolic BP | Naive | 45 | 8.1328 | 5.1177 | 3.1516 |
| External test set (eICU) | Diastolic BP | Naive | 50 | 8.2773 | 5.2555 | 3.2367 |
| External test set (eICU) | Diastolic BP | Naive | 55 | 8.3989 | 5.3744 | 3.3099 |
| External test set (eICU) | Diastolic BP | Naive | 60 | 8.5058 | 5.4785 | 3.3743 |
| External test set (eICU) | Diastolic BP | Naive | 65 | 8.7029 | 5.6370 | 3.4719 |
| External test set (eICU) | Diastolic BP | Naive | 70 | 8.7859 | 5.7235 | 3.5253 |
| External test set (eICU) | Diastolic BP | Naive | 75 | 8.8322 | 5.7803 | 3.5605 |
| External test set (eICU) | Diastolic BP | Naive | 80 | 8.9004 | 5.8550 | 3.6069 |
| External test set (eICU) | Diastolic BP | Naive | 85 | 8.9621 | 5.9214 | 3.6478 |
| External test set (eICU) | Diastolic BP | Naive | 90 | 9.0292 | 5.9813 | 3.6849 |
| External test set (eICU) | Diastolic BP | Naive | 95 | 9.1263 | 6.0633 | 3.7352 |
| External test set (eICU) | Diastolic BP | Naive | 100 | 9.1840 | 6.1172 | 3.7685 |
| External test set (eICU) | Diastolic BP | Naive | 105 | 9.1590 | 6.1177 | 3.7688 |
| External test set (eICU) | Diastolic BP | Naive | 110 | 9.2090 | 6.1663 | 3.7988 |
| External test set (eICU) | Diastolic BP | Naive | 115 | 9.2580 | 6.2123 | 3.8270 |
| External test set (eICU) | Diastolic BP | Naive | 120 | 9.3122 | 6.2579 | 3.8550 |
| External test set (eICU) | Diastolic BP | Theta | 5 | 5.3398 | 2.6920 | 1.6592 |
| External test set (eICU) | Diastolic BP | Theta | 10 | 6.1541 | 3.3891 | 2.0881 |
| External test set (eICU) | Diastolic BP | Theta | 15 | 6.6133 | 3.8057 | 2.3449 |
| External test set (eICU) | Diastolic BP | Theta | 20 | 7.0116 | 4.1518 | 2.5583 |
| External test set (eICU) | Diastolic BP | Theta | 25 | 7.3161 | 4.4318 | 2.7311 |
| External test set (eICU) | Diastolic BP | Theta | 30 | 7.5675 | 4.6664 | 2.8758 |
| External test set (eICU) | Diastolic BP | Theta | 35 | 7.8588 | 4.9104 | 3.0264 |
| External test set (eICU) | Diastolic BP | Theta | 40 | 8.0464 | 5.0873 | 3.1355 |
| External test set (eICU) | Diastolic BP | Theta | 45 | 8.1875 | 5.2207 | 3.2175 |
| External test set (eICU) | Diastolic BP | Theta | 50 | 8.3962 | 5.3913 | 3.3229 |
| External test set (eICU) | Diastolic BP | Theta | 55 | 8.5607 | 5.5362 | 3.4124 |
| External test set (eICU) | Diastolic BP | Theta | 60 | 8.7102 | 5.6670 | 3.4932 |
| External test set (eICU) | Diastolic BP | Theta | 65 | 8.9631 | 5.8542 | 3.6089 |
| External test set (eICU) | Diastolic BP | Theta | 70 | 9.0980 | 5.9702 | 3.6806 |
| External test set (eICU) | Diastolic BP | Theta | 75 | 9.2313 | 6.0761 | 3.7462 |
| External test set (eICU) | Diastolic BP | Theta | 80 | 9.3792 | 6.1917 | 3.8180 |
| External test set (eICU) | Diastolic BP | Theta | 85 | 9.5042 | 6.2930 | 3.8807 |
| External test set (eICU) | Diastolic BP | Theta | 90 | 9.6243 | 6.3864 | 3.9384 |
| External test set (eICU) | Diastolic BP | Theta | 95 | 9.7840 | 6.5006 | 4.0090 |
| External test set (eICU) | Diastolic BP | Theta | 100 | 9.8944 | 6.5852 | 4.0615 |
| External test set (eICU) | Diastolic BP | Theta | 105 | 9.9842 | 6.6479 | 4.1002 |
| External test set (eICU) | Diastolic BP | Theta | 110 | 10.1182 | 6.7421 | 4.1588 |
| External test set (eICU) | Diastolic BP | Theta | 115 | 10.2329 | 6.8252 | 4.2101 |
| External test set (eICU) | Diastolic BP | Theta | 120 | 10.3419 | 6.9064 | 4.2601 |
| External test set (eICU) | Diastolic BP | Transformer | 5 | 5.0376 | 2.6996 | 1.6670 |
| External test set (eICU) | Diastolic BP | Transformer | 10 | 5.7195 | 3.3150 | 2.0458 |
| External test set (eICU) | Diastolic BP | Transformer | 15 | 6.0371 | 3.6372 | 2.2450 |
| External test set (eICU) | Diastolic BP | Transformer | 20 | 6.3145 | 3.9007 | 2.4078 |
| External test set (eICU) | Diastolic BP | Transformer | 25 | 6.5303 | 4.1096 | 2.5369 |
| External test set (eICU) | Diastolic BP | Transformer | 30 | 6.7129 | 4.2855 | 2.6455 |
| External test set (eICU) | Diastolic BP | Transformer | 35 | 6.8979 | 4.4501 | 2.7474 |
| External test set (eICU) | Diastolic BP | Transformer | 40 | 7.0120 | 4.5666 | 2.8194 |
| External test set (eICU) | Diastolic BP | Transformer | 45 | 7.0714 | 4.6444 | 2.8674 |
| External test set (eICU) | Diastolic BP | Transformer | 50 | 7.1833 | 4.7469 | 2.9308 |
| External test set (eICU) | Diastolic BP | Transformer | 55 | 7.2764 | 4.8341 | 2.9848 |
| External test set (eICU) | Diastolic BP | Transformer | 60 | 7.3611 | 4.9126 | 3.0332 |
| External test set (eICU) | Diastolic BP | Transformer | 65 | 7.4848 | 5.0107 | 3.0942 |
| External test set (eICU) | Diastolic BP | Transformer | 70 | 7.5482 | 5.0715 | 3.1317 |
| External test set (eICU) | Diastolic BP | Transformer | 75 | 7.5949 | 5.1198 | 3.1614 |
| External test set (eICU) | Diastolic BP | Transformer | 80 | 7.6569 | 5.1765 | 3.1965 |
| External test set (eICU) | Diastolic BP | Transformer | 85 | 7.7102 | 5.2258 | 3.2268 |
| External test set (eICU) | Diastolic BP | Transformer | 90 | 7.7598 | 5.2714 | 3.2548 |
| External test set (eICU) | Diastolic BP | Transformer | 95 | 7.8246 | 5.3256 | 3.2883 |
| External test set (eICU) | Diastolic BP | Transformer | 100 | 7.8681 | 5.3651 | 3.3125 |
| External test set (eICU) | Diastolic BP | Transformer | 105 | 7.8866 | 5.3889 | 3.3270 |
| External test set (eICU) | Diastolic BP | Transformer | 110 | 7.9325 | 5.4292 | 3.3520 |
| External test set (eICU) | Diastolic BP | Transformer | 115 | 7.9731 | 5.4663 | 3.3749 |
| External test set (eICU) | Diastolic BP | Transformer | 120 | 8.0147 | 5.5026 | 3.3973 |
| External test set (eICU) | Heart rate | AR NNet | 5 | 3.4236 | 2.0317 | 1.2708 |
| External test set (eICU) | Heart rate | AR NNet | 10 | 4.0096 | 2.4119 | 1.5080 |
| External test set (eICU) | Heart rate | AR NNet | 15 | 4.3922 | 2.6775 | 1.6739 |
| External test set (eICU) | Heart rate | AR NNet | 20 | 4.6892 | 2.8957 | 1.8102 |
| External test set (eICU) | Heart rate | AR NNet | 25 | 4.9466 | 3.0885 | 1.9307 |
| External test set (eICU) | Heart rate | AR NNet | 30 | 5.1776 | 3.2658 | 2.0414 |
| External test set (eICU) | Heart rate | AR NNet | 35 | 5.3930 | 3.4355 | 2.1476 |
| External test set (eICU) | Heart rate | AR NNet | 40 | 5.5782 | 3.5792 | 2.2375 |
| External test set (eICU) | Heart rate | AR NNet | 45 | 5.7491 | 3.7119 | 2.3205 |
| External test set (eICU) | Heart rate | AR NNet | 50 | 5.9072 | 3.8356 | 2.3979 |
| External test set (eICU) | Heart rate | AR NNet | 55 | 6.0560 | 3.9524 | 2.4712 |
| External test set (eICU) | Heart rate | AR NNet | 60 | 6.1954 | 4.0627 | 2.5403 |
| External test set (eICU) | Heart rate | AR NNet | 65 | 6.3307 | 4.1686 | 2.6066 |
| External test set (eICU) | Heart rate | AR NNet | 70 | 6.4594 | 4.2694 | 2.6696 |
| External test set (eICU) | Heart rate | AR NNet | 75 | 6.5834 | 4.3664 | 2.7303 |
| External test set (eICU) | Heart rate | AR NNet | 80 | 6.7015 | 4.4591 | 2.7883 |
| External test set (eICU) | Heart rate | AR NNet | 85 | 6.8149 | 4.5484 | 2.8442 |
| External test set (eICU) | Heart rate | AR NNet | 90 | 6.9211 | 4.6334 | 2.8975 |
| External test set (eICU) | Heart rate | AR NNet | 95 | 7.0251 | 4.7157 | 2.9490 |
| External test set (eICU) | Heart rate | AR NNet | 100 | 7.1236 | 4.7947 | 2.9984 |
| External test set (eICU) | Heart rate | AR NNet | 105 | 7.2205 | 4.8715 | 3.0465 |
| External test set (eICU) | Heart rate | AR NNet | 110 | 7.3149 | 4.9460 | 3.0932 |
| External test set (eICU) | Heart rate | AR NNet | 115 | 7.4054 | 5.0183 | 3.1384 |
| External test set (eICU) | Heart rate | AR NNet | 120 | 7.4921 | 5.0878 | 3.1820 |
| External test set (eICU) | Heart rate | ARIMA | 5 | 3.1485 | 1.6690 | 1.0432 |
| External test set (eICU) | Heart rate | ARIMA | 10 | 3.9245 | 2.1792 | 1.3615 |
| External test set (eICU) | Heart rate | ARIMA | 15 | 4.3895 | 2.5005 | 1.5621 |
| External test set (eICU) | Heart rate | ARIMA | 20 | 4.7414 | 2.7496 | 1.7177 |
| External test set (eICU) | Heart rate | ARIMA | 25 | 5.0398 | 2.9675 | 1.8538 |
| External test set (eICU) | Heart rate | ARIMA | 30 | 5.3119 | 3.1597 | 1.9738 |
| External test set (eICU) | Heart rate | ARIMA | 35 | 5.5562 | 3.3365 | 2.0842 |
| External test set (eICU) | Heart rate | ARIMA | 40 | 5.7772 | 3.4977 | 2.1850 |
| External test set (eICU) | Heart rate | ARIMA | 45 | 5.9822 | 3.6498 | 2.2803 |
| External test set (eICU) | Heart rate | ARIMA | 50 | 6.1801 | 3.7927 | 2.3696 |
| External test set (eICU) | Heart rate | ARIMA | 55 | 6.3646 | 3.9301 | 2.4557 |
| External test set (eICU) | Heart rate | ARIMA | 60 | 6.5424 | 4.0598 | 2.5370 |
| External test set (eICU) | Heart rate | ARIMA | 65 | 6.7171 | 4.1874 | 2.6169 |
| External test set (eICU) | Heart rate | ARIMA | 70 | 6.8832 | 4.3084 | 2.6926 |
| External test set (eICU) | Heart rate | ARIMA | 75 | 7.0458 | 4.4274 | 2.7672 |
| External test set (eICU) | Heart rate | ARIMA | 80 | 7.2030 | 4.5408 | 2.8381 |
| External test set (eICU) | Heart rate | ARIMA | 85 | 7.3619 | 4.6540 | 2.9089 |
| External test set (eICU) | Heart rate | ARIMA | 90 | 7.5119 | 4.7618 | 2.9765 |
| External test set (eICU) | Heart rate | ARIMA | 95 | 7.6617 | 4.8675 | 3.0429 |
| External test set (eICU) | Heart rate | ARIMA | 100 | 7.8051 | 4.9699 | 3.1070 |
| External test set (eICU) | Heart rate | ARIMA | 105 | 7.9481 | 5.0709 | 3.1702 |
| External test set (eICU) | Heart rate | ARIMA | 110 | 8.0879 | 5.1698 | 3.2322 |
| External test set (eICU) | Heart rate | ARIMA | 115 | 8.2289 | 5.2670 | 3.2931 |
| External test set (eICU) | Heart rate | ARIMA | 120 | 8.3647 | 5.3617 | 3.3524 |
| External test set (eICU) | Heart rate | ETS | 5 | 3.1124 | 1.6109 | 1.0069 |
| External test set (eICU) | Heart rate | ETS | 10 | 3.9133 | 2.0999 | 1.3119 |
| External test set (eICU) | Heart rate | ETS | 15 | 4.4325 | 2.4220 | 1.5130 |
| External test set (eICU) | Heart rate | ETS | 20 | 4.8435 | 2.6795 | 1.6736 |
| External test set (eICU) | Heart rate | ETS | 25 | 5.1942 | 2.9012 | 1.8120 |
| External test set (eICU) | Heart rate | ETS | 30 | 5.5100 | 3.0983 | 1.9350 |
| External test set (eICU) | Heart rate | ETS | 35 | 5.7940 | 3.2771 | 2.0465 |
| External test set (eICU) | Heart rate | ETS | 40 | 6.0555 | 3.4414 | 2.1491 |
| External test set (eICU) | Heart rate | ETS | 45 | 6.2964 | 3.5937 | 2.2442 |
| External test set (eICU) | Heart rate | ETS | 50 | 6.5273 | 3.7372 | 2.3338 |
| External test set (eICU) | Heart rate | ETS | 55 | 6.7459 | 3.8743 | 2.4195 |
| External test set (eICU) | Heart rate | ETS | 60 | 6.9555 | 4.0038 | 2.5005 |
| External test set (eICU) | Heart rate | ETS | 65 | 7.1597 | 4.1295 | 2.5791 |
| External test set (eICU) | Heart rate | ETS | 70 | 7.3549 | 4.2496 | 2.6540 |
| External test set (eICU) | Heart rate | ETS | 75 | 7.5433 | 4.3659 | 2.7267 |
| External test set (eICU) | Heart rate | ETS | 80 | 7.7244 | 4.4782 | 2.7968 |
| External test set (eICU) | Heart rate | ETS | 85 | 7.9035 | 4.5884 | 2.8656 |
| External test set (eICU) | Heart rate | ETS | 90 | 8.0737 | 4.6932 | 2.9313 |
| External test set (eICU) | Heart rate | ETS | 95 | 8.2475 | 4.7954 | 2.9953 |
| External test set (eICU) | Heart rate | ETS | 100 | 8.4134 | 4.8931 | 3.0563 |
| External test set (eICU) | Heart rate | ETS | 105 | 8.5744 | 4.9897 | 3.1168 |
| External test set (eICU) | Heart rate | ETS | 110 | 8.7350 | 5.0833 | 3.1753 |
| External test set (eICU) | Heart rate | ETS | 115 | 8.8971 | 5.1754 | 3.2329 |
| External test set (eICU) | Heart rate | ETS | 120 | 9.0515 | 5.2644 | 3.2885 |
| External test set (eICU) | Heart rate | GRU | 5 | 3.1085 | 1.7923 | 1.1195 |
| External test set (eICU) | Heart rate | GRU | 10 | 3.7956 | 2.2452 | 1.4031 |
| External test set (eICU) | Heart rate | GRU | 15 | 4.2133 | 2.5414 | 1.5889 |
| External test set (eICU) | Heart rate | GRU | 20 | 4.5340 | 2.7864 | 1.7427 |
| External test set (eICU) | Heart rate | GRU | 25 | 4.8043 | 2.9990 | 1.8760 |
| External test set (eICU) | Heart rate | GRU | 30 | 5.0461 | 3.1892 | 1.9956 |
| External test set (eICU) | Heart rate | GRU | 35 | 5.2556 | 3.3611 | 2.1032 |
| External test set (eICU) | Heart rate | GRU | 40 | 5.4408 | 3.5148 | 2.2000 |
| External test set (eICU) | Heart rate | GRU | 45 | 5.6162 | 3.6603 | 2.2913 |
| External test set (eICU) | Heart rate | GRU | 50 | 5.7754 | 3.7963 | 2.3769 |
| External test set (eICU) | Heart rate | GRU | 55 | 5.9209 | 3.9138 | 2.4504 |
| External test set (eICU) | Heart rate | GRU | 60 | 6.0668 | 4.0388 | 2.5292 |
| External test set (eICU) | Heart rate | GRU | 65 | 6.1997 | 4.1506 | 2.5995 |
| External test set (eICU) | Heart rate | GRU | 70 | 6.3295 | 4.2693 | 2.6741 |
| External test set (eICU) | Heart rate | GRU | 75 | 6.4499 | 4.3723 | 2.7390 |
| External test set (eICU) | Heart rate | GRU | 80 | 6.5724 | 4.4808 | 2.8073 |
| External test set (eICU) | Heart rate | GRU | 85 | 6.6856 | 4.5778 | 2.8682 |
| External test set (eICU) | Heart rate | GRU | 90 | 6.7900 | 4.6709 | 2.9271 |
| External test set (eICU) | Heart rate | GRU | 95 | 6.8895 | 4.7543 | 2.9789 |
| External test set (eICU) | Heart rate | GRU | 100 | 6.9971 | 4.8544 | 3.0428 |
| External test set (eICU) | Heart rate | GRU | 105 | 7.0957 | 4.9414 | 3.0975 |
| External test set (eICU) | Heart rate | GRU | 110 | 7.1969 | 5.0339 | 3.1554 |
| External test set (eICU) | Heart rate | GRU | 115 | 7.2793 | 5.1026 | 3.1991 |
| External test set (eICU) | Heart rate | GRU | 120 | 7.3596 | 5.1774 | 3.2459 |
| External test set (eICU) | Heart rate | Naive | 5 | 3.1283 | 1.5481 | 0.9675 |
| External test set (eICU) | Heart rate | Naive | 10 | 3.9597 | 2.0839 | 1.3017 |
| External test set (eICU) | Heart rate | Naive | 15 | 4.4426 | 2.4151 | 1.5087 |
| External test set (eICU) | Heart rate | Naive | 20 | 4.7899 | 2.6707 | 1.6681 |
| External test set (eICU) | Heart rate | Naive | 25 | 5.0782 | 2.8870 | 1.8032 |
| External test set (eICU) | Heart rate | Naive | 30 | 5.3294 | 3.0765 | 1.9215 |
| External test set (eICU) | Heart rate | Naive | 35 | 5.5536 | 3.2470 | 2.0279 |
| External test set (eICU) | Heart rate | Naive | 40 | 5.7537 | 3.4018 | 2.1245 |
| External test set (eICU) | Heart rate | Naive | 45 | 5.9339 | 3.5438 | 2.2132 |
| External test set (eICU) | Heart rate | Naive | 50 | 6.1041 | 3.6764 | 2.2959 |
| External test set (eICU) | Heart rate | Naive | 55 | 6.2626 | 3.8026 | 2.3747 |
| External test set (eICU) | Heart rate | Naive | 60 | 6.4112 | 3.9195 | 2.4479 |
| External test set (eICU) | Heart rate | Naive | 65 | 6.5530 | 4.0333 | 2.5190 |
| External test set (eICU) | Heart rate | Naive | 70 | 6.6845 | 4.1408 | 2.5860 |
| External test set (eICU) | Heart rate | Naive | 75 | 6.8131 | 4.2453 | 2.6514 |
| External test set (eICU) | Heart rate | Naive | 80 | 6.9370 | 4.3461 | 2.7143 |
| External test set (eICU) | Heart rate | Naive | 85 | 7.0567 | 4.4437 | 2.7753 |
| External test set (eICU) | Heart rate | Naive | 90 | 7.1659 | 4.5353 | 2.8326 |
| External test set (eICU) | Heart rate | Naive | 95 | 7.2778 | 4.6250 | 2.8888 |
| External test set (eICU) | Heart rate | Naive | 100 | 7.3786 | 4.7098 | 2.9418 |
| External test set (eICU) | Heart rate | Naive | 105 | 7.4779 | 4.7928 | 2.9936 |
| External test set (eICU) | Heart rate | Naive | 110 | 7.5764 | 4.8733 | 3.0440 |
| External test set (eICU) | Heart rate | Naive | 115 | 7.6712 | 4.9508 | 3.0925 |
| External test set (eICU) | Heart rate | Naive | 120 | 7.7580 | 5.0246 | 3.1387 |
| External test set (eICU) | Heart rate | Theta | 5 | 3.0871 | 1.6118 | 1.0075 |
| External test set (eICU) | Heart rate | Theta | 10 | 3.8624 | 2.1082 | 1.3173 |
| External test set (eICU) | Heart rate | Theta | 15 | 4.3435 | 2.4355 | 1.5217 |
| External test set (eICU) | Heart rate | Theta | 20 | 4.7126 | 2.6988 | 1.6860 |
| External test set (eICU) | Heart rate | Theta | 25 | 5.0262 | 2.9269 | 1.8285 |
| External test set (eICU) | Heart rate | Theta | 30 | 5.3063 | 3.1311 | 1.9560 |
| External test set (eICU) | Heart rate | Theta | 35 | 5.5619 | 3.3183 | 2.0729 |
| External test set (eICU) | Heart rate | Theta | 40 | 5.7965 | 3.4914 | 2.1812 |
| External test set (eICU) | Heart rate | Theta | 45 | 6.0172 | 3.6537 | 2.2826 |
| External test set (eICU) | Heart rate | Theta | 50 | 6.2272 | 3.8085 | 2.3794 |
| External test set (eICU) | Heart rate | Theta | 55 | 6.4271 | 3.9569 | 2.4722 |
| External test set (eICU) | Heart rate | Theta | 60 | 6.6204 | 4.0990 | 2.5612 |
| External test set (eICU) | Heart rate | Theta | 65 | 6.8079 | 4.2376 | 2.6479 |
| External test set (eICU) | Heart rate | Theta | 70 | 6.9886 | 4.3709 | 2.7313 |
| External test set (eICU) | Heart rate | Theta | 75 | 7.1662 | 4.5019 | 2.8132 |
| External test set (eICU) | Heart rate | Theta | 80 | 7.3419 | 4.6302 | 2.8935 |
| External test set (eICU) | Heart rate | Theta | 85 | 7.5144 | 4.7564 | 2.9724 |
| External test set (eICU) | Heart rate | Theta | 90 | 7.6818 | 4.8785 | 3.0490 |
| External test set (eICU) | Heart rate | Theta | 95 | 7.8494 | 4.9984 | 3.1242 |
| External test set (eICU) | Heart rate | Theta | 100 | 8.0091 | 5.1153 | 3.1975 |
| External test set (eICU) | Heart rate | Theta | 105 | 8.1696 | 5.2308 | 3.2698 |
| External test set (eICU) | Heart rate | Theta | 110 | 8.3302 | 5.3440 | 3.3408 |
| External test set (eICU) | Heart rate | Theta | 115 | 8.4878 | 5.4553 | 3.4106 |
| External test set (eICU) | Heart rate | Theta | 120 | 8.6418 | 5.5650 | 3.4793 |
| External test set (eICU) | Heart rate | Transformer | 5 | 3.2178 | 1.9715 | 1.2333 |
| External test set (eICU) | Heart rate | Transformer | 10 | 3.8949 | 2.4006 | 1.5011 |
| External test set (eICU) | Heart rate | Transformer | 15 | 4.3039 | 2.6817 | 1.6769 |
| External test set (eICU) | Heart rate | Transformer | 20 | 4.6100 | 2.9066 | 1.8175 |
| External test set (eICU) | Heart rate | Transformer | 25 | 4.8713 | 3.1047 | 1.9417 |
| External test set (eICU) | Heart rate | Transformer | 30 | 5.1051 | 3.2857 | 2.0551 |
| External test set (eICU) | Heart rate | Transformer | 35 | 5.3033 | 3.4445 | 2.1546 |
| External test set (eICU) | Heart rate | Transformer | 40 | 5.4831 | 3.5909 | 2.2465 |
| External test set (eICU) | Heart rate | Transformer | 45 | 5.6477 | 3.7262 | 2.3315 |
| External test set (eICU) | Heart rate | Transformer | 50 | 5.8029 | 3.8552 | 2.4124 |
| External test set (eICU) | Heart rate | Transformer | 55 | 5.9483 | 3.9768 | 2.4889 |
| External test set (eICU) | Heart rate | Transformer | 60 | 6.0858 | 4.0922 | 2.5616 |
| External test set (eICU) | Heart rate | Transformer | 65 | 6.2185 | 4.2068 | 2.6338 |
| External test set (eICU) | Heart rate | Transformer | 70 | 6.3432 | 4.3167 | 2.7029 |
| External test set (eICU) | Heart rate | Transformer | 75 | 6.4635 | 4.4218 | 2.7691 |
| External test set (eICU) | Heart rate | Transformer | 80 | 6.5799 | 4.5236 | 2.8332 |
| External test set (eICU) | Heart rate | Transformer | 85 | 6.6914 | 4.6214 | 2.8948 |
| External test set (eICU) | Heart rate | Transformer | 90 | 6.7964 | 4.7154 | 2.9540 |
| External test set (eICU) | Heart rate | Transformer | 95 | 6.8995 | 4.8042 | 3.0098 |
| External test set (eICU) | Heart rate | Transformer | 100 | 6.9976 | 4.8909 | 3.0643 |
| External test set (eICU) | Heart rate | Transformer | 105 | 7.0935 | 4.9754 | 3.1174 |
| External test set (eICU) | Heart rate | Transformer | 110 | 7.1873 | 5.0582 | 3.1697 |
| External test set (eICU) | Heart rate | Transformer | 115 | 7.2767 | 5.1378 | 3.2198 |
| External test set (eICU) | Heart rate | Transformer | 120 | 7.3598 | 5.2123 | 3.2668 |
| External test set (eICU) | Mean BP | AR NNet | 5 | 6.3812 | 3.8390 | 2.3580 |
| External test set (eICU) | Mean BP | AR NNet | 10 | 7.1401 | 4.4907 | 2.7568 |
| External test set (eICU) | Mean BP | AR NNet | 15 | 7.5626 | 4.8818 | 2.9963 |
| External test set (eICU) | Mean BP | AR NNet | 20 | 7.8914 | 5.1843 | 3.1820 |
| External test set (eICU) | Mean BP | AR NNet | 25 | 8.1417 | 5.4213 | 3.3275 |
| External test set (eICU) | Mean BP | AR NNet | 30 | 8.3505 | 5.6210 | 3.4503 |
| External test set (eICU) | Mean BP | AR NNet | 35 | 8.5520 | 5.8068 | 3.5644 |
| External test set (eICU) | Mean BP | AR NNet | 40 | 8.7039 | 5.9497 | 3.6521 |
| External test set (eICU) | Mean BP | AR NNet | 45 | 8.8354 | 6.0720 | 3.7272 |
| External test set (eICU) | Mean BP | AR NNet | 50 | 8.9618 | 6.1884 | 3.7988 |
| External test set (eICU) | Mean BP | AR NNet | 55 | 9.0689 | 6.2881 | 3.8601 |
| External test set (eICU) | Mean BP | AR NNet | 60 | 9.1701 | 6.3811 | 3.9174 |
| External test set (eICU) | Mean BP | AR NNet | 65 | 9.2802 | 6.4755 | 3.9756 |
| External test set (eICU) | Mean BP | AR NNet | 70 | 9.3604 | 6.5498 | 4.0213 |
| External test set (eICU) | Mean BP | AR NNet | 75 | 9.4342 | 6.6176 | 4.0630 |
| External test set (eICU) | Mean BP | AR NNet | 80 | 9.5105 | 6.6856 | 4.1049 |
| External test set (eICU) | Mean BP | AR NNet | 85 | 9.5755 | 6.7449 | 4.1414 |
| External test set (eICU) | Mean BP | AR NNet | 90 | 9.6431 | 6.8037 | 4.1776 |
| External test set (eICU) | Mean BP | AR NNet | 95 | 9.7117 | 6.8629 | 4.2141 |
| External test set (eICU) | Mean BP | AR NNet | 100 | 9.7683 | 6.9146 | 4.2458 |
| External test set (eICU) | Mean BP | AR NNet | 105 | 9.8221 | 6.9628 | 4.2755 |
| External test set (eICU) | Mean BP | AR NNet | 110 | 9.8836 | 7.0159 | 4.3080 |
| External test set (eICU) | Mean BP | AR NNet | 115 | 9.9365 | 7.0627 | 4.3370 |
| External test set (eICU) | Mean BP | AR NNet | 120 | 9.9937 | 7.1117 | 4.3672 |
| External test set (eICU) | Mean BP | ARIMA | 5 | 6.2902 | 3.4441 | 2.1139 |
| External test set (eICU) | Mean BP | ARIMA | 10 | 7.2752 | 4.3774 | 2.6864 |
| External test set (eICU) | Mean BP | ARIMA | 15 | 7.7689 | 4.8666 | 2.9867 |
| External test set (eICU) | Mean BP | ARIMA | 20 | 8.1568 | 5.2321 | 3.2112 |
| External test set (eICU) | Mean BP | ARIMA | 25 | 8.4648 | 5.5217 | 3.3891 |
| External test set (eICU) | Mean BP | ARIMA | 30 | 8.7260 | 5.7595 | 3.5354 |
| External test set (eICU) | Mean BP | ARIMA | 35 | 8.9818 | 5.9817 | 3.6719 |
| External test set (eICU) | Mean BP | ARIMA | 40 | 9.1883 | 6.1611 | 3.7823 |
| External test set (eICU) | Mean BP | ARIMA | 45 | 9.3649 | 6.3121 | 3.8751 |
| External test set (eICU) | Mean BP | ARIMA | 50 | 9.5416 | 6.4615 | 3.9671 |
| External test set (eICU) | Mean BP | ARIMA | 55 | 9.7004 | 6.5929 | 4.0479 |
| External test set (eICU) | Mean BP | ARIMA | 60 | 9.8457 | 6.7114 | 4.1209 |
| External test set (eICU) | Mean BP | ARIMA | 65 | 10.0077 | 6.8395 | 4.1996 |
| External test set (eICU) | Mean BP | ARIMA | 70 | 10.1345 | 6.9387 | 4.2606 |
| External test set (eICU) | Mean BP | ARIMA | 75 | 10.2480 | 7.0274 | 4.3152 |
| External test set (eICU) | Mean BP | ARIMA | 80 | 10.3630 | 7.1165 | 4.3702 |
| External test set (eICU) | Mean BP | ARIMA | 85 | 10.4678 | 7.1961 | 4.4190 |
| External test set (eICU) | Mean BP | ARIMA | 90 | 10.5719 | 7.2709 | 4.4650 |
| External test set (eICU) | Mean BP | ARIMA | 95 | 10.6850 | 7.3520 | 4.5151 |
| External test set (eICU) | Mean BP | ARIMA | 100 | 10.7814 | 7.4197 | 4.5570 |
| External test set (eICU) | Mean BP | ARIMA | 105 | 10.8704 | 7.4805 | 4.5945 |
| External test set (eICU) | Mean BP | ARIMA | 110 | 10.9666 | 7.5484 | 4.6362 |
| External test set (eICU) | Mean BP | ARIMA | 115 | 11.0576 | 7.6107 | 4.6746 |
| External test set (eICU) | Mean BP | ARIMA | 120 | 11.1450 | 7.6728 | 4.7130 |
| External test set (eICU) | Mean BP | ETS | 5 | 6.3979 | 3.3967 | 2.0849 |
| External test set (eICU) | Mean BP | ETS | 10 | 7.4789 | 4.3035 | 2.6409 |
| External test set (eICU) | Mean BP | ETS | 15 | 8.0620 | 4.8323 | 2.9651 |
| External test set (eICU) | Mean BP | ETS | 20 | 8.5459 | 5.2609 | 3.2282 |
| External test set (eICU) | Mean BP | ETS | 25 | 8.9168 | 5.5991 | 3.4362 |
| External test set (eICU) | Mean BP | ETS | 30 | 9.2269 | 5.8820 | 3.6098 |
| External test set (eICU) | Mean BP | ETS | 35 | 9.5562 | 6.1629 | 3.7824 |
| External test set (eICU) | Mean BP | ETS | 40 | 9.7834 | 6.3682 | 3.9082 |
| External test set (eICU) | Mean BP | ETS | 45 | 9.9447 | 6.5175 | 3.9998 |
| External test set (eICU) | Mean BP | ETS | 50 | 10.1550 | 6.6965 | 4.1098 |
| External test set (eICU) | Mean BP | ETS | 55 | 10.3312 | 6.8480 | 4.2027 |
| External test set (eICU) | Mean BP | ETS | 60 | 10.4798 | 6.9796 | 4.2837 |
| External test set (eICU) | Mean BP | ETS | 65 | 10.7064 | 7.1617 | 4.3956 |
| External test set (eICU) | Mean BP | ETS | 70 | 10.8329 | 7.2699 | 4.4617 |
| External test set (eICU) | Mean BP | ETS | 75 | 10.9431 | 7.3627 | 4.5187 |
| External test set (eICU) | Mean BP | ETS | 80 | 11.0594 | 7.4619 | 4.5798 |
| External test set (eICU) | Mean BP | ETS | 85 | 11.1625 | 7.5468 | 4.6317 |
| External test set (eICU) | Mean BP | ETS | 90 | 11.2619 | 7.6223 | 4.6782 |
| External test set (eICU) | Mean BP | ETS | 95 | 11.3843 | 7.7144 | 4.7351 |
| External test set (eICU) | Mean BP | ETS | 100 | 11.4636 | 7.7751 | 4.7724 |
| External test set (eICU) | Mean BP | ETS | 105 | 11.5188 | 7.8116 | 4.7949 |
| External test set (eICU) | Mean BP | ETS | 110 | 11.6023 | 7.8783 | 4.8358 |
| External test set (eICU) | Mean BP | ETS | 115 | 11.6862 | 7.9385 | 4.8728 |
| External test set (eICU) | Mean BP | ETS | 120 | 11.7605 | 7.9979 | 4.9093 |
| External test set (eICU) | Mean BP | GRU | 5 | 5.9306 | 3.3087 | 2.0382 |
| External test set (eICU) | Mean BP | GRU | 10 | 6.8140 | 4.1450 | 2.5509 |
| External test set (eICU) | Mean BP | GRU | 15 | 7.2550 | 4.5995 | 2.8307 |
| External test set (eICU) | Mean BP | GRU | 20 | 7.6168 | 4.9606 | 3.0535 |
| External test set (eICU) | Mean BP | GRU | 25 | 7.8917 | 5.2423 | 3.2275 |
| External test set (eICU) | Mean BP | GRU | 30 | 8.1348 | 5.4825 | 3.3758 |
| External test set (eICU) | Mean BP | GRU | 35 | 8.3389 | 5.6808 | 3.4981 |
| External test set (eICU) | Mean BP | GRU | 40 | 8.4821 | 5.8317 | 3.5911 |
| External test set (eICU) | Mean BP | GRU | 45 | 8.5974 | 5.9525 | 3.6653 |
| External test set (eICU) | Mean BP | GRU | 50 | 8.7292 | 6.0768 | 3.7419 |
| External test set (eICU) | Mean BP | GRU | 55 | 8.8411 | 6.1871 | 3.8101 |
| External test set (eICU) | Mean BP | GRU | 60 | 8.9478 | 6.2874 | 3.8721 |
| External test set (eICU) | Mean BP | GRU | 65 | 9.0594 | 6.3828 | 3.9309 |
| External test set (eICU) | Mean BP | GRU | 70 | 9.1356 | 6.4594 | 3.9779 |
| External test set (eICU) | Mean BP | GRU | 75 | 9.1984 | 6.5192 | 4.0143 |
| External test set (eICU) | Mean BP | GRU | 80 | 9.2719 | 6.5925 | 4.0596 |
| External test set (eICU) | Mean BP | GRU | 85 | 9.3320 | 6.6494 | 4.0945 |
| External test set (eICU) | Mean BP | GRU | 90 | 9.3918 | 6.7065 | 4.1297 |
| External test set (eICU) | Mean BP | GRU | 95 | 9.4490 | 6.7571 | 4.1606 |
| External test set (eICU) | Mean BP | GRU | 100 | 9.4995 | 6.8026 | 4.1882 |
| External test set (eICU) | Mean BP | GRU | 105 | 9.5364 | 6.8408 | 4.2112 |
| External test set (eICU) | Mean BP | GRU | 110 | 9.5838 | 6.8856 | 4.2390 |
| External test set (eICU) | Mean BP | GRU | 115 | 9.6253 | 6.9215 | 4.2610 |
| External test set (eICU) | Mean BP | GRU | 120 | 9.6721 | 6.9637 | 4.2869 |
| External test set (eICU) | Mean BP | Naive | 5 | 6.5488 | 3.2924 | 2.0211 |
| External test set (eICU) | Mean BP | Naive | 10 | 7.7828 | 4.3414 | 2.6631 |
| External test set (eICU) | Mean BP | Naive | 15 | 8.3259 | 4.8818 | 2.9944 |
| External test set (eICU) | Mean BP | Naive | 20 | 8.7765 | 5.3157 | 3.2602 |
| External test set (eICU) | Mean BP | Naive | 25 | 9.1196 | 5.6582 | 3.4708 |
| External test set (eICU) | Mean BP | Naive | 30 | 9.4073 | 5.9420 | 3.6451 |
| External test set (eICU) | Mean BP | Naive | 35 | 9.7255 | 6.2334 | 3.8239 |
| External test set (eICU) | Mean BP | Naive | 40 | 9.9409 | 6.4416 | 3.9516 |
| External test set (eICU) | Mean BP | Naive | 45 | 10.0339 | 6.5630 | 4.0258 |
| External test set (eICU) | Mean BP | Naive | 50 | 10.2086 | 6.7331 | 4.1304 |
| External test set (eICU) | Mean BP | Naive | 55 | 10.3589 | 6.8802 | 4.2206 |
| External test set (eICU) | Mean BP | Naive | 60 | 10.4818 | 7.0057 | 4.2979 |
| External test set (eICU) | Mean BP | Naive | 65 | 10.6878 | 7.1887 | 4.4103 |
| External test set (eICU) | Mean BP | Naive | 70 | 10.7933 | 7.2944 | 4.4752 |
| External test set (eICU) | Mean BP | Naive | 75 | 10.8537 | 7.3650 | 4.5181 |
| External test set (eICU) | Mean BP | Naive | 80 | 10.9375 | 7.4542 | 4.5730 |
| External test set (eICU) | Mean BP | Naive | 85 | 11.0079 | 7.5305 | 4.6197 |
| External test set (eICU) | Mean BP | Naive | 90 | 11.0809 | 7.5983 | 4.6615 |
| External test set (eICU) | Mean BP | Naive | 95 | 11.1815 | 7.6890 | 4.7174 |
| External test set (eICU) | Mean BP | Naive | 100 | 11.2472 | 7.7504 | 4.7552 |
| External test set (eICU) | Mean BP | Naive | 105 | 11.2416 | 7.7599 | 4.7610 |
| External test set (eICU) | Mean BP | Naive | 110 | 11.2938 | 7.8163 | 4.7953 |
| External test set (eICU) | Mean BP | Naive | 115 | 11.3457 | 7.8674 | 4.8264 |
| External test set (eICU) | Mean BP | Naive | 120 | 11.4040 | 7.9208 | 4.8593 |
| External test set (eICU) | Mean BP | Theta | 5 | 6.4005 | 3.4005 | 2.0870 |
| External test set (eICU) | Mean BP | Theta | 10 | 7.4868 | 4.3207 | 2.6515 |
| External test set (eICU) | Mean BP | Theta | 15 | 8.0776 | 4.8629 | 2.9842 |
| External test set (eICU) | Mean BP | Theta | 20 | 8.5749 | 5.3091 | 3.2583 |
| External test set (eICU) | Mean BP | Theta | 25 | 8.9665 | 5.6684 | 3.4795 |
| External test set (eICU) | Mean BP | Theta | 30 | 9.3005 | 5.9730 | 3.6666 |
| External test set (eICU) | Mean BP | Theta | 35 | 9.6618 | 6.2820 | 3.8565 |
| External test set (eICU) | Mean BP | Theta | 40 | 9.9184 | 6.5132 | 3.9985 |
| External test set (eICU) | Mean BP | Theta | 45 | 10.1180 | 6.6917 | 4.1083 |
| External test set (eICU) | Mean BP | Theta | 50 | 10.3707 | 6.9023 | 4.2380 |
| External test set (eICU) | Mean BP | Theta | 55 | 10.5860 | 7.0851 | 4.3502 |
| External test set (eICU) | Mean BP | Theta | 60 | 10.7808 | 7.2484 | 4.4509 |
| External test set (eICU) | Mean BP | Theta | 65 | 11.0600 | 7.4688 | 4.5864 |
| External test set (eICU) | Mean BP | Theta | 70 | 11.2379 | 7.6130 | 4.6750 |
| External test set (eICU) | Mean BP | Theta | 75 | 11.4029 | 7.7441 | 4.7556 |
| External test set (eICU) | Mean BP | Theta | 80 | 11.5834 | 7.8837 | 4.8417 |
| External test set (eICU) | Mean BP | Theta | 85 | 11.7467 | 8.0081 | 4.9182 |
| External test set (eICU) | Mean BP | Theta | 90 | 11.9037 | 8.1228 | 4.9889 |
| External test set (eICU) | Mean BP | Theta | 95 | 12.0932 | 8.2585 | 5.0727 |
| External test set (eICU) | Mean BP | Theta | 100 | 12.2395 | 8.3629 | 5.1373 |
| External test set (eICU) | Mean BP | Theta | 105 | 12.3688 | 8.4465 | 5.1890 |
| External test set (eICU) | Mean BP | Theta | 110 | 12.5305 | 8.5600 | 5.2592 |
| External test set (eICU) | Mean BP | Theta | 115 | 12.6803 | 8.6642 | 5.3233 |
| External test set (eICU) | Mean BP | Theta | 120 | 12.8295 | 8.7694 | 5.3881 |
| External test set (eICU) | Mean BP | Transformer | 5 | 6.0415 | 3.3143 | 2.0378 |
| External test set (eICU) | Mean BP | Transformer | 10 | 6.9449 | 4.1683 | 2.5618 |
| External test set (eICU) | Mean BP | Transformer | 15 | 7.3539 | 4.6096 | 2.8331 |
| External test set (eICU) | Mean BP | Transformer | 20 | 7.7020 | 4.9634 | 3.0509 |
| External test set (eICU) | Mean BP | Transformer | 25 | 7.9756 | 5.2420 | 3.2224 |
| External test set (eICU) | Mean BP | Transformer | 30 | 8.2055 | 5.4727 | 3.3644 |
| External test set (eICU) | Mean BP | Transformer | 35 | 8.4255 | 5.6833 | 3.4943 |
| External test set (eICU) | Mean BP | Transformer | 40 | 8.5665 | 5.8320 | 3.5859 |
| External test set (eICU) | Mean BP | Transformer | 45 | 8.6484 | 5.9333 | 3.6481 |
| External test set (eICU) | Mean BP | Transformer | 50 | 8.7763 | 6.0580 | 3.7250 |
| External test set (eICU) | Mean BP | Transformer | 55 | 8.8834 | 6.1644 | 3.7908 |
| External test set (eICU) | Mean BP | Transformer | 60 | 8.9800 | 6.2586 | 3.8488 |
| External test set (eICU) | Mean BP | Transformer | 65 | 9.1105 | 6.3728 | 3.9199 |
| External test set (eICU) | Mean BP | Transformer | 70 | 9.1812 | 6.4446 | 3.9640 |
| External test set (eICU) | Mean BP | Transformer | 75 | 9.2341 | 6.5019 | 3.9992 |
| External test set (eICU) | Mean BP | Transformer | 80 | 9.3014 | 6.5673 | 4.0395 |
| External test set (eICU) | Mean BP | Transformer | 85 | 9.3601 | 6.6244 | 4.0746 |
| External test set (eICU) | Mean BP | Transformer | 90 | 9.4139 | 6.6769 | 4.1068 |
| External test set (eICU) | Mean BP | Transformer | 95 | 9.4791 | 6.7360 | 4.1435 |
| External test set (eICU) | Mean BP | Transformer | 100 | 9.5247 | 6.7809 | 4.1710 |
| External test set (eICU) | Mean BP | Transformer | 105 | 9.5466 | 6.8077 | 4.1872 |
| External test set (eICU) | Mean BP | Transformer | 110 | 9.5920 | 6.8512 | 4.2142 |
| External test set (eICU) | Mean BP | Transformer | 115 | 9.6332 | 6.8912 | 4.2390 |
| External test set (eICU) | Mean BP | Transformer | 120 | 9.6753 | 6.9311 | 4.2636 |
| External test set (eICU) | SpO2 | AR NNet | 5 | 1.3312 | 0.7153 | 0.4512 |
| External test set (eICU) | SpO2 | AR NNet | 10 | 1.5063 | 0.8345 | 0.5264 |
| External test set (eICU) | SpO2 | AR NNet | 15 | 1.6078 | 0.9104 | 0.5742 |
| External test set (eICU) | SpO2 | AR NNet | 20 | 1.6807 | 0.9684 | 0.6107 |
| External test set (eICU) | SpO2 | AR NNet | 25 | 1.7382 | 1.0157 | 0.6404 |
| External test set (eICU) | SpO2 | AR NNet | 30 | 1.7870 | 1.0564 | 0.6660 |
| External test set (eICU) | SpO2 | AR NNet | 35 | 1.8315 | 1.0934 | 0.6892 |
| External test set (eICU) | SpO2 | AR NNet | 40 | 1.8689 | 1.1246 | 0.7089 |
| External test set (eICU) | SpO2 | AR NNet | 45 | 1.9019 | 1.1524 | 0.7263 |
| External test set (eICU) | SpO2 | AR NNet | 50 | 1.9304 | 1.1771 | 0.7418 |
| External test set (eICU) | SpO2 | AR NNet | 55 | 1.9567 | 1.1997 | 0.7561 |
| External test set (eICU) | SpO2 | AR NNet | 60 | 1.9800 | 1.2205 | 0.7692 |
| External test set (eICU) | SpO2 | AR NNet | 65 | 2.0028 | 1.2400 | 0.7814 |
| External test set (eICU) | SpO2 | AR NNet | 70 | 2.0240 | 1.2580 | 0.7927 |
| External test set (eICU) | SpO2 | AR NNet | 75 | 2.0433 | 1.2746 | 0.8032 |
| External test set (eICU) | SpO2 | AR NNet | 80 | 2.0613 | 1.2903 | 0.8131 |
| External test set (eICU) | SpO2 | AR NNet | 85 | 2.0777 | 1.3048 | 0.8222 |
| External test set (eICU) | SpO2 | AR NNet | 90 | 2.0936 | 1.3187 | 0.8309 |
| External test set (eICU) | SpO2 | AR NNet | 95 | 2.1083 | 1.3318 | 0.8392 |
| External test set (eICU) | SpO2 | AR NNet | 100 | 2.1222 | 1.3442 | 0.8470 |
| External test set (eICU) | SpO2 | AR NNet | 105 | 2.1357 | 1.3561 | 0.8545 |
| External test set (eICU) | SpO2 | AR NNet | 110 | 2.1485 | 1.3675 | 0.8617 |
| External test set (eICU) | SpO2 | AR NNet | 115 | 2.1611 | 1.3784 | 0.8686 |
| External test set (eICU) | SpO2 | AR NNet | 120 | 2.1734 | 1.3892 | 0.8753 |
| External test set (eICU) | SpO2 | ARIMA | 5 | 1.3315 | 0.6448 | 0.4067 |
| External test set (eICU) | SpO2 | ARIMA | 10 | 1.5422 | 0.7995 | 0.5041 |
| External test set (eICU) | SpO2 | ARIMA | 15 | 1.6615 | 0.8916 | 0.5622 |
| External test set (eICU) | SpO2 | ARIMA | 20 | 1.7497 | 0.9594 | 0.6049 |
| External test set (eICU) | SpO2 | ARIMA | 25 | 1.8238 | 1.0156 | 0.6403 |
| External test set (eICU) | SpO2 | ARIMA | 30 | 1.8825 | 1.0618 | 0.6693 |
| External test set (eICU) | SpO2 | ARIMA | 35 | 1.9356 | 1.1032 | 0.6954 |
| External test set (eICU) | SpO2 | ARIMA | 40 | 1.9827 | 1.1391 | 0.7180 |
| External test set (eICU) | SpO2 | ARIMA | 45 | 2.0250 | 1.1726 | 0.7390 |
| External test set (eICU) | SpO2 | ARIMA | 50 | 2.0613 | 1.2020 | 0.7575 |
| External test set (eICU) | SpO2 | ARIMA | 55 | 2.0953 | 1.2293 | 0.7746 |
| External test set (eICU) | SpO2 | ARIMA | 60 | 2.1260 | 1.2538 | 0.7901 |
| External test set (eICU) | SpO2 | ARIMA | 65 | 2.1548 | 1.2772 | 0.8048 |
| External test set (eICU) | SpO2 | ARIMA | 70 | 2.1816 | 1.2985 | 0.8181 |
| External test set (eICU) | SpO2 | ARIMA | 75 | 2.2050 | 1.3186 | 0.8308 |
| External test set (eICU) | SpO2 | ARIMA | 80 | 2.2283 | 1.3372 | 0.8425 |
| External test set (eICU) | SpO2 | ARIMA | 85 | 2.2509 | 1.3551 | 0.8538 |
| External test set (eICU) | SpO2 | ARIMA | 90 | 2.2722 | 1.3718 | 0.8643 |
| External test set (eICU) | SpO2 | ARIMA | 95 | 2.2942 | 1.3883 | 0.8746 |
| External test set (eICU) | SpO2 | ARIMA | 100 | 2.3152 | 1.4034 | 0.8841 |
| External test set (eICU) | SpO2 | ARIMA | 105 | 2.3337 | 1.4177 | 0.8932 |
| External test set (eICU) | SpO2 | ARIMA | 110 | 2.3517 | 1.4320 | 0.9021 |
| External test set (eICU) | SpO2 | ARIMA | 115 | 2.3689 | 1.4453 | 0.9105 |
| External test set (eICU) | SpO2 | ARIMA | 120 | 2.3867 | 1.4584 | 0.9188 |
| External test set (eICU) | SpO2 | ETS | 5 | 1.3262 | 0.6153 | 0.3881 |
| External test set (eICU) | SpO2 | ETS | 10 | 1.5661 | 0.7645 | 0.4822 |
| External test set (eICU) | SpO2 | ETS | 15 | 1.7132 | 0.8611 | 0.5431 |
| External test set (eICU) | SpO2 | ETS | 20 | 1.8263 | 0.9366 | 0.5907 |
| External test set (eICU) | SpO2 | ETS | 25 | 1.9212 | 0.9993 | 0.6301 |
| External test set (eICU) | SpO2 | ETS | 30 | 2.0034 | 1.0530 | 0.6639 |
| External test set (eICU) | SpO2 | ETS | 35 | 2.0755 | 1.1010 | 0.6941 |
| External test set (eICU) | SpO2 | ETS | 40 | 2.1409 | 1.1437 | 0.7210 |
| External test set (eICU) | SpO2 | ETS | 45 | 2.1993 | 1.1826 | 0.7454 |
| External test set (eICU) | SpO2 | ETS | 50 | 2.2556 | 1.2179 | 0.7676 |
| External test set (eICU) | SpO2 | ETS | 55 | 2.3043 | 1.2499 | 0.7876 |
| External test set (eICU) | SpO2 | ETS | 60 | 2.3504 | 1.2793 | 0.8062 |
| External test set (eICU) | SpO2 | ETS | 65 | 2.3936 | 1.3067 | 0.8234 |
| External test set (eICU) | SpO2 | ETS | 70 | 2.4345 | 1.3319 | 0.8391 |
| External test set (eICU) | SpO2 | ETS | 75 | 2.4701 | 1.3553 | 0.8539 |
| External test set (eICU) | SpO2 | ETS | 80 | 2.5050 | 1.3773 | 0.8677 |
| External test set (eICU) | SpO2 | ETS | 85 | 2.5403 | 1.3978 | 0.8805 |
| External test set (eICU) | SpO2 | ETS | 90 | 2.5738 | 1.4171 | 0.8928 |
| External test set (eICU) | SpO2 | ETS | 95 | 2.6054 | 1.4353 | 0.9042 |
| External test set (eICU) | SpO2 | ETS | 100 | 2.6372 | 1.4523 | 0.9149 |
| External test set (eICU) | SpO2 | ETS | 105 | 2.6681 | 1.4687 | 0.9252 |
| External test set (eICU) | SpO2 | ETS | 110 | 2.6982 | 1.4850 | 0.9355 |
| External test set (eICU) | SpO2 | ETS | 115 | 2.7245 | 1.4997 | 0.9448 |
| External test set (eICU) | SpO2 | ETS | 120 | 2.7519 | 1.5142 | 0.9539 |
| External test set (eICU) | SpO2 | GRU | 5 | 1.2644 | 0.7073 | 0.4458 |
| External test set (eICU) | SpO2 | GRU | 10 | 1.4421 | 0.8494 | 0.5358 |
| External test set (eICU) | SpO2 | GRU | 15 | 1.5409 | 0.9345 | 0.5896 |
| External test set (eICU) | SpO2 | GRU | 20 | 1.6127 | 1.0007 | 0.6315 |
| External test set (eICU) | SpO2 | GRU | 25 | 1.6708 | 1.0565 | 0.6668 |
| External test set (eICU) | SpO2 | GRU | 30 | 1.7182 | 1.1001 | 0.6941 |
| External test set (eICU) | SpO2 | GRU | 35 | 1.7585 | 1.1390 | 0.7188 |
| External test set (eICU) | SpO2 | GRU | 40 | 1.7927 | 1.1706 | 0.7385 |
| External test set (eICU) | SpO2 | GRU | 45 | 1.8241 | 1.2016 | 0.7581 |
| External test set (eICU) | SpO2 | GRU | 50 | 1.8519 | 1.2314 | 0.7769 |
| External test set (eICU) | SpO2 | GRU | 55 | 1.8771 | 1.2565 | 0.7926 |
| External test set (eICU) | SpO2 | GRU | 60 | 1.9000 | 1.2787 | 0.8066 |
| External test set (eICU) | SpO2 | GRU | 65 | 1.9217 | 1.3025 | 0.8217 |
| External test set (eICU) | SpO2 | GRU | 70 | 1.9406 | 1.3219 | 0.8337 |
| External test set (eICU) | SpO2 | GRU | 75 | 1.9590 | 1.3417 | 0.8463 |
| External test set (eICU) | SpO2 | GRU | 80 | 1.9761 | 1.3593 | 0.8575 |
| External test set (eICU) | SpO2 | GRU | 85 | 1.9911 | 1.3746 | 0.8670 |
| External test set (eICU) | SpO2 | GRU | 90 | 2.0069 | 1.3919 | 0.8780 |
| External test set (eICU) | SpO2 | GRU | 95 | 2.0205 | 1.4037 | 0.8854 |
| External test set (eICU) | SpO2 | GRU | 100 | 2.0329 | 1.4163 | 0.8934 |
| External test set (eICU) | SpO2 | GRU | 105 | 2.0439 | 1.4261 | 0.8996 |
| External test set (eICU) | SpO2 | GRU | 110 | 2.0562 | 1.4406 | 0.9088 |
| External test set (eICU) | SpO2 | GRU | 115 | 2.0667 | 1.4512 | 0.9155 |
| External test set (eICU) | SpO2 | GRU | 120 | 2.0749 | 1.4591 | 0.9204 |
| External test set (eICU) | SpO2 | Naive | 5 | 1.3634 | 0.5656 | 0.3567 |
| External test set (eICU) | SpO2 | Naive | 10 | 1.6198 | 0.7428 | 0.4685 |
| External test set (eICU) | SpO2 | Naive | 15 | 1.7600 | 0.8484 | 0.5352 |
| External test set (eICU) | SpO2 | Naive | 20 | 1.8566 | 0.9273 | 0.5849 |
| External test set (eICU) | SpO2 | Naive | 25 | 1.9379 | 0.9919 | 0.6256 |
| External test set (eICU) | SpO2 | Naive | 30 | 2.0037 | 1.0459 | 0.6595 |
| External test set (eICU) | SpO2 | Naive | 35 | 2.0604 | 1.0939 | 0.6897 |
| External test set (eICU) | SpO2 | Naive | 40 | 2.1075 | 1.1357 | 0.7159 |
| External test set (eICU) | SpO2 | Naive | 45 | 2.1514 | 1.1743 | 0.7402 |
| External test set (eICU) | SpO2 | Naive | 50 | 2.1898 | 1.2084 | 0.7616 |
| External test set (eICU) | SpO2 | Naive | 55 | 2.2257 | 1.2390 | 0.7808 |
| External test set (eICU) | SpO2 | Naive | 60 | 2.2589 | 1.2679 | 0.7989 |
| External test set (eICU) | SpO2 | Naive | 65 | 2.2860 | 1.2941 | 0.8154 |
| External test set (eICU) | SpO2 | Naive | 70 | 2.3101 | 1.3174 | 0.8300 |
| External test set (eICU) | SpO2 | Naive | 75 | 2.3324 | 1.3394 | 0.8438 |
| External test set (eICU) | SpO2 | Naive | 80 | 2.3540 | 1.3602 | 0.8569 |
| External test set (eICU) | SpO2 | Naive | 85 | 2.3735 | 1.3791 | 0.8688 |
| External test set (eICU) | SpO2 | Naive | 90 | 2.3920 | 1.3967 | 0.8799 |
| External test set (eICU) | SpO2 | Naive | 95 | 2.4098 | 1.4133 | 0.8904 |
| External test set (eICU) | SpO2 | Naive | 100 | 2.4264 | 1.4287 | 0.9000 |
| External test set (eICU) | SpO2 | Naive | 105 | 2.4432 | 1.4429 | 0.9090 |
| External test set (eICU) | SpO2 | Naive | 110 | 2.4575 | 1.4573 | 0.9181 |
| External test set (eICU) | SpO2 | Naive | 115 | 2.4716 | 1.4705 | 0.9264 |
| External test set (eICU) | SpO2 | Naive | 120 | 2.4846 | 1.4834 | 0.9345 |
| External test set (eICU) | SpO2 | Theta | 5 | 1.3165 | 0.6140 | 0.3873 |
| External test set (eICU) | SpO2 | Theta | 10 | 1.5438 | 0.7654 | 0.4828 |
| External test set (eICU) | SpO2 | Theta | 15 | 1.6782 | 0.8636 | 0.5447 |
| External test set (eICU) | SpO2 | Theta | 20 | 1.7805 | 0.9407 | 0.5933 |
| External test set (eICU) | SpO2 | Theta | 25 | 1.8656 | 1.0052 | 0.6339 |
| External test set (eICU) | SpO2 | Theta | 30 | 1.9365 | 1.0607 | 0.6688 |
| External test set (eICU) | SpO2 | Theta | 35 | 1.9994 | 1.1105 | 0.7002 |
| External test set (eICU) | SpO2 | Theta | 40 | 2.0555 | 1.1551 | 0.7282 |
| External test set (eICU) | SpO2 | Theta | 45 | 2.1064 | 1.1961 | 0.7540 |
| External test set (eICU) | SpO2 | Theta | 50 | 2.1535 | 1.2336 | 0.7775 |
| External test set (eICU) | SpO2 | Theta | 55 | 2.1965 | 1.2679 | 0.7991 |
| External test set (eICU) | SpO2 | Theta | 60 | 2.2369 | 1.2999 | 0.8193 |
| External test set (eICU) | SpO2 | Theta | 65 | 2.2726 | 1.3298 | 0.8381 |
| External test set (eICU) | SpO2 | Theta | 70 | 2.3069 | 1.3577 | 0.8556 |
| External test set (eICU) | SpO2 | Theta | 75 | 2.3385 | 1.3841 | 0.8722 |
| External test set (eICU) | SpO2 | Theta | 80 | 2.3700 | 1.4093 | 0.8881 |
| External test set (eICU) | SpO2 | Theta | 85 | 2.4003 | 1.4330 | 0.9030 |
| External test set (eICU) | SpO2 | Theta | 90 | 2.4289 | 1.4554 | 0.9171 |
| External test set (eICU) | SpO2 | Theta | 95 | 2.4569 | 1.4768 | 0.9306 |
| External test set (eICU) | SpO2 | Theta | 100 | 2.4840 | 1.4971 | 0.9434 |
| External test set (eICU) | SpO2 | Theta | 105 | 2.5115 | 1.5170 | 0.9559 |
| External test set (eICU) | SpO2 | Theta | 110 | 2.5373 | 1.5368 | 0.9684 |
| External test set (eICU) | SpO2 | Theta | 115 | 2.5619 | 1.5550 | 0.9799 |
| External test set (eICU) | SpO2 | Theta | 120 | 2.5868 | 1.5731 | 0.9913 |
| External test set (eICU) | SpO2 | Transformer | 5 | 1.2787 | 0.7075 | 0.4460 |
| External test set (eICU) | SpO2 | Transformer | 10 | 1.4567 | 0.8405 | 0.5298 |
| External test set (eICU) | SpO2 | Transformer | 15 | 1.5547 | 0.9226 | 0.5816 |
| External test set (eICU) | SpO2 | Transformer | 20 | 1.6253 | 0.9865 | 0.6219 |
| External test set (eICU) | SpO2 | Transformer | 25 | 1.6818 | 1.0377 | 0.6541 |
| External test set (eICU) | SpO2 | Transformer | 30 | 1.7286 | 1.0810 | 0.6814 |
| External test set (eICU) | SpO2 | Transformer | 35 | 1.7673 | 1.1173 | 0.7043 |
| External test set (eICU) | SpO2 | Transformer | 40 | 1.8012 | 1.1501 | 0.7249 |
| External test set (eICU) | SpO2 | Transformer | 45 | 1.8319 | 1.1791 | 0.7432 |
| External test set (eICU) | SpO2 | Transformer | 50 | 1.8588 | 1.2052 | 0.7596 |
| External test set (eICU) | SpO2 | Transformer | 55 | 1.8835 | 1.2296 | 0.7750 |
| External test set (eICU) | SpO2 | Transformer | 60 | 1.9055 | 1.2513 | 0.7886 |
| External test set (eICU) | SpO2 | Transformer | 65 | 1.9259 | 1.2717 | 0.8015 |
| External test set (eICU) | SpO2 | Transformer | 70 | 1.9443 | 1.2904 | 0.8132 |
| External test set (eICU) | SpO2 | Transformer | 75 | 1.9619 | 1.3077 | 0.8241 |
| External test set (eICU) | SpO2 | Transformer | 80 | 1.9785 | 1.3241 | 0.8345 |
| External test set (eICU) | SpO2 | Transformer | 85 | 1.9935 | 1.3391 | 0.8440 |
| External test set (eICU) | SpO2 | Transformer | 90 | 2.0079 | 1.3531 | 0.8529 |
| External test set (eICU) | SpO2 | Transformer | 95 | 2.0219 | 1.3670 | 0.8617 |
| External test set (eICU) | SpO2 | Transformer | 100 | 2.0341 | 1.3800 | 0.8699 |
| External test set (eICU) | SpO2 | Transformer | 105 | 2.0455 | 1.3918 | 0.8773 |
| External test set (eICU) | SpO2 | Transformer | 110 | 2.0565 | 1.4036 | 0.8849 |
| External test set (eICU) | SpO2 | Transformer | 115 | 2.0669 | 1.4144 | 0.8917 |
| External test set (eICU) | SpO2 | Transformer | 120 | 2.0762 | 1.4248 | 0.8983 |
| External test set (eICU) | Systolic BP | AR NNet | 5 | 9.4987 | 6.1602 | 3.7598 |
| External test set (eICU) | Systolic BP | AR NNet | 10 | 10.8342 | 7.2403 | 4.4156 |
| External test set (eICU) | Systolic BP | AR NNet | 15 | 11.5766 | 7.8851 | 4.8080 |
| External test set (eICU) | Systolic BP | AR NNet | 20 | 12.1333 | 8.3784 | 5.1088 |
| External test set (eICU) | Systolic BP | AR NNet | 25 | 12.5596 | 8.7685 | 5.3469 |
| External test set (eICU) | Systolic BP | AR NNet | 30 | 12.9255 | 9.0986 | 5.5483 |
| External test set (eICU) | Systolic BP | AR NNet | 35 | 13.2643 | 9.4028 | 5.7339 |
| External test set (eICU) | Systolic BP | AR NNet | 40 | 13.5257 | 9.6392 | 5.8780 |
| External test set (eICU) | Systolic BP | AR NNet | 45 | 13.7467 | 9.8420 | 6.0020 |
| External test set (eICU) | Systolic BP | AR NNet | 50 | 13.9677 | 10.0370 | 6.1211 |
| External test set (eICU) | Systolic BP | AR NNet | 55 | 14.1515 | 10.2034 | 6.2229 |
| External test set (eICU) | Systolic BP | AR NNet | 60 | 14.3325 | 10.3624 | 6.3199 |
| External test set (eICU) | Systolic BP | AR NNet | 65 | 14.5072 | 10.5188 | 6.4159 |
| External test set (eICU) | Systolic BP | AR NNet | 70 | 14.6529 | 10.6455 | 6.4929 |
| External test set (eICU) | Systolic BP | AR NNet | 75 | 14.7777 | 10.7586 | 6.5621 |
| External test set (eICU) | Systolic BP | AR NNet | 80 | 14.9088 | 10.8734 | 6.6325 |
| External test set (eICU) | Systolic BP | AR NNet | 85 | 15.0196 | 10.9755 | 6.6950 |
| External test set (eICU) | Systolic BP | AR NNet | 90 | 15.1359 | 11.0771 | 6.7570 |
| External test set (eICU) | Systolic BP | AR NNet | 95 | 15.2505 | 11.1770 | 6.8181 |
| External test set (eICU) | Systolic BP | AR NNet | 100 | 15.3499 | 11.2655 | 6.8721 |
| External test set (eICU) | Systolic BP | AR NNet | 105 | 15.4384 | 11.3466 | 6.9216 |
| External test set (eICU) | Systolic BP | AR NNet | 110 | 15.5437 | 11.4346 | 6.9753 |
| External test set (eICU) | Systolic BP | AR NNet | 115 | 15.6357 | 11.5156 | 7.0249 |
| External test set (eICU) | Systolic BP | AR NNet | 120 | 15.7365 | 11.6005 | 7.0768 |
| External test set (eICU) | Systolic BP | ARIMA | 5 | 9.1563 | 5.4973 | 3.3529 |
| External test set (eICU) | Systolic BP | ARIMA | 10 | 10.9253 | 7.0339 | 4.2889 |
| External test set (eICU) | Systolic BP | ARIMA | 15 | 11.8103 | 7.8365 | 4.7780 |
| External test set (eICU) | Systolic BP | ARIMA | 20 | 12.4773 | 8.4354 | 5.1437 |
| External test set (eICU) | Systolic BP | ARIMA | 25 | 13.0027 | 8.9051 | 5.4302 |
| External test set (eICU) | Systolic BP | ARIMA | 30 | 13.4529 | 9.2945 | 5.6682 |
| External test set (eICU) | Systolic BP | ARIMA | 35 | 13.8859 | 9.6566 | 5.8892 |
| External test set (eICU) | Systolic BP | ARIMA | 40 | 14.2262 | 9.9480 | 6.0672 |
| External test set (eICU) | Systolic BP | ARIMA | 45 | 14.5207 | 10.1957 | 6.2186 |
| External test set (eICU) | Systolic BP | ARIMA | 50 | 14.8234 | 10.4377 | 6.3666 |
| External test set (eICU) | Systolic BP | ARIMA | 55 | 15.0828 | 10.6517 | 6.4976 |
| External test set (eICU) | Systolic BP | ARIMA | 60 | 15.3350 | 10.8482 | 6.6180 |
| External test set (eICU) | Systolic BP | ARIMA | 65 | 15.6056 | 11.0575 | 6.7461 |
| External test set (eICU) | Systolic BP | ARIMA | 70 | 15.8184 | 11.2200 | 6.8454 |
| External test set (eICU) | Systolic BP | ARIMA | 75 | 16.0100 | 11.3631 | 6.9328 |
| External test set (eICU) | Systolic BP | ARIMA | 80 | 16.2030 | 11.5091 | 7.0224 |
| External test set (eICU) | Systolic BP | ARIMA | 85 | 16.3826 | 11.6404 | 7.1029 |
| External test set (eICU) | Systolic BP | ARIMA | 90 | 16.5573 | 11.7673 | 7.1808 |
| External test set (eICU) | Systolic BP | ARIMA | 95 | 16.7524 | 11.9011 | 7.2622 |
| External test set (eICU) | Systolic BP | ARIMA | 100 | 16.9149 | 12.0143 | 7.3314 |
| External test set (eICU) | Systolic BP | ARIMA | 105 | 17.0667 | 12.1160 | 7.3933 |
| External test set (eICU) | Systolic BP | ARIMA | 110 | 17.2367 | 12.2285 | 7.4618 |
| External test set (eICU) | Systolic BP | ARIMA | 115 | 17.3922 | 12.3324 | 7.5254 |
| External test set (eICU) | Systolic BP | ARIMA | 120 | 17.5510 | 12.4373 | 7.5896 |
| External test set (eICU) | Systolic BP | ETS | 5 | 9.3164 | 5.4316 | 3.3145 |
| External test set (eICU) | Systolic BP | ETS | 10 | 11.1678 | 6.9108 | 4.2158 |
| External test set (eICU) | Systolic BP | ETS | 15 | 12.1797 | 7.7662 | 4.7364 |
| External test set (eICU) | Systolic BP | ETS | 20 | 13.0014 | 8.4595 | 5.1593 |
| External test set (eICU) | Systolic BP | ETS | 25 | 13.6392 | 9.0087 | 5.4943 |
| External test set (eICU) | Systolic BP | ETS | 30 | 14.1774 | 9.4678 | 5.7742 |
| External test set (eICU) | Systolic BP | ETS | 35 | 14.7376 | 9.9257 | 6.0536 |
| External test set (eICU) | Systolic BP | ETS | 40 | 15.1285 | 10.2595 | 6.2570 |
| External test set (eICU) | Systolic BP | ETS | 45 | 15.4152 | 10.5066 | 6.4067 |
| External test set (eICU) | Systolic BP | ETS | 50 | 15.7779 | 10.7985 | 6.5849 |
| External test set (eICU) | Systolic BP | ETS | 55 | 16.0891 | 11.0488 | 6.7376 |
| External test set (eICU) | Systolic BP | ETS | 60 | 16.3734 | 11.2704 | 6.8729 |
| External test set (eICU) | Systolic BP | ETS | 65 | 16.7419 | 11.5653 | 7.0545 |
| External test set (eICU) | Systolic BP | ETS | 70 | 16.9724 | 11.7445 | 7.1634 |
| External test set (eICU) | Systolic BP | ETS | 75 | 17.1747 | 11.8937 | 7.2538 |
| External test set (eICU) | Systolic BP | ETS | 80 | 17.3848 | 12.0606 | 7.3558 |
| External test set (eICU) | Systolic BP | ETS | 85 | 17.5783 | 12.2071 | 7.4456 |
| External test set (eICU) | Systolic BP | ETS | 90 | 17.7581 | 12.3379 | 7.5259 |
| External test set (eICU) | Systolic BP | ETS | 95 | 17.9655 | 12.4936 | 7.6216 |
| External test set (eICU) | Systolic BP | ETS | 100 | 18.1163 | 12.6038 | 7.6885 |
| External test set (eICU) | Systolic BP | ETS | 105 | 18.2271 | 12.6724 | 7.7293 |
| External test set (eICU) | Systolic BP | ETS | 110 | 18.3848 | 12.7830 | 7.7967 |
| External test set (eICU) | Systolic BP | ETS | 115 | 18.5490 | 12.8872 | 7.8604 |
| External test set (eICU) | Systolic BP | ETS | 120 | 18.6936 | 12.9880 | 7.9220 |
| External test set (eICU) | Systolic BP | GRU | 5 | 8.7538 | 5.2866 | 3.2341 |
| External test set (eICU) | Systolic BP | GRU | 10 | 10.3333 | 6.6894 | 4.0906 |
| External test set (eICU) | Systolic BP | GRU | 15 | 11.1160 | 7.4489 | 4.5554 |
| External test set (eICU) | Systolic BP | GRU | 20 | 11.7302 | 8.0377 | 4.9169 |
| External test set (eICU) | Systolic BP | GRU | 25 | 12.2129 | 8.5063 | 5.2045 |
| External test set (eICU) | Systolic BP | GRU | 30 | 12.6212 | 8.9001 | 5.4468 |
| External test set (eICU) | Systolic BP | GRU | 35 | 12.9659 | 9.2238 | 5.6447 |
| External test set (eICU) | Systolic BP | GRU | 40 | 13.2277 | 9.4813 | 5.8033 |
| External test set (eICU) | Systolic BP | GRU | 45 | 13.4302 | 9.6807 | 5.9248 |
| External test set (eICU) | Systolic BP | GRU | 50 | 13.6582 | 9.8900 | 6.0536 |
| External test set (eICU) | Systolic BP | GRU | 55 | 13.8575 | 10.0810 | 6.1714 |
| External test set (eICU) | Systolic BP | GRU | 60 | 14.0359 | 10.2440 | 6.2710 |
| External test set (eICU) | Systolic BP | GRU | 65 | 14.2237 | 10.4161 | 6.3785 |
| External test set (eICU) | Systolic BP | GRU | 70 | 14.3526 | 10.5349 | 6.4502 |
| External test set (eICU) | Systolic BP | GRU | 75 | 14.4749 | 10.6526 | 6.5229 |
| External test set (eICU) | Systolic BP | GRU | 80 | 14.5930 | 10.7591 | 6.5875 |
| External test set (eICU) | Systolic BP | GRU | 85 | 14.7048 | 10.8670 | 6.6544 |
| External test set (eICU) | Systolic BP | GRU | 90 | 14.7973 | 10.9562 | 6.7093 |
| External test set (eICU) | Systolic BP | GRU | 95 | 14.9007 | 11.0505 | 6.7666 |
| External test set (eICU) | Systolic BP | GRU | 100 | 14.9869 | 11.1325 | 6.8168 |
| External test set (eICU) | Systolic BP | GRU | 105 | 15.0461 | 11.1898 | 6.8513 |
| External test set (eICU) | Systolic BP | GRU | 110 | 15.1381 | 11.2766 | 6.9052 |
| External test set (eICU) | Systolic BP | GRU | 115 | 15.2217 | 11.3540 | 6.9529 |
| External test set (eICU) | Systolic BP | GRU | 120 | 15.2936 | 11.4181 | 6.9917 |
| External test set (eICU) | Systolic BP | Naive | 5 | 9.4323 | 5.3063 | 3.2409 |
| External test set (eICU) | Systolic BP | Naive | 10 | 11.5652 | 7.0124 | 4.2783 |
| External test set (eICU) | Systolic BP | Naive | 15 | 12.5117 | 7.8826 | 4.8064 |
| External test set (eICU) | Systolic BP | Naive | 20 | 13.2776 | 8.5843 | 5.2338 |
| External test set (eICU) | Systolic BP | Naive | 25 | 13.8653 | 9.1358 | 5.5700 |
| External test set (eICU) | Systolic BP | Naive | 30 | 14.3434 | 9.5899 | 5.8469 |
| External test set (eICU) | Systolic BP | Naive | 35 | 14.8785 | 10.0628 | 6.1352 |
| External test set (eICU) | Systolic BP | Naive | 40 | 15.2260 | 10.3990 | 6.3403 |
| External test set (eICU) | Systolic BP | Naive | 45 | 15.4087 | 10.6011 | 6.4612 |
| External test set (eICU) | Systolic BP | Naive | 50 | 15.6993 | 10.8772 | 6.6300 |
| External test set (eICU) | Systolic BP | Naive | 55 | 15.9528 | 11.1159 | 6.7755 |
| External test set (eICU) | Systolic BP | Naive | 60 | 16.1744 | 11.3260 | 6.9038 |
| External test set (eICU) | Systolic BP | Naive | 65 | 16.5098 | 11.6200 | 7.0854 |
| External test set (eICU) | Systolic BP | Naive | 70 | 16.6892 | 11.7927 | 7.1906 |
| External test set (eICU) | Systolic BP | Naive | 75 | 16.8044 | 11.9070 | 7.2587 |
| External test set (eICU) | Systolic BP | Naive | 80 | 16.9539 | 12.0572 | 7.3506 |
| External test set (eICU) | Systolic BP | Naive | 85 | 17.0843 | 12.1867 | 7.4297 |
| External test set (eICU) | Systolic BP | Naive | 90 | 17.2044 | 12.3022 | 7.5007 |
| External test set (eICU) | Systolic BP | Naive | 95 | 17.3793 | 12.4576 | 7.5961 |
| External test set (eICU) | Systolic BP | Naive | 100 | 17.4799 | 12.5620 | 7.6602 |
| External test set (eICU) | Systolic BP | Naive | 105 | 17.4848 | 12.5850 | 7.6721 |
| External test set (eICU) | Systolic BP | Naive | 110 | 17.5788 | 12.6803 | 7.7298 |
| External test set (eICU) | Systolic BP | Naive | 115 | 17.6748 | 12.7645 | 7.7809 |
| External test set (eICU) | Systolic BP | Naive | 120 | 17.7711 | 12.8530 | 7.8351 |
| External test set (eICU) | Systolic BP | Theta | 5 | 9.3021 | 5.4252 | 3.3108 |
| External test set (eICU) | Systolic BP | Theta | 10 | 11.1816 | 6.9315 | 4.2288 |
| External test set (eICU) | Systolic BP | Theta | 15 | 12.2032 | 7.8107 | 4.7642 |
| External test set (eICU) | Systolic BP | Theta | 20 | 13.0343 | 8.5296 | 5.2031 |
| External test set (eICU) | Systolic BP | Theta | 25 | 13.6895 | 9.1081 | 5.5563 |
| External test set (eICU) | Systolic BP | Theta | 30 | 14.2525 | 9.5998 | 5.8565 |
| External test set (eICU) | Systolic BP | Theta | 35 | 14.8444 | 10.0961 | 6.1596 |
| External test set (eICU) | Systolic BP | Theta | 40 | 15.2691 | 10.4696 | 6.3876 |
| External test set (eICU) | Systolic BP | Theta | 45 | 15.6086 | 10.7622 | 6.5653 |
| External test set (eICU) | Systolic BP | Theta | 50 | 16.0195 | 11.1025 | 6.7736 |
| External test set (eICU) | Systolic BP | Theta | 55 | 16.3804 | 11.3995 | 6.9553 |
| External test set (eICU) | Systolic BP | Theta | 60 | 16.7192 | 11.6704 | 7.1211 |
| External test set (eICU) | Systolic BP | Theta | 65 | 17.1653 | 12.0232 | 7.3383 |
| External test set (eICU) | Systolic BP | Theta | 70 | 17.4626 | 12.2565 | 7.4807 |
| External test set (eICU) | Systolic BP | Theta | 75 | 17.7442 | 12.4667 | 7.6087 |
| External test set (eICU) | Systolic BP | Theta | 80 | 18.0408 | 12.6938 | 7.7478 |
| External test set (eICU) | Systolic BP | Theta | 85 | 18.3110 | 12.8988 | 7.8738 |
| External test set (eICU) | Systolic BP | Theta | 90 | 18.5749 | 13.0925 | 7.9928 |
| External test set (eICU) | Systolic BP | Theta | 95 | 18.8841 | 13.3174 | 8.1307 |
| External test set (eICU) | Systolic BP | Theta | 100 | 19.1256 | 13.4914 | 8.2374 |
| External test set (eICU) | Systolic BP | Theta | 105 | 19.3444 | 13.6306 | 8.3219 |
| External test set (eICU) | Systolic BP | Theta | 110 | 19.6110 | 13.8154 | 8.4352 |
| External test set (eICU) | Systolic BP | Theta | 115 | 19.8671 | 13.9879 | 8.5411 |
| External test set (eICU) | Systolic BP | Theta | 120 | 20.1280 | 14.1632 | 8.6485 |
| External test set (eICU) | Systolic BP | Transformer | 5 | 8.9297 | 5.4600 | 3.3376 |
| External test set (eICU) | Systolic BP | Transformer | 10 | 10.5501 | 6.8765 | 4.2016 |
| External test set (eICU) | Systolic BP | Transformer | 15 | 11.2839 | 7.5980 | 4.6418 |
| External test set (eICU) | Systolic BP | Transformer | 20 | 11.8817 | 8.1702 | 4.9925 |
| External test set (eICU) | Systolic BP | Transformer | 25 | 12.3444 | 8.6160 | 5.2654 |
| External test set (eICU) | Systolic BP | Transformer | 30 | 12.7340 | 8.9882 | 5.4934 |
| External test set (eICU) | Systolic BP | Transformer | 35 | 13.0989 | 9.3264 | 5.7009 |
| External test set (eICU) | Systolic BP | Transformer | 40 | 13.3473 | 9.5707 | 5.8506 |
| External test set (eICU) | Systolic BP | Transformer | 45 | 13.5157 | 9.7453 | 5.9568 |
| External test set (eICU) | Systolic BP | Transformer | 50 | 13.7350 | 9.9495 | 6.0824 |
| External test set (eICU) | Systolic BP | Transformer | 55 | 13.9190 | 10.1228 | 6.1889 |
| External test set (eICU) | Systolic BP | Transformer | 60 | 14.0846 | 10.2781 | 6.2844 |
| External test set (eICU) | Systolic BP | Transformer | 65 | 14.2885 | 10.4586 | 6.3968 |
| External test set (eICU) | Systolic BP | Transformer | 70 | 14.4125 | 10.5765 | 6.4691 |
| External test set (eICU) | Systolic BP | Transformer | 75 | 14.5151 | 10.6749 | 6.5293 |
| External test set (eICU) | Systolic BP | Transformer | 80 | 14.6314 | 10.7838 | 6.5962 |
| External test set (eICU) | Systolic BP | Transformer | 85 | 14.7313 | 10.8779 | 6.6542 |
| External test set (eICU) | Systolic BP | Transformer | 90 | 14.8268 | 10.9689 | 6.7102 |
| External test set (eICU) | Systolic BP | Transformer | 95 | 14.9320 | 11.0660 | 6.7704 |
| External test set (eICU) | Systolic BP | Transformer | 100 | 15.0135 | 11.1424 | 6.8171 |
| External test set (eICU) | Systolic BP | Transformer | 105 | 15.0639 | 11.1923 | 6.8469 |
| External test set (eICU) | Systolic BP | Transformer | 110 | 15.1464 | 11.2666 | 6.8930 |
| External test set (eICU) | Systolic BP | Transformer | 115 | 15.2174 | 11.3340 | 6.9349 |
| External test set (eICU) | Systolic BP | Transformer | 120 | 15.2927 | 11.4023 | 6.9772 |
| Internal test set | Central venous pressure | AR NNet | 5 | 2.6645 | 1.3197 | 0.7901 |
| Internal test set | Central venous pressure | AR NNet | 10 | 3.0884 | 1.5951 | 0.9550 |
| Internal test set | Central venous pressure | AR NNet | 15 | 3.3221 | 1.7711 | 1.0605 |
| Internal test set | Central venous pressure | AR NNet | 20 | 3.4778 | 1.8997 | 1.1377 |
| Internal test set | Central venous pressure | AR NNet | 25 | 3.5953 | 2.0012 | 1.1986 |
| Internal test set | Central venous pressure | AR NNet | 30 | 3.6810 | 2.0834 | 1.2477 |
| Internal test set | Central venous pressure | AR NNet | 35 | 3.7554 | 2.1584 | 1.2928 |
| Internal test set | Central venous pressure | AR NNet | 40 | 3.8181 | 2.2209 | 1.3302 |
| Internal test set | Central venous pressure | AR NNet | 45 | 3.8729 | 2.2762 | 1.3633 |
| Internal test set | Central venous pressure | AR NNet | 50 | 3.9251 | 2.3265 | 1.3935 |
| Internal test set | Central venous pressure | AR NNet | 55 | 3.9652 | 2.3705 | 1.4199 |
| Internal test set | Central venous pressure | AR NNet | 60 | 4.0058 | 2.4112 | 1.4443 |
| Internal test set | Central venous pressure | AR NNet | 65 | 4.0423 | 2.4499 | 1.4673 |
| Internal test set | Central venous pressure | AR NNet | 70 | 4.0961 | 2.4888 | 1.4907 |
| Internal test set | Central venous pressure | AR NNet | 75 | 4.1437 | 2.5253 | 1.5127 |
| Internal test set | Central venous pressure | AR NNet | 80 | 4.1796 | 2.5586 | 1.5325 |
| Internal test set | Central venous pressure | AR NNet | 85 | 4.2123 | 2.5907 | 1.5516 |
| Internal test set | Central venous pressure | AR NNet | 90 | 4.2397 | 2.6201 | 1.5690 |
| Internal test set | Central venous pressure | AR NNet | 95 | 4.2628 | 2.6483 | 1.5856 |
| Internal test set | Central venous pressure | AR NNet | 100 | 4.2744 | 2.6734 | 1.6004 |
| Internal test set | Central venous pressure | AR NNet | 105 | 4.3009 | 2.7000 | 1.6160 |
| Internal test set | Central venous pressure | AR NNet | 110 | 4.3275 | 2.7256 | 1.6311 |
| Internal test set | Central venous pressure | AR NNet | 115 | 4.3510 | 2.7504 | 1.6458 |
| Internal test set | Central venous pressure | AR NNet | 120 | 4.3735 | 2.7737 | 1.6596 |
| Internal test set | Central venous pressure | ARIMA | 5 | 2.5993 | 1.1334 | 0.6778 |
| Internal test set | Central venous pressure | ARIMA | 10 | 3.1076 | 1.4994 | 0.8973 |
| Internal test set | Central venous pressure | ARIMA | 15 | 3.3799 | 1.7175 | 1.0287 |
| Internal test set | Central venous pressure | ARIMA | 20 | 3.5736 | 1.8768 | 1.1243 |
| Internal test set | Central venous pressure | ARIMA | 25 | 3.7144 | 2.0015 | 1.1992 |
| Internal test set | Central venous pressure | ARIMA | 30 | 3.8360 | 2.1020 | 1.2594 |
| Internal test set | Central venous pressure | ARIMA | 35 | 3.9331 | 2.1917 | 1.3133 |
| Internal test set | Central venous pressure | ARIMA | 40 | 4.0147 | 2.2673 | 1.3588 |
| Internal test set | Central venous pressure | ARIMA | 45 | 4.0824 | 2.3345 | 1.3991 |
| Internal test set | Central venous pressure | ARIMA | 50 | 4.1365 | 2.3941 | 1.4347 |
| Internal test set | Central venous pressure | ARIMA | 55 | 4.1973 | 2.4514 | 1.4690 |
| Internal test set | Central venous pressure | ARIMA | 60 | 4.2476 | 2.4995 | 1.4978 |
| Internal test set | Central venous pressure | ARIMA | 65 | 4.2933 | 2.5468 | 1.5261 |
| Internal test set | Central venous pressure | ARIMA | 70 | 4.3390 | 2.5901 | 1.5522 |
| Internal test set | Central venous pressure | ARIMA | 75 | 4.3858 | 2.6328 | 1.5780 |
| Internal test set | Central venous pressure | ARIMA | 80 | 4.4313 | 2.6727 | 1.6020 |
| Internal test set | Central venous pressure | ARIMA | 85 | 4.4775 | 2.7132 | 1.6264 |
| Internal test set | Central venous pressure | ARIMA | 90 | 4.5209 | 2.7500 | 1.6484 |
| Internal test set | Central venous pressure | ARIMA | 95 | 4.5673 | 2.7873 | 1.6704 |
| Internal test set | Central venous pressure | ARIMA | 100 | 4.6060 | 2.8210 | 1.6903 |
| Internal test set | Central venous pressure | ARIMA | 105 | 4.6458 | 2.8533 | 1.7093 |
| Internal test set | Central venous pressure | ARIMA | 110 | 4.6796 | 2.8829 | 1.7269 |
| Internal test set | Central venous pressure | ARIMA | 115 | 4.7184 | 2.9138 | 1.7452 |
| Internal test set | Central venous pressure | ARIMA | 120 | 4.7520 | 2.9418 | 1.7620 |
| Internal test set | Central venous pressure | ETS | 5 | 2.8071 | 1.0830 | 0.6485 |
| Internal test set | Central venous pressure | ETS | 10 | 3.3367 | 1.4451 | 0.8655 |
| Internal test set | Central venous pressure | ETS | 15 | 3.6644 | 1.6844 | 1.0091 |
| Internal test set | Central venous pressure | ETS | 20 | 3.8984 | 1.8696 | 1.1203 |
| Internal test set | Central venous pressure | ETS | 25 | 4.0809 | 2.0159 | 1.2080 |
| Internal test set | Central venous pressure | ETS | 30 | 4.2334 | 2.1362 | 1.2803 |
| Internal test set | Central venous pressure | ETS | 35 | 4.3622 | 2.2437 | 1.3447 |
| Internal test set | Central venous pressure | ETS | 40 | 4.4742 | 2.3361 | 1.4000 |
| Internal test set | Central venous pressure | ETS | 45 | 4.5722 | 2.4151 | 1.4471 |
| Internal test set | Central venous pressure | ETS | 50 | 4.6621 | 2.4901 | 1.4919 |
| Internal test set | Central venous pressure | ETS | 55 | 4.7376 | 2.5560 | 1.5314 |
| Internal test set | Central venous pressure | ETS | 60 | 4.8009 | 2.6134 | 1.5659 |
| Internal test set | Central venous pressure | ETS | 65 | 4.8553 | 2.6663 | 1.5974 |
| Internal test set | Central venous pressure | ETS | 70 | 4.9057 | 2.7146 | 1.6264 |
| Internal test set | Central venous pressure | ETS | 75 | 4.9582 | 2.7606 | 1.6539 |
| Internal test set | Central venous pressure | ETS | 80 | 5.0051 | 2.8048 | 1.6804 |
| Internal test set | Central venous pressure | ETS | 85 | 5.0516 | 2.8475 | 1.7059 |
| Internal test set | Central venous pressure | ETS | 90 | 5.0950 | 2.8867 | 1.7293 |
| Internal test set | Central venous pressure | ETS | 95 | 5.1381 | 2.9249 | 1.7520 |
| Internal test set | Central venous pressure | ETS | 100 | 5.1804 | 2.9615 | 1.7736 |
| Internal test set | Central venous pressure | ETS | 105 | 5.2178 | 2.9950 | 1.7932 |
| Internal test set | Central venous pressure | ETS | 110 | 5.2584 | 3.0281 | 1.8129 |
| Internal test set | Central venous pressure | ETS | 115 | 5.2979 | 3.0599 | 1.8319 |
| Internal test set | Central venous pressure | ETS | 120 | 5.3381 | 3.0894 | 1.8493 |
| Internal test set | Central venous pressure | GRU | 5 | 2.1764 | 1.0374 | 0.6215 |
| Internal test set | Central venous pressure | GRU | 10 | 2.6929 | 1.3674 | 0.8196 |
| Internal test set | Central venous pressure | GRU | 15 | 2.9640 | 1.5701 | 0.9413 |
| Internal test set | Central venous pressure | GRU | 20 | 3.1358 | 1.7142 | 1.0278 |
| Internal test set | Central venous pressure | GRU | 25 | 3.2640 | 1.8274 | 1.0959 |
| Internal test set | Central venous pressure | GRU | 30 | 3.3673 | 1.9191 | 1.1512 |
| Internal test set | Central venous pressure | GRU | 35 | 3.4506 | 1.9933 | 1.1957 |
| Internal test set | Central venous pressure | GRU | 40 | 3.5190 | 2.0569 | 1.2337 |
| Internal test set | Central venous pressure | GRU | 45 | 3.5778 | 2.1120 | 1.2667 |
| Internal test set | Central venous pressure | GRU | 50 | 3.6336 | 2.1619 | 1.2965 |
| Internal test set | Central venous pressure | GRU | 55 | 3.6794 | 2.2058 | 1.3228 |
| Internal test set | Central venous pressure | GRU | 60 | 3.7217 | 2.2459 | 1.3467 |
| Internal test set | Central venous pressure | GRU | 65 | 3.7591 | 2.2832 | 1.3690 |
| Internal test set | Central venous pressure | GRU | 70 | 3.7926 | 2.3165 | 1.3888 |
| Internal test set | Central venous pressure | GRU | 75 | 3.8270 | 2.3472 | 1.4072 |
| Internal test set | Central venous pressure | GRU | 80 | 3.8581 | 2.3784 | 1.4259 |
| Internal test set | Central venous pressure | GRU | 85 | 3.8875 | 2.4077 | 1.4434 |
| Internal test set | Central venous pressure | GRU | 90 | 3.9151 | 2.4329 | 1.4584 |
| Internal test set | Central venous pressure | GRU | 95 | 3.9389 | 2.4582 | 1.4734 |
| Internal test set | Central venous pressure | GRU | 100 | 3.9639 | 2.4833 | 1.4881 |
| Internal test set | Central venous pressure | GRU | 105 | 3.9874 | 2.5068 | 1.5020 |
| Internal test set | Central venous pressure | GRU | 110 | 4.0107 | 2.5294 | 1.5154 |
| Internal test set | Central venous pressure | GRU | 115 | 4.0318 | 2.5502 | 1.5275 |
| Internal test set | Central venous pressure | GRU | 120 | 4.0542 | 2.5713 | 1.5400 |
| Internal test set | Central venous pressure | Naive | 5 | 2.2777 | 0.9420 | 0.5629 |
| Internal test set | Central venous pressure | Naive | 10 | 2.9561 | 1.3421 | 0.8025 |
| Internal test set | Central venous pressure | Naive | 15 | 3.3388 | 1.5926 | 0.9529 |
| Internal test set | Central venous pressure | Naive | 20 | 3.5854 | 1.7838 | 1.0677 |
| Internal test set | Central venous pressure | Naive | 25 | 3.7745 | 1.9321 | 1.1566 |
| Internal test set | Central venous pressure | Naive | 30 | 3.9243 | 2.0507 | 1.2278 |
| Internal test set | Central venous pressure | Naive | 35 | 4.0478 | 2.1574 | 1.2916 |
| Internal test set | Central venous pressure | Naive | 40 | 4.1508 | 2.2472 | 1.3454 |
| Internal test set | Central venous pressure | Naive | 45 | 4.2359 | 2.3215 | 1.3897 |
| Internal test set | Central venous pressure | Naive | 50 | 4.3152 | 2.3924 | 1.4320 |
| Internal test set | Central venous pressure | Naive | 55 | 4.3845 | 2.4561 | 1.4703 |
| Internal test set | Central venous pressure | Naive | 60 | 4.4370 | 2.5090 | 1.5021 |
| Internal test set | Central venous pressure | Naive | 65 | 4.4794 | 2.5586 | 1.5316 |
| Internal test set | Central venous pressure | Naive | 70 | 4.5170 | 2.6032 | 1.5583 |
| Internal test set | Central venous pressure | Naive | 75 | 4.5601 | 2.6453 | 1.5834 |
| Internal test set | Central venous pressure | Naive | 80 | 4.5985 | 2.6865 | 1.6082 |
| Internal test set | Central venous pressure | Naive | 85 | 4.6334 | 2.7255 | 1.6316 |
| Internal test set | Central venous pressure | Naive | 90 | 4.6674 | 2.7604 | 1.6524 |
| Internal test set | Central venous pressure | Naive | 95 | 4.7016 | 2.7965 | 1.6739 |
| Internal test set | Central venous pressure | Naive | 100 | 4.7323 | 2.8296 | 1.6933 |
| Internal test set | Central venous pressure | Naive | 105 | 4.7599 | 2.8585 | 1.7102 |
| Internal test set | Central venous pressure | Naive | 110 | 4.7858 | 2.8871 | 1.7272 |
| Internal test set | Central venous pressure | Naive | 115 | 4.8124 | 2.9155 | 1.7442 |
| Internal test set | Central venous pressure | Naive | 120 | 4.8384 | 2.9401 | 1.7588 |
| Internal test set | Central venous pressure | Theta | 5 | 3.5326 | 1.2045 | 0.7204 |
| Internal test set | Central venous pressure | Theta | 10 | 3.9848 | 1.5755 | 0.9425 |
| Internal test set | Central venous pressure | Theta | 15 | 4.2641 | 1.8202 | 1.0893 |
| Internal test set | Central venous pressure | Theta | 20 | 4.4658 | 2.0113 | 1.2041 |
| Internal test set | Central venous pressure | Theta | 25 | 4.6219 | 2.1643 | 1.2959 |
| Internal test set | Central venous pressure | Theta | 30 | 4.7581 | 2.2932 | 1.3733 |
| Internal test set | Central venous pressure | Theta | 35 | 4.8716 | 2.4087 | 1.4425 |
| Internal test set | Central venous pressure | Theta | 40 | 4.9770 | 2.5112 | 1.5039 |
| Internal test set | Central venous pressure | Theta | 45 | 5.0640 | 2.5996 | 1.5568 |
| Internal test set | Central venous pressure | Theta | 50 | 5.1541 | 2.6857 | 1.6083 |
| Internal test set | Central venous pressure | Theta | 55 | 5.2279 | 2.7626 | 1.6545 |
| Internal test set | Central venous pressure | Theta | 60 | 5.2948 | 2.8324 | 1.6965 |
| Internal test set | Central venous pressure | Theta | 65 | 5.3519 | 2.8974 | 1.7355 |
| Internal test set | Central venous pressure | Theta | 70 | 5.4119 | 2.9601 | 1.7731 |
| Internal test set | Central venous pressure | Theta | 75 | 5.4710 | 3.0196 | 1.8089 |
| Internal test set | Central venous pressure | Theta | 80 | 5.5342 | 3.0794 | 1.8449 |
| Internal test set | Central venous pressure | Theta | 85 | 5.5887 | 3.1365 | 1.8794 |
| Internal test set | Central venous pressure | Theta | 90 | 5.6469 | 3.1921 | 1.9127 |
| Internal test set | Central venous pressure | Theta | 95 | 5.7015 | 3.2454 | 1.9446 |
| Internal test set | Central venous pressure | Theta | 100 | 5.7587 | 3.2979 | 1.9759 |
| Internal test set | Central venous pressure | Theta | 105 | 5.8078 | 3.3467 | 2.0050 |
| Internal test set | Central venous pressure | Theta | 110 | 5.8639 | 3.3957 | 2.0344 |
| Internal test set | Central venous pressure | Theta | 115 | 5.9166 | 3.4427 | 2.0626 |
| Internal test set | Central venous pressure | Theta | 120 | 5.9727 | 3.4883 | 2.0898 |
| Internal test set | Central venous pressure | Transformer | 5 | 2.2098 | 1.1099 | 0.6658 |
| Internal test set | Central venous pressure | Transformer | 10 | 2.7299 | 1.4175 | 0.8501 |
| Internal test set | Central venous pressure | Transformer | 15 | 3.0014 | 1.6043 | 0.9622 |
| Internal test set | Central venous pressure | Transformer | 20 | 3.1713 | 1.7411 | 1.0443 |
| Internal test set | Central venous pressure | Transformer | 25 | 3.2984 | 1.8465 | 1.1075 |
| Internal test set | Central venous pressure | Transformer | 30 | 3.3994 | 1.9321 | 1.1590 |
| Internal test set | Central venous pressure | Transformer | 35 | 3.4810 | 2.0041 | 1.2021 |
| Internal test set | Central venous pressure | Transformer | 40 | 3.5485 | 2.0657 | 1.2390 |
| Internal test set | Central venous pressure | Transformer | 45 | 3.6059 | 2.1181 | 1.2703 |
| Internal test set | Central venous pressure | Transformer | 50 | 3.6599 | 2.1672 | 1.2996 |
| Internal test set | Central venous pressure | Transformer | 55 | 3.7055 | 2.2115 | 1.3262 |
| Internal test set | Central venous pressure | Transformer | 60 | 3.7451 | 2.2505 | 1.3494 |
| Internal test set | Central venous pressure | Transformer | 65 | 3.7814 | 2.2873 | 1.3714 |
| Internal test set | Central venous pressure | Transformer | 70 | 3.8143 | 2.3214 | 1.3918 |
| Internal test set | Central venous pressure | Transformer | 75 | 3.8472 | 2.3529 | 1.4107 |
| Internal test set | Central venous pressure | Transformer | 80 | 3.8777 | 2.3837 | 1.4292 |
| Internal test set | Central venous pressure | Transformer | 85 | 3.9069 | 2.4133 | 1.4469 |
| Internal test set | Central venous pressure | Transformer | 90 | 3.9338 | 2.4407 | 1.4631 |
| Internal test set | Central venous pressure | Transformer | 95 | 3.9580 | 2.4662 | 1.4782 |
| Internal test set | Central venous pressure | Transformer | 100 | 3.9821 | 2.4915 | 1.4931 |
| Internal test set | Central venous pressure | Transformer | 105 | 4.0044 | 2.5149 | 1.5069 |
| Internal test set | Central venous pressure | Transformer | 110 | 4.0269 | 2.5379 | 1.5206 |
| Internal test set | Central venous pressure | Transformer | 115 | 4.0482 | 2.5603 | 1.5339 |
| Internal test set | Central venous pressure | Transformer | 120 | 4.0698 | 2.5811 | 1.5461 |
| Internal test set | Diastolic BP | AR NNet | 5 | 5.1334 | 3.2656 | 1.9355 |
| Internal test set | Diastolic BP | AR NNet | 10 | 5.8604 | 3.8342 | 2.2734 |
| Internal test set | Diastolic BP | AR NNet | 15 | 6.2629 | 4.1760 | 2.4775 |
| Internal test set | Diastolic BP | AR NNet | 20 | 6.5481 | 4.4238 | 2.6257 |
| Internal test set | Diastolic BP | AR NNet | 25 | 6.7714 | 4.6205 | 2.7434 |
| Internal test set | Diastolic BP | AR NNet | 30 | 6.9483 | 4.7809 | 2.8394 |
| Internal test set | Diastolic BP | AR NNet | 35 | 7.1045 | 4.9214 | 2.9238 |
| Internal test set | Diastolic BP | AR NNet | 40 | 7.2333 | 5.0354 | 2.9923 |
| Internal test set | Diastolic BP | AR NNet | 45 | 7.3485 | 5.1371 | 3.0535 |
| Internal test set | Diastolic BP | AR NNet | 50 | 7.4477 | 5.2268 | 3.1075 |
| Internal test set | Diastolic BP | AR NNet | 55 | 7.5324 | 5.3053 | 3.1548 |
| Internal test set | Diastolic BP | AR NNet | 60 | 7.6114 | 5.3770 | 3.1981 |
| Internal test set | Diastolic BP | AR NNet | 65 | 7.6884 | 5.4458 | 3.2396 |
| Internal test set | Diastolic BP | AR NNet | 70 | 7.7544 | 5.5048 | 3.2751 |
| Internal test set | Diastolic BP | AR NNet | 75 | 7.8154 | 5.5590 | 3.3077 |
| Internal test set | Diastolic BP | AR NNet | 80 | 7.8718 | 5.6087 | 3.3377 |
| Internal test set | Diastolic BP | AR NNet | 85 | 7.9230 | 5.6554 | 3.3658 |
| Internal test set | Diastolic BP | AR NNet | 90 | 7.9682 | 5.6980 | 3.3916 |
| Internal test set | Diastolic BP | AR NNet | 95 | 8.0132 | 5.7400 | 3.4168 |
| Internal test set | Diastolic BP | AR NNet | 100 | 8.0570 | 5.7795 | 3.4406 |
| Internal test set | Diastolic BP | AR NNet | 105 | 8.0989 | 5.8174 | 3.4636 |
| Internal test set | Diastolic BP | AR NNet | 110 | 8.1398 | 5.8543 | 3.4860 |
| Internal test set | Diastolic BP | AR NNet | 115 | 8.1784 | 5.8892 | 3.5071 |
| Internal test set | Diastolic BP | AR NNet | 120 | 8.2161 | 5.9237 | 3.5279 |
| Internal test set | Diastolic BP | ARIMA | 5 | 4.9584 | 2.9445 | 1.7421 |
| Internal test set | Diastolic BP | ARIMA | 10 | 5.9259 | 3.7424 | 2.2170 |
| Internal test set | Diastolic BP | ARIMA | 15 | 6.4315 | 4.1827 | 2.4805 |
| Internal test set | Diastolic BP | ARIMA | 20 | 6.7769 | 4.4859 | 2.6622 |
| Internal test set | Diastolic BP | ARIMA | 25 | 7.0700 | 4.7349 | 2.8113 |
| Internal test set | Diastolic BP | ARIMA | 30 | 7.3100 | 4.9319 | 2.9296 |
| Internal test set | Diastolic BP | ARIMA | 35 | 7.5283 | 5.1093 | 3.0360 |
| Internal test set | Diastolic BP | ARIMA | 40 | 7.7128 | 5.2576 | 3.1250 |
| Internal test set | Diastolic BP | ARIMA | 45 | 7.8852 | 5.3891 | 3.2039 |
| Internal test set | Diastolic BP | ARIMA | 50 | 8.0418 | 5.5047 | 3.2741 |
| Internal test set | Diastolic BP | ARIMA | 55 | 8.1743 | 5.6068 | 3.3360 |
| Internal test set | Diastolic BP | ARIMA | 60 | 8.3032 | 5.7003 | 3.3928 |
| Internal test set | Diastolic BP | ARIMA | 65 | 8.4316 | 5.7919 | 3.4482 |
| Internal test set | Diastolic BP | ARIMA | 70 | 8.5438 | 5.8728 | 3.4972 |
| Internal test set | Diastolic BP | ARIMA | 75 | 8.6497 | 5.9475 | 3.5421 |
| Internal test set | Diastolic BP | ARIMA | 80 | 8.7582 | 6.0192 | 3.5854 |
| Internal test set | Diastolic BP | ARIMA | 85 | 8.8543 | 6.0820 | 3.6233 |
| Internal test set | Diastolic BP | ARIMA | 90 | 8.9465 | 6.1421 | 3.6598 |
| Internal test set | Diastolic BP | ARIMA | 95 | 9.0377 | 6.2032 | 3.6962 |
| Internal test set | Diastolic BP | ARIMA | 100 | 9.1271 | 6.2594 | 3.7303 |
| Internal test set | Diastolic BP | ARIMA | 105 | 9.2109 | 6.3126 | 3.7624 |
| Internal test set | Diastolic BP | ARIMA | 110 | 9.2994 | 6.3649 | 3.7944 |
| Internal test set | Diastolic BP | ARIMA | 115 | 9.3861 | 6.4140 | 3.8240 |
| Internal test set | Diastolic BP | ARIMA | 120 | 9.4633 | 6.4614 | 3.8524 |
| Internal test set | Diastolic BP | ETS | 5 | 4.9705 | 2.8817 | 1.7037 |
| Internal test set | Diastolic BP | ETS | 10 | 6.0225 | 3.6699 | 2.1720 |
| Internal test set | Diastolic BP | ETS | 15 | 6.6144 | 4.1591 | 2.4642 |
| Internal test set | Diastolic BP | ETS | 20 | 7.0384 | 4.5161 | 2.6773 |
| Internal test set | Diastolic BP | ETS | 25 | 7.3883 | 4.8053 | 2.8501 |
| Internal test set | Diastolic BP | ETS | 30 | 7.6773 | 5.0406 | 2.9905 |
| Internal test set | Diastolic BP | ETS | 35 | 7.9497 | 5.2538 | 3.1173 |
| Internal test set | Diastolic BP | ETS | 40 | 8.1734 | 5.4321 | 3.2232 |
| Internal test set | Diastolic BP | ETS | 45 | 8.3714 | 5.5877 | 3.3157 |
| Internal test set | Diastolic BP | ETS | 50 | 8.5580 | 5.7304 | 3.4013 |
| Internal test set | Diastolic BP | ETS | 55 | 8.7227 | 5.8532 | 3.4750 |
| Internal test set | Diastolic BP | ETS | 60 | 8.8710 | 5.9601 | 3.5392 |
| Internal test set | Diastolic BP | ETS | 65 | 9.0153 | 6.0639 | 3.6017 |
| Internal test set | Diastolic BP | ETS | 70 | 9.1423 | 6.1539 | 3.6563 |
| Internal test set | Diastolic BP | ETS | 75 | 9.2507 | 6.2327 | 3.7034 |
| Internal test set | Diastolic BP | ETS | 80 | 9.3624 | 6.3115 | 3.7508 |
| Internal test set | Diastolic BP | ETS | 85 | 9.4593 | 6.3800 | 3.7918 |
| Internal test set | Diastolic BP | ETS | 90 | 9.5541 | 6.4452 | 3.8312 |
| Internal test set | Diastolic BP | ETS | 95 | 9.6482 | 6.5097 | 3.8697 |
| Internal test set | Diastolic BP | ETS | 100 | 9.7341 | 6.5687 | 3.9051 |
| Internal test set | Diastolic BP | ETS | 105 | 9.8053 | 6.6204 | 3.9361 |
| Internal test set | Diastolic BP | ETS | 110 | 9.8939 | 6.6716 | 3.9670 |
| Internal test set | Diastolic BP | ETS | 115 | 9.9800 | 6.7258 | 3.9991 |
| Internal test set | Diastolic BP | ETS | 120 | 10.0634 | 6.7765 | 4.0294 |
| Internal test set | Diastolic BP | GRU | 5 | 4.5535 | 2.7313 | 1.6178 |
| Internal test set | Diastolic BP | GRU | 10 | 5.4311 | 3.4540 | 2.0485 |
| Internal test set | Diastolic BP | GRU | 15 | 5.8738 | 3.8541 | 2.2889 |
| Internal test set | Diastolic BP | GRU | 20 | 6.1797 | 4.1366 | 2.4588 |
| Internal test set | Diastolic BP | GRU | 25 | 6.4196 | 4.3547 | 2.5906 |
| Internal test set | Diastolic BP | GRU | 30 | 6.6173 | 4.5347 | 2.6996 |
| Internal test set | Diastolic BP | GRU | 35 | 6.7637 | 4.6757 | 2.7847 |
| Internal test set | Diastolic BP | GRU | 40 | 6.8797 | 4.7829 | 2.8490 |
| Internal test set | Diastolic BP | GRU | 45 | 6.9792 | 4.8764 | 2.9058 |
| Internal test set | Diastolic BP | GRU | 50 | 7.0714 | 4.9607 | 2.9569 |
| Internal test set | Diastolic BP | GRU | 55 | 7.1506 | 5.0315 | 2.9998 |
| Internal test set | Diastolic BP | GRU | 60 | 7.2214 | 5.0987 | 3.0406 |
| Internal test set | Diastolic BP | GRU | 65 | 7.2850 | 5.1568 | 3.0757 |
| Internal test set | Diastolic BP | GRU | 70 | 7.3363 | 5.2055 | 3.1053 |
| Internal test set | Diastolic BP | GRU | 75 | 7.3835 | 5.2523 | 3.1334 |
| Internal test set | Diastolic BP | GRU | 80 | 7.4262 | 5.2961 | 3.1601 |
| Internal test set | Diastolic BP | GRU | 85 | 7.4676 | 5.3342 | 3.1833 |
| Internal test set | Diastolic BP | GRU | 90 | 7.5080 | 5.3715 | 3.2055 |
| Internal test set | Diastolic BP | GRU | 95 | 7.5432 | 5.4076 | 3.2276 |
| Internal test set | Diastolic BP | GRU | 100 | 7.5777 | 5.4382 | 3.2458 |
| Internal test set | Diastolic BP | GRU | 105 | 7.6083 | 5.4666 | 3.2630 |
| Internal test set | Diastolic BP | GRU | 110 | 7.6378 | 5.4957 | 3.2809 |
| Internal test set | Diastolic BP | GRU | 115 | 7.6629 | 5.5222 | 3.2964 |
| Internal test set | Diastolic BP | GRU | 120 | 7.6889 | 5.5475 | 3.3117 |
| Internal test set | Diastolic BP | Naive | 5 | 5.0332 | 2.7843 | 1.6459 |
| Internal test set | Diastolic BP | Naive | 10 | 6.2338 | 3.6990 | 2.1872 |
| Internal test set | Diastolic BP | Naive | 15 | 6.8227 | 4.2161 | 2.4956 |
| Internal test set | Diastolic BP | Naive | 20 | 7.2217 | 4.5842 | 2.7148 |
| Internal test set | Diastolic BP | Naive | 25 | 7.5470 | 4.8791 | 2.8905 |
| Internal test set | Diastolic BP | Naive | 30 | 7.7936 | 5.1076 | 3.0268 |
| Internal test set | Diastolic BP | Naive | 35 | 8.0315 | 5.3156 | 3.1503 |
| Internal test set | Diastolic BP | Naive | 40 | 8.2141 | 5.4837 | 3.2496 |
| Internal test set | Diastolic BP | Naive | 45 | 8.3630 | 5.6250 | 3.3335 |
| Internal test set | Diastolic BP | Naive | 50 | 8.5128 | 5.7584 | 3.4133 |
| Internal test set | Diastolic BP | Naive | 55 | 8.6334 | 5.8718 | 3.4814 |
| Internal test set | Diastolic BP | Naive | 60 | 8.7409 | 5.9712 | 3.5414 |
| Internal test set | Diastolic BP | Naive | 65 | 8.8455 | 6.0643 | 3.5969 |
| Internal test set | Diastolic BP | Naive | 70 | 8.9318 | 6.1435 | 3.6450 |
| Internal test set | Diastolic BP | Naive | 75 | 8.9953 | 6.2097 | 3.6846 |
| Internal test set | Diastolic BP | Naive | 80 | 9.0655 | 6.2782 | 3.7257 |
| Internal test set | Diastolic BP | Naive | 85 | 9.1257 | 6.3368 | 3.7609 |
| Internal test set | Diastolic BP | Naive | 90 | 9.1801 | 6.3902 | 3.7932 |
| Internal test set | Diastolic BP | Naive | 95 | 9.2345 | 6.4449 | 3.8256 |
| Internal test set | Diastolic BP | Naive | 100 | 9.2893 | 6.4945 | 3.8554 |
| Internal test set | Diastolic BP | Naive | 105 | 9.3189 | 6.5330 | 3.8786 |
| Internal test set | Diastolic BP | Naive | 110 | 9.3553 | 6.5719 | 3.9025 |
| Internal test set | Diastolic BP | Naive | 115 | 9.3964 | 6.6123 | 3.9266 |
| Internal test set | Diastolic BP | Naive | 120 | 9.4417 | 6.6524 | 3.9506 |
| Internal test set | Diastolic BP | Theta | 5 | 4.9686 | 2.8861 | 1.7062 |
| Internal test set | Diastolic BP | Theta | 10 | 6.0280 | 3.6895 | 2.1837 |
| Internal test set | Diastolic BP | Theta | 15 | 6.6312 | 4.1940 | 2.4853 |
| Internal test set | Diastolic BP | Theta | 20 | 7.0743 | 4.5708 | 2.7102 |
| Internal test set | Diastolic BP | Theta | 25 | 7.4476 | 4.8820 | 2.8963 |
| Internal test set | Diastolic BP | Theta | 30 | 7.7625 | 5.1420 | 3.0516 |
| Internal test set | Diastolic BP | Theta | 35 | 8.0676 | 5.3830 | 3.1952 |
| Internal test set | Diastolic BP | Theta | 40 | 8.3295 | 5.5888 | 3.3176 |
| Internal test set | Diastolic BP | Theta | 45 | 8.5734 | 5.7763 | 3.4295 |
| Internal test set | Diastolic BP | Theta | 50 | 8.8046 | 5.9522 | 3.5351 |
| Internal test set | Diastolic BP | Theta | 55 | 9.0145 | 6.1100 | 3.6299 |
| Internal test set | Diastolic BP | Theta | 60 | 9.2131 | 6.2536 | 3.7162 |
| Internal test set | Diastolic BP | Theta | 65 | 9.4162 | 6.3956 | 3.8015 |
| Internal test set | Diastolic BP | Theta | 70 | 9.5962 | 6.5215 | 3.8776 |
| Internal test set | Diastolic BP | Theta | 75 | 9.7628 | 6.6376 | 3.9471 |
| Internal test set | Diastolic BP | Theta | 80 | 9.9353 | 6.7546 | 4.0174 |
| Internal test set | Diastolic BP | Theta | 85 | 10.1013 | 6.8639 | 4.0830 |
| Internal test set | Diastolic BP | Theta | 90 | 10.2673 | 6.9727 | 4.1484 |
| Internal test set | Diastolic BP | Theta | 95 | 10.4319 | 7.0794 | 4.2125 |
| Internal test set | Diastolic BP | Theta | 100 | 10.5911 | 7.1806 | 4.2735 |
| Internal test set | Diastolic BP | Theta | 105 | 10.7389 | 7.2774 | 4.3315 |
| Internal test set | Diastolic BP | Theta | 110 | 10.8973 | 7.3736 | 4.3892 |
| Internal test set | Diastolic BP | Theta | 115 | 11.0567 | 7.4717 | 4.4476 |
| Internal test set | Diastolic BP | Theta | 120 | 11.2156 | 7.5684 | 4.5052 |
| Internal test set | Diastolic BP | Transformer | 5 | 4.6310 | 2.7838 | 1.6513 |
| Internal test set | Diastolic BP | Transformer | 10 | 5.5245 | 3.5000 | 2.0773 |
| Internal test set | Diastolic BP | Transformer | 15 | 5.9551 | 3.8897 | 2.3112 |
| Internal test set | Diastolic BP | Transformer | 20 | 6.2528 | 4.1635 | 2.4757 |
| Internal test set | Diastolic BP | Transformer | 25 | 6.4875 | 4.3783 | 2.6053 |
| Internal test set | Diastolic BP | Transformer | 30 | 6.6747 | 4.5528 | 2.7109 |
| Internal test set | Diastolic BP | Transformer | 35 | 6.8177 | 4.6899 | 2.7934 |
| Internal test set | Diastolic BP | Transformer | 40 | 6.9301 | 4.7996 | 2.8594 |
| Internal test set | Diastolic BP | Transformer | 45 | 7.0250 | 4.8902 | 2.9143 |
| Internal test set | Diastolic BP | Transformer | 50 | 7.1151 | 4.9733 | 2.9646 |
| Internal test set | Diastolic BP | Transformer | 55 | 7.1926 | 5.0452 | 3.0082 |
| Internal test set | Diastolic BP | Transformer | 60 | 7.2623 | 5.1104 | 3.0478 |
| Internal test set | Diastolic BP | Transformer | 65 | 7.3245 | 5.1710 | 3.0845 |
| Internal test set | Diastolic BP | Transformer | 70 | 7.3736 | 5.2220 | 3.1157 |
| Internal test set | Diastolic BP | Transformer | 75 | 7.4173 | 5.2657 | 3.1421 |
| Internal test set | Diastolic BP | Transformer | 80 | 7.4571 | 5.3086 | 3.1680 |
| Internal test set | Diastolic BP | Transformer | 85 | 7.4967 | 5.3471 | 3.1913 |
| Internal test set | Diastolic BP | Transformer | 90 | 7.5335 | 5.3847 | 3.2141 |
| Internal test set | Diastolic BP | Transformer | 95 | 7.5720 | 5.4228 | 3.2371 |
| Internal test set | Diastolic BP | Transformer | 100 | 7.6071 | 5.4555 | 3.2569 |
| Internal test set | Diastolic BP | Transformer | 105 | 7.6330 | 5.4828 | 3.2733 |
| Internal test set | Diastolic BP | Transformer | 110 | 7.6617 | 5.5111 | 3.2906 |
| Internal test set | Diastolic BP | Transformer | 115 | 7.6871 | 5.5384 | 3.3069 |
| Internal test set | Diastolic BP | Transformer | 120 | 7.7114 | 5.5653 | 3.3230 |
| Internal test set | Heart rate | AR NNet | 5 | 4.7159 | 2.2614 | 1.3413 |
| Internal test set | Heart rate | AR NNet | 10 | 5.4062 | 2.6405 | 1.5652 |
| Internal test set | Heart rate | AR NNet | 15 | 5.8852 | 2.9284 | 1.7358 |
| Internal test set | Heart rate | AR NNet | 20 | 6.2674 | 3.1734 | 1.8811 |
| Internal test set | Heart rate | AR NNet | 25 | 6.6050 | 3.3948 | 2.0123 |
| Internal test set | Heart rate | AR NNet | 30 | 6.9003 | 3.5957 | 2.1313 |
| Internal test set | Heart rate | AR NNet | 35 | 7.1742 | 3.7865 | 2.2440 |
| Internal test set | Heart rate | AR NNet | 40 | 7.4078 | 3.9505 | 2.3410 |
| Internal test set | Heart rate | AR NNet | 45 | 7.6316 | 4.1022 | 2.4308 |
| Internal test set | Heart rate | AR NNet | 50 | 7.8271 | 4.2421 | 2.5138 |
| Internal test set | Heart rate | AR NNet | 55 | 8.0142 | 4.3750 | 2.5924 |
| Internal test set | Heart rate | AR NNet | 60 | 8.1896 | 4.5009 | 2.6668 |
| Internal test set | Heart rate | AR NNet | 65 | 8.3563 | 4.6212 | 2.7379 |
| Internal test set | Heart rate | AR NNet | 70 | 8.5112 | 4.7349 | 2.8052 |
| Internal test set | Heart rate | AR NNet | 75 | 8.6565 | 4.8430 | 2.8691 |
| Internal test set | Heart rate | AR NNet | 80 | 8.7945 | 4.9466 | 2.9305 |
| Internal test set | Heart rate | AR NNet | 85 | 8.9292 | 5.0463 | 2.9896 |
| Internal test set | Heart rate | AR NNet | 90 | 9.0596 | 5.1417 | 3.0461 |
| Internal test set | Heart rate | AR NNet | 95 | 9.1818 | 5.2318 | 3.0993 |
| Internal test set | Heart rate | AR NNet | 100 | 9.2952 | 5.3189 | 3.1508 |
| Internal test set | Heart rate | AR NNet | 105 | 9.4021 | 5.4027 | 3.2002 |
| Internal test set | Heart rate | AR NNet | 110 | 9.5125 | 5.4851 | 3.2489 |
| Internal test set | Heart rate | AR NNet | 115 | 9.6145 | 5.5656 | 3.2964 |
| Internal test set | Heart rate | AR NNet | 120 | 9.7168 | 5.6434 | 3.3423 |
| Internal test set | Heart rate | ARIMA | 5 | 4.5380 | 1.8988 | 1.1269 |
| Internal test set | Heart rate | ARIMA | 10 | 5.3617 | 2.3733 | 1.4059 |
| Internal test set | Heart rate | ARIMA | 15 | 5.9297 | 2.7071 | 1.6032 |
| Internal test set | Heart rate | ARIMA | 20 | 6.3588 | 2.9782 | 1.7637 |
| Internal test set | Heart rate | ARIMA | 25 | 6.7529 | 3.2245 | 1.9097 |
| Internal test set | Heart rate | ARIMA | 30 | 7.1032 | 3.4410 | 2.0380 |
| Internal test set | Heart rate | ARIMA | 35 | 7.4165 | 3.6418 | 2.1565 |
| Internal test set | Heart rate | ARIMA | 40 | 7.7022 | 3.8271 | 2.2662 |
| Internal test set | Heart rate | ARIMA | 45 | 7.9816 | 4.0020 | 2.3698 |
| Internal test set | Heart rate | ARIMA | 50 | 8.2415 | 4.1628 | 2.4649 |
| Internal test set | Heart rate | ARIMA | 55 | 8.4965 | 4.3178 | 2.5568 |
| Internal test set | Heart rate | ARIMA | 60 | 8.7297 | 4.4631 | 2.6426 |
| Internal test set | Heart rate | ARIMA | 65 | 8.9520 | 4.6037 | 2.7258 |
| Internal test set | Heart rate | ARIMA | 70 | 9.1474 | 4.7368 | 2.8042 |
| Internal test set | Heart rate | ARIMA | 75 | 9.3447 | 4.8665 | 2.8803 |
| Internal test set | Heart rate | ARIMA | 80 | 9.5421 | 4.9922 | 2.9544 |
| Internal test set | Heart rate | ARIMA | 85 | 9.7244 | 5.1117 | 3.0251 |
| Internal test set | Heart rate | ARIMA | 90 | 9.9076 | 5.2283 | 3.0939 |
| Internal test set | Heart rate | ARIMA | 95 | 10.0787 | 5.3419 | 3.1606 |
| Internal test set | Heart rate | ARIMA | 100 | 10.2459 | 5.4508 | 3.2249 |
| Internal test set | Heart rate | ARIMA | 105 | 10.3943 | 5.5554 | 3.2867 |
| Internal test set | Heart rate | ARIMA | 110 | 10.5565 | 5.6598 | 3.3483 |
| Internal test set | Heart rate | ARIMA | 115 | 10.7020 | 5.7616 | 3.4086 |
| Internal test set | Heart rate | ARIMA | 120 | 10.8580 | 5.8605 | 3.4669 |
| Internal test set | Heart rate | ETS | 5 | 4.4164 | 1.7926 | 1.0637 |
| Internal test set | Heart rate | ETS | 10 | 5.2979 | 2.2381 | 1.3257 |
| Internal test set | Heart rate | ETS | 15 | 5.9584 | 2.5654 | 1.5191 |
| Internal test set | Heart rate | ETS | 20 | 6.5246 | 2.8491 | 1.6870 |
| Internal test set | Heart rate | ETS | 25 | 7.0260 | 3.1006 | 1.8356 |
| Internal test set | Heart rate | ETS | 30 | 7.4665 | 3.3283 | 1.9703 |
| Internal test set | Heart rate | ETS | 35 | 7.8860 | 3.5381 | 2.0941 |
| Internal test set | Heart rate | ETS | 40 | 8.2792 | 3.7332 | 2.2094 |
| Internal test set | Heart rate | ETS | 45 | 8.6545 | 3.9169 | 2.3179 |
| Internal test set | Heart rate | ETS | 50 | 9.0312 | 4.0893 | 2.4197 |
| Internal test set | Heart rate | ETS | 55 | 9.3783 | 4.2512 | 2.5158 |
| Internal test set | Heart rate | ETS | 60 | 9.7037 | 4.4072 | 2.6080 |
| Internal test set | Heart rate | ETS | 65 | 10.0012 | 4.5552 | 2.6957 |
| Internal test set | Heart rate | ETS | 70 | 10.2711 | 4.6945 | 2.7776 |
| Internal test set | Heart rate | ETS | 75 | 10.5299 | 4.8287 | 2.8563 |
| Internal test set | Heart rate | ETS | 80 | 10.8000 | 4.9616 | 2.9344 |
| Internal test set | Heart rate | ETS | 85 | 11.0481 | 5.0866 | 3.0083 |
| Internal test set | Heart rate | ETS | 90 | 11.2871 | 5.2101 | 3.0813 |
| Internal test set | Heart rate | ETS | 95 | 11.5054 | 5.3259 | 3.1494 |
| Internal test set | Heart rate | ETS | 100 | 11.7297 | 5.4393 | 3.2161 |
| Internal test set | Heart rate | ETS | 105 | 11.9383 | 5.5492 | 3.2810 |
| Internal test set | Heart rate | ETS | 110 | 12.1522 | 5.6539 | 3.3429 |
| Internal test set | Heart rate | ETS | 115 | 12.3416 | 5.7567 | 3.4039 |
| Internal test set | Heart rate | ETS | 120 | 12.5363 | 5.8609 | 3.4656 |
| Internal test set | Heart rate | GRU | 5 | 4.2220 | 1.9657 | 1.1690 |
| Internal test set | Heart rate | GRU | 10 | 4.9944 | 2.3828 | 1.4146 |
| Internal test set | Heart rate | GRU | 15 | 5.4969 | 2.6933 | 1.5986 |
| Internal test set | Heart rate | GRU | 20 | 5.9073 | 2.9567 | 1.7551 |
| Internal test set | Heart rate | GRU | 25 | 6.2649 | 3.2013 | 1.9007 |
| Internal test set | Heart rate | GRU | 30 | 6.5816 | 3.4215 | 2.0323 |
| Internal test set | Heart rate | GRU | 35 | 6.8404 | 3.6128 | 2.1457 |
| Internal test set | Heart rate | GRU | 40 | 7.0622 | 3.7813 | 2.2455 |
| Internal test set | Heart rate | GRU | 45 | 7.2683 | 3.9370 | 2.3380 |
| Internal test set | Heart rate | GRU | 50 | 7.4623 | 4.0835 | 2.4250 |
| Internal test set | Heart rate | GRU | 55 | 7.6415 | 4.2243 | 2.5092 |
| Internal test set | Heart rate | GRU | 60 | 7.8061 | 4.3456 | 2.5810 |
| Internal test set | Heart rate | GRU | 65 | 7.9543 | 4.4726 | 2.6568 |
| Internal test set | Heart rate | GRU | 70 | 8.0937 | 4.5911 | 2.7271 |
| Internal test set | Heart rate | GRU | 75 | 8.2189 | 4.6930 | 2.7872 |
| Internal test set | Heart rate | GRU | 80 | 8.3429 | 4.8074 | 2.8550 |
| Internal test set | Heart rate | GRU | 85 | 8.4570 | 4.9009 | 2.9111 |
| Internal test set | Heart rate | GRU | 90 | 8.5709 | 4.9982 | 2.9691 |
| Internal test set | Heart rate | GRU | 95 | 8.6745 | 5.0862 | 3.0215 |
| Internal test set | Heart rate | GRU | 100 | 8.7732 | 5.1734 | 3.0731 |
| Internal test set | Heart rate | GRU | 105 | 8.8696 | 5.2570 | 3.1231 |
| Internal test set | Heart rate | GRU | 110 | 8.9702 | 5.3403 | 3.1720 |
| Internal test set | Heart rate | GRU | 115 | 9.0577 | 5.4135 | 3.2160 |
| Internal test set | Heart rate | GRU | 120 | 9.1434 | 5.4837 | 3.2569 |
| Internal test set | Heart rate | Naive | 5 | 4.4302 | 1.7130 | 1.0166 |
| Internal test set | Heart rate | Naive | 10 | 5.3699 | 2.1932 | 1.2987 |
| Internal test set | Heart rate | Naive | 15 | 5.9377 | 2.5178 | 1.4902 |
| Internal test set | Heart rate | Naive | 20 | 6.3795 | 2.7862 | 1.6495 |
| Internal test set | Heart rate | Naive | 25 | 6.7544 | 3.0235 | 1.7899 |
| Internal test set | Heart rate | Naive | 30 | 7.0763 | 3.2311 | 1.9129 |
| Internal test set | Heart rate | Naive | 35 | 7.3721 | 3.4255 | 2.0277 |
| Internal test set | Heart rate | Naive | 40 | 7.6201 | 3.6002 | 2.1309 |
| Internal test set | Heart rate | Naive | 45 | 7.8510 | 3.7610 | 2.2257 |
| Internal test set | Heart rate | Naive | 50 | 8.0809 | 3.9136 | 2.3162 |
| Internal test set | Heart rate | Naive | 55 | 8.2928 | 4.0547 | 2.3999 |
| Internal test set | Heart rate | Naive | 60 | 8.4910 | 4.1875 | 2.4783 |
| Internal test set | Heart rate | Naive | 65 | 8.6708 | 4.3144 | 2.5534 |
| Internal test set | Heart rate | Naive | 70 | 8.8364 | 4.4394 | 2.6274 |
| Internal test set | Heart rate | Naive | 75 | 8.9876 | 4.5517 | 2.6932 |
| Internal test set | Heart rate | Naive | 80 | 9.1337 | 4.6632 | 2.7588 |
| Internal test set | Heart rate | Naive | 85 | 9.2704 | 4.7670 | 2.8199 |
| Internal test set | Heart rate | Naive | 90 | 9.4076 | 4.8695 | 2.8810 |
| Internal test set | Heart rate | Naive | 95 | 9.5401 | 4.9708 | 2.9405 |
| Internal test set | Heart rate | Naive | 100 | 9.6634 | 5.0659 | 2.9966 |
| Internal test set | Heart rate | Naive | 105 | 9.7766 | 5.1565 | 3.0498 |
| Internal test set | Heart rate | Naive | 110 | 9.8965 | 5.2431 | 3.1009 |
| Internal test set | Heart rate | Naive | 115 | 10.0026 | 5.3292 | 3.1523 |
| Internal test set | Heart rate | Naive | 120 | 10.1094 | 5.4135 | 3.2022 |
| Internal test set | Heart rate | Theta | 5 | 4.3502 | 1.8068 | 1.0721 |
| Internal test set | Heart rate | Theta | 10 | 5.2085 | 2.2715 | 1.3456 |
| Internal test set | Heart rate | Theta | 15 | 5.7986 | 2.6150 | 1.5491 |
| Internal test set | Heart rate | Theta | 20 | 6.2896 | 2.9146 | 1.7269 |
| Internal test set | Heart rate | Theta | 25 | 6.7200 | 3.1835 | 1.8861 |
| Internal test set | Heart rate | Theta | 30 | 7.1172 | 3.4320 | 2.0335 |
| Internal test set | Heart rate | Theta | 35 | 7.4836 | 3.6647 | 2.1711 |
| Internal test set | Heart rate | Theta | 40 | 7.8171 | 3.8819 | 2.2995 |
| Internal test set | Heart rate | Theta | 45 | 8.1460 | 4.0911 | 2.4233 |
| Internal test set | Heart rate | Theta | 50 | 8.4597 | 4.2875 | 2.5396 |
| Internal test set | Heart rate | Theta | 55 | 8.7638 | 4.4775 | 2.6523 |
| Internal test set | Heart rate | Theta | 60 | 9.0529 | 4.6614 | 2.7612 |
| Internal test set | Heart rate | Theta | 65 | 9.3235 | 4.8389 | 2.8664 |
| Internal test set | Heart rate | Theta | 70 | 9.5893 | 5.0131 | 2.9693 |
| Internal test set | Heart rate | Theta | 75 | 9.8522 | 5.1812 | 3.0683 |
| Internal test set | Heart rate | Theta | 80 | 10.1036 | 5.3461 | 3.1656 |
| Internal test set | Heart rate | Theta | 85 | 10.3494 | 5.5069 | 3.2609 |
| Internal test set | Heart rate | Theta | 90 | 10.5889 | 5.6645 | 3.3544 |
| Internal test set | Heart rate | Theta | 95 | 10.8212 | 5.8185 | 3.4454 |
| Internal test set | Heart rate | Theta | 100 | 11.0537 | 5.9684 | 3.5340 |
| Internal test set | Heart rate | Theta | 105 | 11.2812 | 6.1158 | 3.6213 |
| Internal test set | Heart rate | Theta | 110 | 11.5122 | 6.2603 | 3.7070 |
| Internal test set | Heart rate | Theta | 115 | 11.7340 | 6.4038 | 3.7921 |
| Internal test set | Heart rate | Theta | 120 | 11.9664 | 6.5482 | 3.8776 |
| Internal test set | Heart rate | Transformer | 5 | 4.3456 | 2.1793 | 1.3010 |
| Internal test set | Heart rate | Transformer | 10 | 5.1366 | 2.5957 | 1.5456 |
| Internal test set | Heart rate | Transformer | 15 | 5.6375 | 2.8957 | 1.7229 |
| Internal test set | Heart rate | Transformer | 20 | 6.0409 | 3.1464 | 1.8716 |
| Internal test set | Heart rate | Transformer | 25 | 6.3948 | 3.3769 | 2.0085 |
| Internal test set | Heart rate | Transformer | 30 | 6.7085 | 3.5864 | 2.1332 |
| Internal test set | Heart rate | Transformer | 35 | 6.9640 | 3.7649 | 2.2386 |
| Internal test set | Heart rate | Transformer | 40 | 7.1848 | 3.9250 | 2.3335 |
| Internal test set | Heart rate | Transformer | 45 | 7.3857 | 4.0719 | 2.4204 |
| Internal test set | Heart rate | Transformer | 50 | 7.5806 | 4.2127 | 2.5037 |
| Internal test set | Heart rate | Transformer | 55 | 7.7591 | 4.3419 | 2.5806 |
| Internal test set | Heart rate | Transformer | 60 | 7.9256 | 4.4640 | 2.6528 |
| Internal test set | Heart rate | Transformer | 65 | 8.0733 | 4.5795 | 2.7213 |
| Internal test set | Heart rate | Transformer | 70 | 8.2127 | 4.6909 | 2.7874 |
| Internal test set | Heart rate | Transformer | 75 | 8.3376 | 4.7931 | 2.8479 |
| Internal test set | Heart rate | Transformer | 80 | 8.4580 | 4.8936 | 2.9074 |
| Internal test set | Heart rate | Transformer | 85 | 8.5733 | 4.9886 | 2.9636 |
| Internal test set | Heart rate | Transformer | 90 | 8.6864 | 5.0807 | 3.0187 |
| Internal test set | Heart rate | Transformer | 95 | 8.7922 | 5.1664 | 3.0692 |
| Internal test set | Heart rate | Transformer | 100 | 8.8926 | 5.2504 | 3.1190 |
| Internal test set | Heart rate | Transformer | 105 | 8.9899 | 5.3310 | 3.1667 |
| Internal test set | Heart rate | Transformer | 110 | 9.0863 | 5.4081 | 3.2124 |
| Internal test set | Heart rate | Transformer | 115 | 9.1751 | 5.4836 | 3.2573 |
| Internal test set | Heart rate | Transformer | 120 | 9.2631 | 5.5573 | 3.3008 |
| Internal test set | Mean BP | AR NNet | 5 | 6.4860 | 4.2327 | 2.4843 |
| Internal test set | Mean BP | AR NNet | 10 | 7.4713 | 5.0249 | 2.9498 |
| Internal test set | Mean BP | AR NNet | 15 | 8.0199 | 5.4972 | 3.2286 |
| Internal test set | Mean BP | AR NNet | 20 | 8.4055 | 5.8364 | 3.4290 |
| Internal test set | Mean BP | AR NNet | 25 | 8.7016 | 6.1005 | 3.5852 |
| Internal test set | Mean BP | AR NNet | 30 | 8.9345 | 6.3129 | 3.7111 |
| Internal test set | Mean BP | AR NNet | 35 | 9.1332 | 6.4944 | 3.8189 |
| Internal test set | Mean BP | AR NNet | 40 | 9.2962 | 6.6415 | 3.9063 |
| Internal test set | Mean BP | AR NNet | 45 | 9.4380 | 6.7699 | 3.9825 |
| Internal test set | Mean BP | AR NNet | 50 | 9.5632 | 6.8829 | 4.0497 |
| Internal test set | Mean BP | AR NNet | 55 | 9.6727 | 6.9813 | 4.1083 |
| Internal test set | Mean BP | AR NNet | 60 | 9.7669 | 7.0684 | 4.1602 |
| Internal test set | Mean BP | AR NNet | 65 | 9.8570 | 7.1506 | 4.2094 |
| Internal test set | Mean BP | AR NNet | 70 | 9.9324 | 7.2220 | 4.2520 |
| Internal test set | Mean BP | AR NNet | 75 | 10.0008 | 7.2857 | 4.2899 |
| Internal test set | Mean BP | AR NNet | 80 | 10.0625 | 7.3460 | 4.3259 |
| Internal test set | Mean BP | AR NNet | 85 | 10.1163 | 7.3983 | 4.3573 |
| Internal test set | Mean BP | AR NNet | 90 | 10.1641 | 7.4465 | 4.3862 |
| Internal test set | Mean BP | AR NNet | 95 | 10.2115 | 7.4938 | 4.4145 |
| Internal test set | Mean BP | AR NNet | 100 | 10.2554 | 7.5382 | 4.4411 |
| Internal test set | Mean BP | AR NNet | 105 | 10.2968 | 7.5799 | 4.4663 |
| Internal test set | Mean BP | AR NNet | 110 | 10.3367 | 7.6203 | 4.4908 |
| Internal test set | Mean BP | AR NNet | 115 | 10.3760 | 7.6581 | 4.5136 |
| Internal test set | Mean BP | AR NNet | 120 | 10.4156 | 7.6960 | 4.5365 |
| Internal test set | Mean BP | ARIMA | 5 | 6.1846 | 3.7499 | 2.1967 |
| Internal test set | Mean BP | ARIMA | 10 | 7.5399 | 4.8872 | 2.8656 |
| Internal test set | Mean BP | ARIMA | 15 | 8.2325 | 5.5000 | 3.2278 |
| Internal test set | Mean BP | ARIMA | 20 | 8.7032 | 5.9196 | 3.4764 |
| Internal test set | Mean BP | ARIMA | 25 | 9.0865 | 6.2517 | 3.6729 |
| Internal test set | Mean BP | ARIMA | 30 | 9.4039 | 6.5176 | 3.8312 |
| Internal test set | Mean BP | ARIMA | 35 | 9.6826 | 6.7492 | 3.9689 |
| Internal test set | Mean BP | ARIMA | 40 | 9.9252 | 6.9436 | 4.0846 |
| Internal test set | Mean BP | ARIMA | 45 | 10.1467 | 7.1163 | 4.1872 |
| Internal test set | Mean BP | ARIMA | 50 | 10.3508 | 7.2674 | 4.2774 |
| Internal test set | Mean BP | ARIMA | 55 | 10.5340 | 7.4031 | 4.3585 |
| Internal test set | Mean BP | ARIMA | 60 | 10.6974 | 7.5219 | 4.4295 |
| Internal test set | Mean BP | ARIMA | 65 | 10.8468 | 7.6322 | 4.4958 |
| Internal test set | Mean BP | ARIMA | 70 | 10.9967 | 7.7347 | 4.5573 |
| Internal test set | Mean BP | ARIMA | 75 | 11.1261 | 7.8254 | 4.6115 |
| Internal test set | Mean BP | ARIMA | 80 | 11.2508 | 7.9138 | 4.6647 |
| Internal test set | Mean BP | ARIMA | 85 | 11.3659 | 7.9916 | 4.7114 |
| Internal test set | Mean BP | ARIMA | 90 | 11.4749 | 8.0608 | 4.7530 |
| Internal test set | Mean BP | ARIMA | 95 | 11.5870 | 8.1317 | 4.7956 |
| Internal test set | Mean BP | ARIMA | 100 | 11.6832 | 8.1946 | 4.8335 |
| Internal test set | Mean BP | ARIMA | 105 | 11.7734 | 8.2557 | 4.8701 |
| Internal test set | Mean BP | ARIMA | 110 | 11.8649 | 8.3143 | 4.9058 |
| Internal test set | Mean BP | ARIMA | 115 | 11.9602 | 8.3719 | 4.9407 |
| Internal test set | Mean BP | ARIMA | 120 | 12.0480 | 8.4263 | 4.9737 |
| Internal test set | Mean BP | ETS | 5 | 6.2038 | 3.6743 | 2.1509 |
| Internal test set | Mean BP | ETS | 10 | 7.6446 | 4.7880 | 2.8060 |
| Internal test set | Mean BP | ETS | 15 | 8.4662 | 5.4715 | 3.2093 |
| Internal test set | Mean BP | ETS | 20 | 9.0497 | 5.9746 | 3.5061 |
| Internal test set | Mean BP | ETS | 25 | 9.5254 | 6.3760 | 3.7429 |
| Internal test set | Mean BP | ETS | 30 | 9.9192 | 6.7063 | 3.9378 |
| Internal test set | Mean BP | ETS | 35 | 10.2755 | 6.9968 | 4.1086 |
| Internal test set | Mean BP | ETS | 40 | 10.5658 | 7.2349 | 4.2488 |
| Internal test set | Mean BP | ETS | 45 | 10.8324 | 7.4446 | 4.3722 |
| Internal test set | Mean BP | ETS | 50 | 11.0803 | 7.6339 | 4.4838 |
| Internal test set | Mean BP | ETS | 55 | 11.2996 | 7.7987 | 4.5814 |
| Internal test set | Mean BP | ETS | 60 | 11.4883 | 7.9417 | 4.6666 |
| Internal test set | Mean BP | ETS | 65 | 11.6715 | 8.0765 | 4.7467 |
| Internal test set | Mean BP | ETS | 70 | 11.8132 | 8.1861 | 4.8123 |
| Internal test set | Mean BP | ETS | 75 | 11.9381 | 8.2837 | 4.8700 |
| Internal test set | Mean BP | ETS | 80 | 12.0634 | 8.3835 | 4.9297 |
| Internal test set | Mean BP | ETS | 85 | 12.1656 | 8.4627 | 4.9769 |
| Internal test set | Mean BP | ETS | 90 | 12.2757 | 8.5393 | 5.0221 |
| Internal test set | Mean BP | ETS | 95 | 12.3924 | 8.6217 | 5.0708 |
| Internal test set | Mean BP | ETS | 100 | 12.4913 | 8.6907 | 5.1120 |
| Internal test set | Mean BP | ETS | 105 | 12.5637 | 8.7519 | 5.1487 |
| Internal test set | Mean BP | ETS | 110 | 12.6432 | 8.8080 | 5.1826 |
| Internal test set | Mean BP | ETS | 115 | 12.7377 | 8.8727 | 5.2213 |
| Internal test set | Mean BP | ETS | 120 | 12.8237 | 8.9315 | 5.2564 |
| Internal test set | Mean BP | GRU | 5 | 5.6311 | 3.4627 | 2.0365 |
| Internal test set | Mean BP | GRU | 10 | 6.8572 | 4.4862 | 2.6386 |
| Internal test set | Mean BP | GRU | 15 | 7.4695 | 5.0536 | 2.9758 |
| Internal test set | Mean BP | GRU | 20 | 7.8875 | 5.4525 | 3.2126 |
| Internal test set | Mean BP | GRU | 25 | 8.2122 | 5.7630 | 3.3979 |
| Internal test set | Mean BP | GRU | 30 | 8.4738 | 6.0135 | 3.5476 |
| Internal test set | Mean BP | GRU | 35 | 8.6599 | 6.1967 | 3.6567 |
| Internal test set | Mean BP | GRU | 40 | 8.8113 | 6.3477 | 3.7468 |
| Internal test set | Mean BP | GRU | 45 | 8.9368 | 6.4739 | 3.8227 |
| Internal test set | Mean BP | GRU | 50 | 9.0491 | 6.5810 | 3.8864 |
| Internal test set | Mean BP | GRU | 55 | 9.1435 | 6.6714 | 3.9407 |
| Internal test set | Mean BP | GRU | 60 | 9.2240 | 6.7543 | 3.9908 |
| Internal test set | Mean BP | GRU | 65 | 9.2942 | 6.8245 | 4.0330 |
| Internal test set | Mean BP | GRU | 70 | 9.3532 | 6.8876 | 4.0711 |
| Internal test set | Mean BP | GRU | 75 | 9.4066 | 6.9404 | 4.1026 |
| Internal test set | Mean BP | GRU | 80 | 9.4550 | 6.9946 | 4.1354 |
| Internal test set | Mean BP | GRU | 85 | 9.4994 | 7.0429 | 4.1645 |
| Internal test set | Mean BP | GRU | 90 | 9.5417 | 7.0863 | 4.1907 |
| Internal test set | Mean BP | GRU | 95 | 9.5785 | 7.1256 | 4.2141 |
| Internal test set | Mean BP | GRU | 100 | 9.6128 | 7.1609 | 4.2353 |
| Internal test set | Mean BP | GRU | 105 | 9.6397 | 7.1947 | 4.2556 |
| Internal test set | Mean BP | GRU | 110 | 9.6660 | 7.2238 | 4.2730 |
| Internal test set | Mean BP | GRU | 115 | 9.6878 | 7.2492 | 4.2886 |
| Internal test set | Mean BP | GRU | 120 | 9.7144 | 7.2805 | 4.3074 |
| Internal test set | Mean BP | Naive | 5 | 6.2261 | 3.5294 | 2.0656 |
| Internal test set | Mean BP | Naive | 10 | 7.8691 | 4.8017 | 2.8113 |
| Internal test set | Mean BP | Naive | 15 | 8.6751 | 5.5206 | 3.2351 |
| Internal test set | Mean BP | Naive | 20 | 9.2221 | 6.0375 | 3.5393 |
| Internal test set | Mean BP | Naive | 25 | 9.6580 | 6.4422 | 3.7774 |
| Internal test set | Mean BP | Naive | 30 | 10.0073 | 6.7685 | 3.9697 |
| Internal test set | Mean BP | Naive | 35 | 10.3236 | 7.0552 | 4.1382 |
| Internal test set | Mean BP | Naive | 40 | 10.5723 | 7.2854 | 4.2733 |
| Internal test set | Mean BP | Naive | 45 | 10.7707 | 7.4741 | 4.3842 |
| Internal test set | Mean BP | Naive | 50 | 10.9653 | 7.6503 | 4.4878 |
| Internal test set | Mean BP | Naive | 55 | 11.1376 | 7.8030 | 4.5781 |
| Internal test set | Mean BP | Naive | 60 | 11.2807 | 7.9351 | 4.6570 |
| Internal test set | Mean BP | Naive | 65 | 11.4122 | 8.0565 | 4.7288 |
| Internal test set | Mean BP | Naive | 70 | 11.5204 | 8.1565 | 4.7887 |
| Internal test set | Mean BP | Naive | 75 | 11.5941 | 8.2406 | 4.8385 |
| Internal test set | Mean BP | Naive | 80 | 11.6796 | 8.3301 | 4.8920 |
| Internal test set | Mean BP | Naive | 85 | 11.7389 | 8.3979 | 4.9322 |
| Internal test set | Mean BP | Naive | 90 | 11.7962 | 8.4585 | 4.9679 |
| Internal test set | Mean BP | Naive | 95 | 11.8628 | 8.5255 | 5.0072 |
| Internal test set | Mean BP | Naive | 100 | 11.9274 | 8.5855 | 5.0426 |
| Internal test set | Mean BP | Naive | 105 | 11.9545 | 8.6298 | 5.0693 |
| Internal test set | Mean BP | Naive | 110 | 11.9879 | 8.6731 | 5.0955 |
| Internal test set | Mean BP | Naive | 115 | 12.0311 | 8.7227 | 5.1254 |
| Internal test set | Mean BP | Naive | 120 | 12.0764 | 8.7702 | 5.1542 |
| Internal test set | Mean BP | Theta | 5 | 6.2027 | 3.6770 | 2.1521 |
| Internal test set | Mean BP | Theta | 10 | 7.6651 | 4.8116 | 2.8196 |
| Internal test set | Mean BP | Theta | 15 | 8.4971 | 5.5164 | 3.2357 |
| Internal test set | Mean BP | Theta | 20 | 9.1061 | 6.0441 | 3.5472 |
| Internal test set | Mean BP | Theta | 25 | 9.6147 | 6.4773 | 3.8029 |
| Internal test set | Mean BP | Theta | 30 | 10.0505 | 6.8416 | 4.0181 |
| Internal test set | Mean BP | Theta | 35 | 10.4548 | 7.1699 | 4.2115 |
| Internal test set | Mean BP | Theta | 40 | 10.8047 | 7.4478 | 4.3753 |
| Internal test set | Mean BP | Theta | 45 | 11.1305 | 7.6983 | 4.5231 |
| Internal test set | Mean BP | Theta | 50 | 11.4451 | 7.9336 | 4.6621 |
| Internal test set | Mean BP | Theta | 55 | 11.7335 | 8.1476 | 4.7888 |
| Internal test set | Mean BP | Theta | 60 | 12.0017 | 8.3395 | 4.9031 |
| Internal test set | Mean BP | Theta | 65 | 12.2631 | 8.5245 | 5.0129 |
| Internal test set | Mean BP | Theta | 70 | 12.4949 | 8.6863 | 5.1095 |
| Internal test set | Mean BP | Theta | 75 | 12.7083 | 8.8346 | 5.1975 |
| Internal test set | Mean BP | Theta | 80 | 12.9269 | 8.9859 | 5.2877 |
| Internal test set | Mean BP | Theta | 85 | 13.1288 | 9.1189 | 5.3667 |
| Internal test set | Mean BP | Theta | 90 | 13.3315 | 9.2512 | 5.4452 |
| Internal test set | Mean BP | Theta | 95 | 13.5447 | 9.3920 | 5.5286 |
| Internal test set | Mean BP | Theta | 100 | 13.7441 | 9.5227 | 5.6061 |
| Internal test set | Mean BP | Theta | 105 | 13.9250 | 9.6438 | 5.6784 |
| Internal test set | Mean BP | Theta | 110 | 14.1105 | 9.7620 | 5.7487 |
| Internal test set | Mean BP | Theta | 115 | 14.3067 | 9.8851 | 5.8219 |
| Internal test set | Mean BP | Theta | 120 | 14.5003 | 10.0057 | 5.8935 |
| Internal test set | Mean BP | Transformer | 5 | 5.7419 | 3.5084 | 2.0610 |
| Internal test set | Mean BP | Transformer | 10 | 6.9936 | 4.5424 | 2.6696 |
| Internal test set | Mean BP | Transformer | 15 | 7.5908 | 5.0987 | 2.9994 |
| Internal test set | Mean BP | Transformer | 20 | 7.9975 | 5.4901 | 3.2318 |
| Internal test set | Mean BP | Transformer | 25 | 8.3115 | 5.7915 | 3.4114 |
| Internal test set | Mean BP | Transformer | 30 | 8.5577 | 6.0322 | 3.5553 |
| Internal test set | Mean BP | Transformer | 35 | 8.7413 | 6.2185 | 3.6662 |
| Internal test set | Mean BP | Transformer | 40 | 8.8853 | 6.3648 | 3.7535 |
| Internal test set | Mean BP | Transformer | 45 | 9.0039 | 6.4843 | 3.8250 |
| Internal test set | Mean BP | Transformer | 50 | 9.1108 | 6.5911 | 3.8889 |
| Internal test set | Mean BP | Transformer | 55 | 9.2026 | 6.6811 | 3.9429 |
| Internal test set | Mean BP | Transformer | 60 | 9.2803 | 6.7623 | 3.9920 |
| Internal test set | Mean BP | Transformer | 65 | 9.3499 | 6.8362 | 4.0366 |
| Internal test set | Mean BP | Transformer | 70 | 9.4040 | 6.8973 | 4.0736 |
| Internal test set | Mean BP | Transformer | 75 | 9.4530 | 6.9501 | 4.1054 |
| Internal test set | Mean BP | Transformer | 80 | 9.4990 | 7.0021 | 4.1368 |
| Internal test set | Mean BP | Transformer | 85 | 9.5384 | 7.0475 | 4.1642 |
| Internal test set | Mean BP | Transformer | 90 | 9.5767 | 7.0887 | 4.1890 |
| Internal test set | Mean BP | Transformer | 95 | 9.6151 | 7.1312 | 4.2146 |
| Internal test set | Mean BP | Transformer | 100 | 9.6489 | 7.1680 | 4.2367 |
| Internal test set | Mean BP | Transformer | 105 | 9.6730 | 7.1986 | 4.2553 |
| Internal test set | Mean BP | Transformer | 110 | 9.6984 | 7.2300 | 4.2742 |
| Internal test set | Mean BP | Transformer | 115 | 9.7218 | 7.2601 | 4.2924 |
| Internal test set | Mean BP | Transformer | 120 | 9.7431 | 7.2866 | 4.3085 |
| Internal test set | SpO2 | AR NNet | 5 | 1.8145 | 0.8484 | 0.5151 |
| Internal test set | SpO2 | AR NNet | 10 | 2.0452 | 0.9875 | 0.5997 |
| Internal test set | SpO2 | AR NNet | 15 | 2.1812 | 1.0773 | 0.6544 |
| Internal test set | SpO2 | AR NNet | 20 | 2.2812 | 1.1466 | 0.6965 |
| Internal test set | SpO2 | AR NNet | 25 | 2.3582 | 1.2034 | 0.7311 |
| Internal test set | SpO2 | AR NNet | 30 | 2.4240 | 1.2525 | 0.7611 |
| Internal test set | SpO2 | AR NNet | 35 | 2.4814 | 1.2970 | 0.7883 |
| Internal test set | SpO2 | AR NNet | 40 | 2.5327 | 1.3353 | 0.8116 |
| Internal test set | SpO2 | AR NNet | 45 | 2.5796 | 1.3697 | 0.8326 |
| Internal test set | SpO2 | AR NNet | 50 | 2.6224 | 1.4011 | 0.8518 |
| Internal test set | SpO2 | AR NNet | 55 | 2.6609 | 1.4298 | 0.8694 |
| Internal test set | SpO2 | AR NNet | 60 | 2.6943 | 1.4555 | 0.8852 |
| Internal test set | SpO2 | AR NNet | 65 | 2.7254 | 1.4789 | 0.8995 |
| Internal test set | SpO2 | AR NNet | 70 | 2.7572 | 1.5012 | 0.9133 |
| Internal test set | SpO2 | AR NNet | 75 | 2.7862 | 1.5223 | 0.9263 |
| Internal test set | SpO2 | AR NNet | 80 | 2.8143 | 1.5420 | 0.9384 |
| Internal test set | SpO2 | AR NNet | 85 | 2.8366 | 1.5599 | 0.9494 |
| Internal test set | SpO2 | AR NNet | 90 | 2.8566 | 1.5766 | 0.9598 |
| Internal test set | SpO2 | AR NNet | 95 | 2.8765 | 1.5926 | 0.9697 |
| Internal test set | SpO2 | AR NNet | 100 | 2.8976 | 1.6085 | 0.9794 |
| Internal test set | SpO2 | AR NNet | 105 | 2.9190 | 1.6235 | 0.9887 |
| Internal test set | SpO2 | AR NNet | 110 | 2.9402 | 1.6379 | 0.9975 |
| Internal test set | SpO2 | AR NNet | 115 | 2.9626 | 1.6519 | 1.0061 |
| Internal test set | SpO2 | AR NNet | 120 | 2.9864 | 1.6657 | 1.0147 |
| Internal test set | SpO2 | ARIMA | 5 | 1.8648 | 0.7768 | 0.4713 |
| Internal test set | SpO2 | ARIMA | 10 | 2.1641 | 0.9578 | 0.5814 |
| Internal test set | SpO2 | ARIMA | 15 | 2.3272 | 1.0680 | 0.6485 |
| Internal test set | SpO2 | ARIMA | 20 | 2.4483 | 1.1485 | 0.6973 |
| Internal test set | SpO2 | ARIMA | 25 | 2.5432 | 1.2153 | 0.7378 |
| Internal test set | SpO2 | ARIMA | 30 | 2.6189 | 1.2703 | 0.7715 |
| Internal test set | SpO2 | ARIMA | 35 | 2.6917 | 1.3202 | 0.8018 |
| Internal test set | SpO2 | ARIMA | 40 | 2.7490 | 1.3627 | 0.8278 |
| Internal test set | SpO2 | ARIMA | 45 | 2.8067 | 1.4037 | 0.8528 |
| Internal test set | SpO2 | ARIMA | 50 | 2.8594 | 1.4403 | 0.8752 |
| Internal test set | SpO2 | ARIMA | 55 | 2.9070 | 1.4753 | 0.8967 |
| Internal test set | SpO2 | ARIMA | 60 | 2.9505 | 1.5059 | 0.9155 |
| Internal test set | SpO2 | ARIMA | 65 | 2.9876 | 1.5338 | 0.9326 |
| Internal test set | SpO2 | ARIMA | 70 | 3.0255 | 1.5593 | 0.9485 |
| Internal test set | SpO2 | ARIMA | 75 | 3.0646 | 1.5844 | 0.9639 |
| Internal test set | SpO2 | ARIMA | 80 | 3.0992 | 1.6079 | 0.9782 |
| Internal test set | SpO2 | ARIMA | 85 | 3.1307 | 1.6303 | 0.9921 |
| Internal test set | SpO2 | ARIMA | 90 | 3.1600 | 1.6501 | 1.0043 |
| Internal test set | SpO2 | ARIMA | 95 | 3.1851 | 1.6698 | 1.0162 |
| Internal test set | SpO2 | ARIMA | 100 | 3.2101 | 1.6889 | 1.0279 |
| Internal test set | SpO2 | ARIMA | 105 | 3.2404 | 1.7084 | 1.0399 |
| Internal test set | SpO2 | ARIMA | 110 | 3.2638 | 1.7252 | 1.0501 |
| Internal test set | SpO2 | ARIMA | 115 | 3.2895 | 1.7424 | 1.0607 |
| Internal test set | SpO2 | ARIMA | 120 | 3.3199 | 1.7581 | 1.0703 |
| Internal test set | SpO2 | ETS | 5 | 1.8445 | 0.7347 | 0.4453 |
| Internal test set | SpO2 | ETS | 10 | 2.2022 | 0.9098 | 0.5519 |
| Internal test set | SpO2 | ETS | 15 | 2.4175 | 1.0268 | 0.6231 |
| Internal test set | SpO2 | ETS | 20 | 2.5810 | 1.1195 | 0.6792 |
| Internal test set | SpO2 | ETS | 25 | 2.7148 | 1.1964 | 0.7260 |
| Internal test set | SpO2 | ETS | 30 | 2.8247 | 1.2615 | 0.7657 |
| Internal test set | SpO2 | ETS | 35 | 2.9193 | 1.3183 | 0.8002 |
| Internal test set | SpO2 | ETS | 40 | 3.0098 | 1.3704 | 0.8319 |
| Internal test set | SpO2 | ETS | 45 | 3.0937 | 1.4181 | 0.8609 |
| Internal test set | SpO2 | ETS | 50 | 3.1750 | 1.4640 | 0.8888 |
| Internal test set | SpO2 | ETS | 55 | 3.2416 | 1.5050 | 0.9140 |
| Internal test set | SpO2 | ETS | 60 | 3.3038 | 1.5413 | 0.9363 |
| Internal test set | SpO2 | ETS | 65 | 3.3553 | 1.5737 | 0.9559 |
| Internal test set | SpO2 | ETS | 70 | 3.4045 | 1.6046 | 0.9749 |
| Internal test set | SpO2 | ETS | 75 | 3.4581 | 1.6353 | 0.9936 |
| Internal test set | SpO2 | ETS | 80 | 3.5115 | 1.6636 | 1.0109 |
| Internal test set | SpO2 | ETS | 85 | 3.5628 | 1.6905 | 1.0275 |
| Internal test set | SpO2 | ETS | 90 | 3.6098 | 1.7154 | 1.0430 |
| Internal test set | SpO2 | ETS | 95 | 3.6500 | 1.7385 | 1.0568 |
| Internal test set | SpO2 | ETS | 100 | 3.6887 | 1.7609 | 1.0705 |
| Internal test set | SpO2 | ETS | 105 | 3.7265 | 1.7815 | 1.0831 |
| Internal test set | SpO2 | ETS | 110 | 3.7655 | 1.8020 | 1.0955 |
| Internal test set | SpO2 | ETS | 115 | 3.8032 | 1.8224 | 1.1079 |
| Internal test set | SpO2 | ETS | 120 | 3.8411 | 1.8412 | 1.1193 |
| Internal test set | SpO2 | GRU | 5 | 1.7160 | 0.8149 | 0.4958 |
| Internal test set | SpO2 | GRU | 10 | 1.9567 | 0.9724 | 0.5918 |
| Internal test set | SpO2 | GRU | 15 | 2.0895 | 1.0712 | 0.6523 |
| Internal test set | SpO2 | GRU | 20 | 2.1842 | 1.1450 | 0.6976 |
| Internal test set | SpO2 | GRU | 25 | 2.2615 | 1.2054 | 0.7347 |
| Internal test set | SpO2 | GRU | 30 | 2.3225 | 1.2507 | 0.7625 |
| Internal test set | SpO2 | GRU | 35 | 2.3746 | 1.2940 | 0.7889 |
| Internal test set | SpO2 | GRU | 40 | 2.4207 | 1.3307 | 0.8114 |
| Internal test set | SpO2 | GRU | 45 | 2.4632 | 1.3641 | 0.8317 |
| Internal test set | SpO2 | GRU | 50 | 2.4994 | 1.3952 | 0.8506 |
| Internal test set | SpO2 | GRU | 55 | 2.5323 | 1.4244 | 0.8684 |
| Internal test set | SpO2 | GRU | 60 | 2.5632 | 1.4493 | 0.8837 |
| Internal test set | SpO2 | GRU | 65 | 2.5892 | 1.4726 | 0.8979 |
| Internal test set | SpO2 | GRU | 70 | 2.6148 | 1.4958 | 0.9120 |
| Internal test set | SpO2 | GRU | 75 | 2.6396 | 1.5171 | 0.9251 |
| Internal test set | SpO2 | GRU | 80 | 2.6627 | 1.5356 | 0.9364 |
| Internal test set | SpO2 | GRU | 85 | 2.6826 | 1.5529 | 0.9471 |
| Internal test set | SpO2 | GRU | 90 | 2.7006 | 1.5694 | 0.9570 |
| Internal test set | SpO2 | GRU | 95 | 2.7181 | 1.5836 | 0.9657 |
| Internal test set | SpO2 | GRU | 100 | 2.7333 | 1.5983 | 0.9749 |
| Internal test set | SpO2 | GRU | 105 | 2.7487 | 1.6093 | 0.9817 |
| Internal test set | SpO2 | GRU | 110 | 2.7610 | 1.6240 | 0.9907 |
| Internal test set | SpO2 | GRU | 115 | 2.7755 | 1.6352 | 0.9977 |
| Internal test set | SpO2 | GRU | 120 | 2.7886 | 1.6445 | 1.0034 |
| Internal test set | SpO2 | Naive | 5 | 1.8537 | 0.6642 | 0.4033 |
| Internal test set | SpO2 | Naive | 10 | 2.2119 | 0.8718 | 0.5292 |
| Internal test set | SpO2 | Naive | 15 | 2.3982 | 0.9981 | 0.6059 |
| Internal test set | SpO2 | Naive | 20 | 2.5304 | 1.0953 | 0.6647 |
| Internal test set | SpO2 | Naive | 25 | 2.6373 | 1.1732 | 0.7120 |
| Internal test set | SpO2 | Naive | 30 | 2.7249 | 1.2388 | 0.7520 |
| Internal test set | SpO2 | Naive | 35 | 2.7966 | 1.2946 | 0.7860 |
| Internal test set | SpO2 | Naive | 40 | 2.8590 | 1.3458 | 0.8171 |
| Internal test set | SpO2 | Naive | 45 | 2.9153 | 1.3904 | 0.8441 |
| Internal test set | SpO2 | Naive | 50 | 2.9758 | 1.4343 | 0.8709 |
| Internal test set | SpO2 | Naive | 55 | 3.0223 | 1.4730 | 0.8948 |
| Internal test set | SpO2 | Naive | 60 | 3.0644 | 1.5084 | 0.9164 |
| Internal test set | SpO2 | Naive | 65 | 3.0964 | 1.5383 | 0.9346 |
| Internal test set | SpO2 | Naive | 70 | 3.1309 | 1.5676 | 0.9527 |
| Internal test set | SpO2 | Naive | 75 | 3.1613 | 1.5948 | 0.9693 |
| Internal test set | SpO2 | Naive | 80 | 3.1987 | 1.6214 | 0.9853 |
| Internal test set | SpO2 | Naive | 85 | 3.2253 | 1.6446 | 0.9997 |
| Internal test set | SpO2 | Naive | 90 | 3.2494 | 1.6664 | 1.0133 |
| Internal test set | SpO2 | Naive | 95 | 3.2745 | 1.6879 | 1.0263 |
| Internal test set | SpO2 | Naive | 100 | 3.2948 | 1.7076 | 1.0382 |
| Internal test set | SpO2 | Naive | 105 | 3.3167 | 1.7263 | 1.0497 |
| Internal test set | SpO2 | Naive | 110 | 3.3360 | 1.7435 | 1.0603 |
| Internal test set | SpO2 | Naive | 115 | 3.3607 | 1.7614 | 1.0712 |
| Internal test set | SpO2 | Naive | 120 | 3.3878 | 1.7790 | 1.0819 |
| Internal test set | SpO2 | Theta | 5 | 1.8063 | 0.7303 | 0.4427 |
| Internal test set | SpO2 | Theta | 10 | 2.1204 | 0.9051 | 0.5491 |
| Internal test set | SpO2 | Theta | 15 | 2.3005 | 1.0215 | 0.6200 |
| Internal test set | SpO2 | Theta | 20 | 2.4368 | 1.1139 | 0.6761 |
| Internal test set | SpO2 | Theta | 25 | 2.5483 | 1.1905 | 0.7228 |
| Internal test set | SpO2 | Theta | 30 | 2.6430 | 1.2566 | 0.7631 |
| Internal test set | SpO2 | Theta | 35 | 2.7250 | 1.3141 | 0.7981 |
| Internal test set | SpO2 | Theta | 40 | 2.7982 | 1.3673 | 0.8305 |
| Internal test set | SpO2 | Theta | 45 | 2.8682 | 1.4167 | 0.8607 |
| Internal test set | SpO2 | Theta | 50 | 2.9390 | 1.4642 | 0.8897 |
| Internal test set | SpO2 | Theta | 55 | 2.9972 | 1.5072 | 0.9162 |
| Internal test set | SpO2 | Theta | 60 | 3.0501 | 1.5456 | 0.9397 |
| Internal test set | SpO2 | Theta | 65 | 3.0945 | 1.5802 | 0.9609 |
| Internal test set | SpO2 | Theta | 70 | 3.1399 | 1.6136 | 0.9815 |
| Internal test set | SpO2 | Theta | 75 | 3.1856 | 1.6467 | 1.0017 |
| Internal test set | SpO2 | Theta | 80 | 3.2337 | 1.6780 | 1.0208 |
| Internal test set | SpO2 | Theta | 85 | 3.2748 | 1.7069 | 1.0386 |
| Internal test set | SpO2 | Theta | 90 | 3.3112 | 1.7341 | 1.0555 |
| Internal test set | SpO2 | Theta | 95 | 3.3467 | 1.7598 | 1.0710 |
| Internal test set | SpO2 | Theta | 100 | 3.3828 | 1.7850 | 1.0864 |
| Internal test set | SpO2 | Theta | 105 | 3.4191 | 1.8088 | 1.1009 |
| Internal test set | SpO2 | Theta | 110 | 3.4542 | 1.8323 | 1.1152 |
| Internal test set | SpO2 | Theta | 115 | 3.4934 | 1.8555 | 1.1295 |
| Internal test set | SpO2 | Theta | 120 | 3.5338 | 1.8782 | 1.1435 |
| Internal test set | SpO2 | Transformer | 5 | 1.7329 | 0.8103 | 0.4924 |
| Internal test set | SpO2 | Transformer | 10 | 1.9739 | 0.9620 | 0.5849 |
| Internal test set | SpO2 | Transformer | 15 | 2.1017 | 1.0552 | 0.6420 |
| Internal test set | SpO2 | Transformer | 20 | 2.1924 | 1.1276 | 0.6863 |
| Internal test set | SpO2 | Transformer | 25 | 2.2656 | 1.1848 | 0.7214 |
| Internal test set | SpO2 | Transformer | 30 | 2.3228 | 1.2322 | 0.7505 |
| Internal test set | SpO2 | Transformer | 35 | 2.3705 | 1.2722 | 0.7750 |
| Internal test set | SpO2 | Transformer | 40 | 2.4142 | 1.3092 | 0.7976 |
| Internal test set | SpO2 | Transformer | 45 | 2.4543 | 1.3413 | 0.8173 |
| Internal test set | SpO2 | Transformer | 50 | 2.4885 | 1.3701 | 0.8349 |
| Internal test set | SpO2 | Transformer | 55 | 2.5204 | 1.3981 | 0.8520 |
| Internal test set | SpO2 | Transformer | 60 | 2.5481 | 1.4225 | 0.8670 |
| Internal test set | SpO2 | Transformer | 65 | 2.5743 | 1.4453 | 0.8809 |
| Internal test set | SpO2 | Transformer | 70 | 2.5970 | 1.4665 | 0.8940 |
| Internal test set | SpO2 | Transformer | 75 | 2.6198 | 1.4866 | 0.9064 |
| Internal test set | SpO2 | Transformer | 80 | 2.6424 | 1.5050 | 0.9176 |
| Internal test set | SpO2 | Transformer | 85 | 2.6613 | 1.5217 | 0.9280 |
| Internal test set | SpO2 | Transformer | 90 | 2.6795 | 1.5371 | 0.9375 |
| Internal test set | SpO2 | Transformer | 95 | 2.6960 | 1.5523 | 0.9468 |
| Internal test set | SpO2 | Transformer | 100 | 2.7106 | 1.5667 | 0.9557 |
| Internal test set | SpO2 | Transformer | 105 | 2.7260 | 1.5801 | 0.9639 |
| Internal test set | SpO2 | Transformer | 110 | 2.7370 | 1.5924 | 0.9716 |
| Internal test set | SpO2 | Transformer | 115 | 2.7506 | 1.6040 | 0.9788 |
| Internal test set | SpO2 | Transformer | 120 | 2.7654 | 1.6157 | 0.9861 |
| Internal test set | Systolic BP | AR NNet | 5 | 11.1504 | 7.2891 | 4.2268 |
| Internal test set | Systolic BP | AR NNet | 10 | 12.8851 | 8.6509 | 5.0166 |
| Internal test set | Systolic BP | AR NNet | 15 | 13.8576 | 9.4693 | 5.4927 |
| Internal test set | Systolic BP | AR NNet | 20 | 14.5427 | 10.0627 | 5.8387 |
| Internal test set | Systolic BP | AR NNet | 25 | 15.0776 | 10.5336 | 6.1128 |
| Internal test set | Systolic BP | AR NNet | 30 | 15.5020 | 10.9130 | 6.3339 |
| Internal test set | Systolic BP | AR NNet | 35 | 15.8544 | 11.2350 | 6.5217 |
| Internal test set | Systolic BP | AR NNet | 40 | 16.1545 | 11.4997 | 6.6758 |
| Internal test set | Systolic BP | AR NNet | 45 | 16.4154 | 11.7329 | 6.8114 |
| Internal test set | Systolic BP | AR NNet | 50 | 16.6409 | 11.9362 | 6.9300 |
| Internal test set | Systolic BP | AR NNet | 55 | 16.8383 | 12.1130 | 7.0335 |
| Internal test set | Systolic BP | AR NNet | 60 | 17.0119 | 12.2730 | 7.1272 |
| Internal test set | Systolic BP | AR NNet | 65 | 17.1781 | 12.4219 | 7.2143 |
| Internal test set | Systolic BP | AR NNet | 70 | 17.3182 | 12.5521 | 7.2904 |
| Internal test set | Systolic BP | AR NNet | 75 | 17.4490 | 12.6721 | 7.3605 |
| Internal test set | Systolic BP | AR NNet | 80 | 17.5642 | 12.7805 | 7.4240 |
| Internal test set | Systolic BP | AR NNet | 85 | 17.6713 | 12.8817 | 7.4831 |
| Internal test set | Systolic BP | AR NNet | 90 | 17.7638 | 12.9731 | 7.5367 |
| Internal test set | Systolic BP | AR NNet | 95 | 17.8582 | 13.0632 | 7.5894 |
| Internal test set | Systolic BP | AR NNet | 100 | 17.9453 | 13.1454 | 7.6375 |
| Internal test set | Systolic BP | AR NNet | 105 | 18.0299 | 13.2254 | 7.6845 |
| Internal test set | Systolic BP | AR NNet | 110 | 18.1112 | 13.2994 | 7.7279 |
| Internal test set | Systolic BP | AR NNet | 115 | 18.1928 | 13.3751 | 7.7722 |
| Internal test set | Systolic BP | AR NNet | 120 | 18.2669 | 13.4457 | 7.8138 |
| Internal test set | Systolic BP | ARIMA | 5 | 10.6287 | 6.4846 | 3.7542 |
| Internal test set | Systolic BP | ARIMA | 10 | 12.9656 | 8.4095 | 4.8725 |
| Internal test set | Systolic BP | ARIMA | 15 | 14.1735 | 9.4563 | 5.4823 |
| Internal test set | Systolic BP | ARIMA | 20 | 15.0123 | 10.1802 | 5.9047 |
| Internal test set | Systolic BP | ARIMA | 25 | 15.6981 | 10.7660 | 6.2463 |
| Internal test set | Systolic BP | ARIMA | 30 | 16.2674 | 11.2406 | 6.5234 |
| Internal test set | Systolic BP | ARIMA | 35 | 16.7738 | 11.6568 | 6.7657 |
| Internal test set | Systolic BP | ARIMA | 40 | 17.1997 | 11.9950 | 6.9632 |
| Internal test set | Systolic BP | ARIMA | 45 | 17.6064 | 12.3043 | 7.1433 |
| Internal test set | Systolic BP | ARIMA | 50 | 17.9740 | 12.5800 | 7.3039 |
| Internal test set | Systolic BP | ARIMA | 55 | 18.2904 | 12.8183 | 7.4437 |
| Internal test set | Systolic BP | ARIMA | 60 | 18.5805 | 13.0358 | 7.5717 |
| Internal test set | Systolic BP | ARIMA | 65 | 18.8758 | 13.2428 | 7.6926 |
| Internal test set | Systolic BP | ARIMA | 70 | 19.1394 | 13.4267 | 7.8008 |
| Internal test set | Systolic BP | ARIMA | 75 | 19.3971 | 13.5961 | 7.9001 |
| Internal test set | Systolic BP | ARIMA | 80 | 19.6350 | 13.7530 | 7.9927 |
| Internal test set | Systolic BP | ARIMA | 85 | 19.8555 | 13.8997 | 8.0794 |
| Internal test set | Systolic BP | ARIMA | 90 | 20.0687 | 14.0344 | 8.1586 |
| Internal test set | Systolic BP | ARIMA | 95 | 20.2904 | 14.1685 | 8.2360 |
| Internal test set | Systolic BP | ARIMA | 100 | 20.4927 | 14.2885 | 8.3062 |
| Internal test set | Systolic BP | ARIMA | 105 | 20.6965 | 14.4040 | 8.3734 |
| Internal test set | Systolic BP | ARIMA | 110 | 20.8949 | 14.5181 | 8.4400 |
| Internal test set | Systolic BP | ARIMA | 115 | 21.0863 | 14.6299 | 8.5054 |
| Internal test set | Systolic BP | ARIMA | 120 | 21.2588 | 14.7339 | 8.5664 |
| Internal test set | Systolic BP | ETS | 5 | 10.6495 | 6.3574 | 3.6792 |
| Internal test set | Systolic BP | ETS | 10 | 13.1506 | 8.2560 | 4.7818 |
| Internal test set | Systolic BP | ETS | 15 | 14.5999 | 9.4266 | 5.4629 |
| Internal test set | Systolic BP | ETS | 20 | 15.6440 | 10.2915 | 5.9663 |
| Internal test set | Systolic BP | ETS | 25 | 16.4967 | 10.9933 | 6.3757 |
| Internal test set | Systolic BP | ETS | 30 | 17.2175 | 11.5771 | 6.7151 |
| Internal test set | Systolic BP | ETS | 35 | 17.8586 | 12.0902 | 7.0125 |
| Internal test set | Systolic BP | ETS | 40 | 18.4039 | 12.5064 | 7.2541 |
| Internal test set | Systolic BP | ETS | 45 | 18.9240 | 12.8833 | 7.4713 |
| Internal test set | Systolic BP | ETS | 50 | 19.3721 | 13.2140 | 7.6625 |
| Internal test set | Systolic BP | ETS | 55 | 19.7390 | 13.4988 | 7.8286 |
| Internal test set | Systolic BP | ETS | 60 | 20.0853 | 13.7595 | 7.9808 |
| Internal test set | Systolic BP | ETS | 65 | 20.4330 | 14.0114 | 8.1277 |
| Internal test set | Systolic BP | ETS | 70 | 20.7130 | 14.2111 | 8.2446 |
| Internal test set | Systolic BP | ETS | 75 | 20.9630 | 14.3980 | 8.3540 |
| Internal test set | Systolic BP | ETS | 80 | 21.2004 | 14.5717 | 8.4561 |
| Internal test set | Systolic BP | ETS | 85 | 21.4266 | 14.7286 | 8.5480 |
| Internal test set | Systolic BP | ETS | 90 | 21.6462 | 14.8687 | 8.6295 |
| Internal test set | Systolic BP | ETS | 95 | 21.8567 | 15.0134 | 8.7128 |
| Internal test set | Systolic BP | ETS | 100 | 22.0443 | 15.1377 | 8.7850 |
| Internal test set | Systolic BP | ETS | 105 | 22.2178 | 15.2573 | 8.8544 |
| Internal test set | Systolic BP | ETS | 110 | 22.4082 | 15.3727 | 8.9208 |
| Internal test set | Systolic BP | ETS | 115 | 22.6101 | 15.4966 | 8.9930 |
| Internal test set | Systolic BP | ETS | 120 | 22.7956 | 15.6086 | 9.0591 |
| Internal test set | Systolic BP | GRU | 5 | 9.7487 | 6.0169 | 3.5027 |
| Internal test set | Systolic BP | GRU | 10 | 11.8562 | 7.7678 | 4.5203 |
| Internal test set | Systolic BP | GRU | 15 | 12.9298 | 8.7508 | 5.0944 |
| Internal test set | Systolic BP | GRU | 20 | 13.6680 | 9.4421 | 5.4987 |
| Internal test set | Systolic BP | GRU | 25 | 14.2426 | 9.9863 | 5.8187 |
| Internal test set | Systolic BP | GRU | 30 | 14.7196 | 10.4310 | 6.0807 |
| Internal test set | Systolic BP | GRU | 35 | 15.0650 | 10.7617 | 6.2739 |
| Internal test set | Systolic BP | GRU | 40 | 15.3502 | 11.0411 | 6.4379 |
| Internal test set | Systolic BP | GRU | 45 | 15.5910 | 11.2688 | 6.5719 |
| Internal test set | Systolic BP | GRU | 50 | 15.7990 | 11.4654 | 6.6868 |
| Internal test set | Systolic BP | GRU | 55 | 15.9718 | 11.6397 | 6.7902 |
| Internal test set | Systolic BP | GRU | 60 | 16.1308 | 11.7911 | 6.8793 |
| Internal test set | Systolic BP | GRU | 65 | 16.2667 | 11.9278 | 6.9607 |
| Internal test set | Systolic BP | GRU | 70 | 16.3901 | 12.0466 | 7.0308 |
| Internal test set | Systolic BP | GRU | 75 | 16.4963 | 12.1554 | 7.0948 |
| Internal test set | Systolic BP | GRU | 80 | 16.5967 | 12.2552 | 7.1530 |
| Internal test set | Systolic BP | GRU | 85 | 16.6855 | 12.3528 | 7.2121 |
| Internal test set | Systolic BP | GRU | 90 | 16.7693 | 12.4346 | 7.2605 |
| Internal test set | Systolic BP | GRU | 95 | 16.8432 | 12.5042 | 7.3004 |
| Internal test set | Systolic BP | GRU | 100 | 16.9107 | 12.5748 | 7.3417 |
| Internal test set | Systolic BP | GRU | 105 | 16.9702 | 12.6340 | 7.3766 |
| Internal test set | Systolic BP | GRU | 110 | 17.0334 | 12.7033 | 7.4176 |
| Internal test set | Systolic BP | GRU | 115 | 17.0947 | 12.7634 | 7.4533 |
| Internal test set | Systolic BP | GRU | 120 | 17.1504 | 12.8161 | 7.4840 |
| Internal test set | Systolic BP | Naive | 5 | 10.6977 | 6.1478 | 3.5591 |
| Internal test set | Systolic BP | Naive | 10 | 13.5137 | 8.3045 | 4.8076 |
| Internal test set | Systolic BP | Naive | 15 | 14.9373 | 9.5312 | 5.5199 |
| Internal test set | Systolic BP | Naive | 20 | 15.8984 | 10.4145 | 6.0330 |
| Internal test set | Systolic BP | Naive | 25 | 16.6681 | 11.1175 | 6.4415 |
| Internal test set | Systolic BP | Naive | 30 | 17.2794 | 11.6839 | 6.7710 |
| Internal test set | Systolic BP | Naive | 35 | 17.8493 | 12.1913 | 7.0646 |
| Internal test set | Systolic BP | Naive | 40 | 18.2871 | 12.5916 | 7.2959 |
| Internal test set | Systolic BP | Naive | 45 | 18.6839 | 12.9311 | 7.4909 |
| Internal test set | Systolic BP | Naive | 50 | 19.0314 | 13.2427 | 7.6707 |
| Internal test set | Systolic BP | Naive | 55 | 19.3074 | 13.5044 | 7.8236 |
| Internal test set | Systolic BP | Naive | 60 | 19.5492 | 13.7393 | 7.9611 |
| Internal test set | Systolic BP | Naive | 65 | 19.7912 | 13.9655 | 8.0930 |
| Internal test set | Systolic BP | Naive | 70 | 19.9707 | 14.1426 | 8.1961 |
| Internal test set | Systolic BP | Naive | 75 | 20.1231 | 14.3054 | 8.2912 |
| Internal test set | Systolic BP | Naive | 80 | 20.2710 | 14.4542 | 8.3785 |
| Internal test set | Systolic BP | Naive | 85 | 20.3921 | 14.5855 | 8.4557 |
| Internal test set | Systolic BP | Naive | 90 | 20.4908 | 14.6979 | 8.5220 |
| Internal test set | Systolic BP | Naive | 95 | 20.6224 | 14.8233 | 8.5939 |
| Internal test set | Systolic BP | Naive | 100 | 20.7205 | 14.9309 | 8.6565 |
| Internal test set | Systolic BP | Naive | 105 | 20.7922 | 15.0137 | 8.7040 |
| Internal test set | Systolic BP | Naive | 110 | 20.8769 | 15.1042 | 8.7560 |
| Internal test set | Systolic BP | Naive | 115 | 20.9739 | 15.1990 | 8.8114 |
| Internal test set | Systolic BP | Naive | 120 | 21.0574 | 15.2865 | 8.8636 |
| Internal test set | Systolic BP | Theta | 5 | 10.6440 | 6.3583 | 3.6789 |
| Internal test set | Systolic BP | Theta | 10 | 13.1710 | 8.2949 | 4.8044 |
| Internal test set | Systolic BP | Theta | 15 | 14.6235 | 9.4968 | 5.5039 |
| Internal test set | Systolic BP | Theta | 20 | 15.6897 | 10.4046 | 6.0331 |
| Internal test set | Systolic BP | Theta | 25 | 16.5678 | 11.1538 | 6.4701 |
| Internal test set | Systolic BP | Theta | 30 | 17.3288 | 11.7883 | 6.8396 |
| Internal test set | Systolic BP | Theta | 35 | 18.0304 | 12.3594 | 7.1714 |
| Internal test set | Systolic BP | Theta | 40 | 18.6322 | 12.8364 | 7.4488 |
| Internal test set | Systolic BP | Theta | 45 | 19.2139 | 13.2721 | 7.7012 |
| Internal test set | Systolic BP | Theta | 50 | 19.7442 | 13.6751 | 7.9352 |
| Internal test set | Systolic BP | Theta | 55 | 20.2107 | 14.0368 | 8.1470 |
| Internal test set | Systolic BP | Theta | 60 | 20.6549 | 14.3719 | 8.3434 |
| Internal test set | Systolic BP | Theta | 65 | 21.1000 | 14.7016 | 8.5363 |
| Internal test set | Systolic BP | Theta | 70 | 21.4853 | 14.9831 | 8.7015 |
| Internal test set | Systolic BP | Theta | 75 | 21.8533 | 15.2477 | 8.8571 |
| Internal test set | Systolic BP | Theta | 80 | 22.2188 | 15.5070 | 9.0095 |
| Internal test set | Systolic BP | Theta | 85 | 22.5690 | 15.7513 | 9.1529 |
| Internal test set | Systolic BP | Theta | 90 | 22.9139 | 15.9858 | 9.2903 |
| Internal test set | Systolic BP | Theta | 95 | 23.2733 | 16.2280 | 9.4316 |
| Internal test set | Systolic BP | Theta | 100 | 23.5985 | 16.4517 | 9.5623 |
| Internal test set | Systolic BP | Theta | 105 | 23.9165 | 16.6633 | 9.6859 |
| Internal test set | Systolic BP | Theta | 110 | 24.2487 | 16.8808 | 9.8124 |
| Internal test set | Systolic BP | Theta | 115 | 24.5970 | 17.1017 | 9.9415 |
| Internal test set | Systolic BP | Theta | 120 | 24.9281 | 17.3103 | 10.0645 |
| Internal test set | Systolic BP | Transformer | 5 | 9.9716 | 6.2128 | 3.6125 |
| Internal test set | Systolic BP | Transformer | 10 | 12.1082 | 7.9727 | 4.6346 |
| Internal test set | Systolic BP | Transformer | 15 | 13.1517 | 8.9190 | 5.1872 |
| Internal test set | Systolic BP | Transformer | 20 | 13.8663 | 9.5861 | 5.5780 |
| Internal test set | Systolic BP | Transformer | 25 | 14.4208 | 10.1094 | 5.8856 |
| Internal test set | Systolic BP | Transformer | 30 | 14.8676 | 10.5323 | 6.1347 |
| Internal test set | Systolic BP | Transformer | 35 | 15.2068 | 10.8571 | 6.3252 |
| Internal test set | Systolic BP | Transformer | 40 | 15.4755 | 11.1186 | 6.4787 |
| Internal test set | Systolic BP | Transformer | 45 | 15.7047 | 11.3399 | 6.6085 |
| Internal test set | Systolic BP | Transformer | 50 | 15.9008 | 11.5307 | 6.7208 |
| Internal test set | Systolic BP | Transformer | 55 | 16.0666 | 11.6928 | 6.8169 |
| Internal test set | Systolic BP | Transformer | 60 | 16.2157 | 11.8398 | 6.9040 |
| Internal test set | Systolic BP | Transformer | 65 | 16.3487 | 11.9749 | 6.9842 |
| Internal test set | Systolic BP | Transformer | 70 | 16.4592 | 12.0907 | 7.0529 |
| Internal test set | Systolic BP | Transformer | 75 | 16.5614 | 12.1932 | 7.1138 |
| Internal test set | Systolic BP | Transformer | 80 | 16.6550 | 12.2909 | 7.1715 |
| Internal test set | Systolic BP | Transformer | 85 | 16.7344 | 12.3781 | 7.2232 |
| Internal test set | Systolic BP | Transformer | 90 | 16.8145 | 12.4596 | 7.2714 |
| Internal test set | Systolic BP | Transformer | 95 | 16.8911 | 12.5361 | 7.3165 |
| Internal test set | Systolic BP | Transformer | 100 | 16.9563 | 12.6047 | 7.3571 |
| Internal test set | Systolic BP | Transformer | 105 | 17.0147 | 12.6639 | 7.3917 |
| Internal test set | Systolic BP | Transformer | 110 | 17.0763 | 12.7251 | 7.4272 |
| Internal test set | Systolic BP | Transformer | 115 | 17.1333 | 12.7822 | 7.4610 |
| Internal test set | Systolic BP | Transformer | 120 | 17.1884 | 12.8361 | 7.4924 |
