# Supplementary material for: Short-term vital parameter forecasting in the intensive care unit: A benchmark study leveraging data from patients after cardiothoracic surgery
Source: PLOS Digit Health. 2024 Sep 12;3(9):e0000598. doi: 10.1371/journal.pdig.0000598 (PMC11392423; doi:10.1371/journal.pdig.0000598)
Supplement: S4 Table — (DOCX) [file pdig.0000598.s005.docx]

**S4 Table:** Optimal hyperparameters of the GRU model

| **Model** | **Hyperparameter** | **Value** |
| --- | --- | --- |
| GRU | Learning rate | 0.00403 |
|  | Weight decay | 0.000008 |
|  | Dropout | 0.13 |
|  | Number of layers | 1 |
|  | Hidden size | 128 |
