# Supplementary material for: Short-term vital parameter forecasting in the intensive care unit: A benchmark study leveraging data from patients after cardiothoracic surgery
Source: PLOS Digit Health. 2024 Sep 12;3(9):e0000598. doi: 10.1371/journal.pdig.0000598 (PMC11392423; doi:10.1371/journal.pdig.0000598)
Supplement: S5 Table — (DOCX) [file pdig.0000598.s006.docx]

**S5 Table:** Optimal hyperparameters of the N-BEATS model per vital parameter

| **Model** | **Vital parameter** | **Hyperparameter** | **Value** |
| --- | --- | --- | --- |
| N-BEATS | BP Diastolic | Learning rate | 0.0028 |
|  |  | MLP units | 128 |
|  |  | Input size | 72 |
|  |  | Polynomial degree | 2 |
| N-BEATS | BP Systolic | Learning rate | 0.00317 |
|  |  | MLP units | 64 |
|  |  | Input size | 48 |
|  |  | Polynomial degree | 2 |
| N-BEATS | BP Mean | Learning rate | 0.0028 |
|  |  | MLP units | 128 |
|  |  | Input size | 72 |
|  |  | Polynomial degree | 2 |
| N-BEATS | Central venous pressure | Learning rate | 0.00151 |
|  |  | MLP units | 128 |
|  |  | Input size | 72 |
|  |  | Polynomial degree | 4 |
| N-BEATS | Oxygen saturation | Learning rate | 0.00101 |
|  |  | MLP units | 128 |
|  |  | Input size | 24 |
|  |  | Polynomial degree | 2 |
| N-BEATS | Heart rate | Learning rate | 0.00329 |
|  |  | MLP units | 64 |
|  |  | Input size | 12 |
|  |  | Polynomial degree | 3 |
