# Supplementary material for: Short-term vital parameter forecasting in the intensive care unit: A benchmark study leveraging data from patients after cardiothoracic surgery
Source: PLOS Digit Health. 2024 Sep 12;3(9):e0000598. doi: 10.1371/journal.pdig.0000598 (PMC11392423; doi:10.1371/journal.pdig.0000598)
Supplement: S6 Table — (DOCX) [file pdig.0000598.s007.docx]

**S6 Table:** Optimal hyperparameters of the N-HiTS model per vital parameter

| **Model** | **Vital parameter** | **Hyperparameter** | **Value** |
| --- | --- | --- | --- |
| N-HiTS | BP Diastolic | Learning rate | 0.00281 |
|  |  | MLP units | 32 |
|  |  | Input size | 48 |
|  |  | Dropout | 0.18 |
| N-HiTS | BP Systolic | Learning rate | 0.00336 |
|  |  | MLP units | 64 |
|  |  | Input size | 24 |
|  |  | Dropout | 0.07 |
| N-HiTS | BP Mean | Learning rate | 0.00173 |
|  |  | MLP units | 64 |
|  |  | Input size | 72 |
|  |  | Dropout | 0.24 |
| N-HiTS | Central venous pressure | Learning rate | 0.00089 |
|  |  | MLP units | 64 |
|  |  | Input size | 12 |
|  |  | Dropout | 0.01 |
| N-HiTS | Oxygen saturation | Learning rate | 0.00101 |
|  |  | MLP units | 64 |
|  |  | Input size | 12 |
|  |  | Dropout | 0.01 |
| N-HiTS | Heart rate | Learning rate | 0.00313 |
|  |  | MLP units | 128 |
|  |  | Input size | 48 |
|  |  | Dropout | 0.13 |
