# Supplementary material for: Short-term vital parameter forecasting in the intensive care unit: A benchmark study leveraging data from patients after cardiothoracic surgery
Source: PLOS Digit Health. 2024 Sep 12;3(9):e0000598. doi: 10.1371/journal.pdig.0000598 (PMC11392423; doi:10.1371/journal.pdig.0000598)
Supplement: Attachment — Submitted filename: forecasting data in the icu vital parameters.docx [file pdig.0000598.s008.docx]

Review Paper: Short-term vital parameter forecasting in the intensive care unit - A benchmark study leveraging data from patients after cardiothoracic surgery

**5 vital parameter forecasting in the ICU, it therefore remains to be established whether an increase in model**

**complexity yields better forecasting performance.**

The authors do not seem to use the optimal forecasting models: See: <https://nixtlaverse.nixtla.io/neuralforecast/index.html>

**For compatibility of the two data sources, we down-sampled vital parameters in the internal single-center cohort to five-minute intervals using the median of the spanned values.**

This has the effect of making the vital parameters extremely smooth: A rapid change is particularly interesting for a parameter such as oxygen saturation, which is not recorded in this way.

Have values from the future been used for this averaging? Was the interval [0.5] or [2.5, 7.5] used for the averaging? This could potentially provide the algorithm with information from the future.

**Thus, each ICU admission corresponds to one unique patient and vice versa.**

Have you made sure to keep readmissions in only one data set? A patient who is in the data set several times should not appear in the test and the training set. Otherwise, the accuracy is fictitiously increased.

**Using pre-defined bounds of plausibility for each vital parameter (Table 1), we removed values exceeding**

**these limits and considered them missing.**

Was this done before or after smoothing? The boundaries seem very narrow. Especially with vital parameters, you should not delete outliers, as these sometimes have a particularly high value. What about patients who have died in the ICU? Their vital signs should be below the lower limits.

**Forecasts were always based on the entire observed past up to the current time point. Thus, the length of the forecast window remained fixed at 120 minutes, but the amount of past values on which predictions were based depended on the time point of prediction**

How was this achieved? It is not trivial that a model can use different input lengths. Was it shortened or was the input padded? Does the model work from the first minute or only from a certain point in time?

**Figure 1: Schematic illustration of the forecasting setup. Synthetic example of three successive forecasts produced by the same method for one vital parameter of a single patient. Layout of the figure inspired by [11].**

The model appears to output the average value of the vital parameter but does not react to the individual change of the patient.

**In contrast, for the multivariate neural networks, we trained a single, immutable model on all patients and vital parameters observed in the training set**

Have you tried to train the neuronal networks for just one vital sign?

**Per patient and vital parameter, we produced forecasts every five minutes starting with the sixth, and ending**

**with the penultimate timepoint. Thus, univariate models were given at least five past observations to train on,**

**and all forecasts could be evaluated on at least one true value.**

Does the model need 25 (5x5) minutes of history to work?

**If at least 24 past observations were available for a given vital parameter, we used Box-Cox**

**transformation[22] on the time series with a transformation parameter between zero and two, using the**

**implementation in the forecast package[21].**

What was done before 24 observations? If the other input parameters are scaled, the first 24 must also be scaled.

How was the output scaled back?

MAE, MSME are dependent on the scaling of the data set, therefore this scaling is of great importance, otherwise it is difficult to compare the deviations with other publications. Which I think is an important point of this publication.

Vital parameters were standardized to exhibit mean zero and standard deviation one prior to

**model fitting, and de-standardized post prediction.**

Which data set was used to create the standard scaler? Technically, only the training set should be used for this and the other sets should also be scaled with the scalers from the training set.

**The models were designed to output forecasts for all 24 forecast horizons (5 through 120 minutes) simultaneously.**

How was the variable length of the input time horizon considered?

**We used the HyperOpt search algorithm[26] with trials scheduled and ended prematurely in case of poor performance after at least 10 epochs using Hyperband scheduling[27]. Models were trained for up to 100 epochs with training data presented in batches of 128 patients. 50 different hyperparameter combinations were tried to find the most suitable model**

Which methods did the authors use to minimise overfitting?

**Neural network model parameters For the GRU model, hyperparameter tuning resulted in a single-layer model with hidden size 256, a dropout of 0.37, L2 regularization parameter of 6 · 10-6, which was fitted using a learning rate of 1.31 · 10-3. The optimal Transformer had 3 layers, model dimension 128, hidden size 256, 16 transformer heads, used fixed positional encoding, the rectified linear unit as activation function, a dropout of 0.04, L2 regularization**

**parameter 4.45 · 10-5, and was fitted using a learning rate of 1.25 · 10-3.**

This Information could be transferred to the supplement section.

**Out of the 144 combinations of vital parameter and forecast horizon, the GRU exhibits the smallest RMSE 136**

**imes (94.44%) in the internal test set, and 119 times (82.64%) in the external test set.**

I think an overall MAP and a figure showing this information would be preferable.

**Figure 2: An example of forecasts produced by four different models at a single timepoint for a single patient for all six**

**vital parameters. Times are shifted for additional de-identification of the patient. The ground truth is displayed in black.**

The model does not seem to be able to predict the detailed change in vital signs, but only the trend value. What then is the advantage over a model that simply predicts the value for 2 hours in the future and then interpolates this value?

**Figure 3: Root mean squared error (RMSE) per vital parameter for selected forecast horizons in the internal test set.**

**Different forecasting models are indicated by color. The best performing model (in terms of RMSE) is indicated by *.**

Error bars would be advantageous. What does ‘selected forecast horizons’ mean? Why wasn't the average of all patients in the test set taken?

**Figure 5: Relative improvement on the root mean squared error (RMSE) of the naïve model per vital parameter for**

**selected forecast horizons in the internal test set. Different forecasting models are indicated by color. The best performing model (in terms of RMSE) is indicated by *.**

Personally, I don't find the information in this graphic essential.

**For example, for most patients lower end target values for mean blood pressure and oxygen saturation are 65 mmHg[28] and 92%[29], respectively. By training multivariate neural networks on close to 20,000 ICU admissions, our models were given ample chance to detect, and at prediction time reflect these patterns.**

It would be very interesting to see if the model can predict lower end target values for mean blood pressure and oxygen saturation.

**Given the recent success of Transformer architectures in tasks requiring the processing of sequential data[17,**

**31], our findings regarding the superiority of the GRU to the Transformer in ICU forecasting is somewhat**

**surprising. While the Transformer is competitive and performs better than univariate statistical models in ICU**

Why have you not tested any other Transformer models?

**Our work exhibits four key limitations, which should be addressed in future work. First, our forecasts were**

**based solely on the vital parameters in question.**

Have you tried using more input parameters? This should be stated more clearly in the methodology.

**While obtaining confidence intervals from statistical models like ARIMA and exponential smoothing is straightforward and implemented in frameworks like R’s forecast package[21], it is a much more complex endeavor for recurrent neural networks or Transformers, and requires the implementation of Bayesian modeling, quantile regression or repeated sampling techniques[32].**

It is more complex, but very doable. You can also calculate a confidence interval from the number of errors.

**General**

The paper addresses important questions and is also methodologically sound, as the results are also successfully validated externally. It is also beneficial that the code has been published online.

In general, the quality of English could be improved. Some sentences are not easy to understand, and some technical terms could be replaced by more easily understandable words for the general medical audience.

Most important issue: All models do not really seem to react to the particular details of the vital parameters and only approximate the statistical average value of the respective vital parameter based on the current value. Only the vital sign data was used to predict the vital signs, thus the model cannot account for any medication, patient characteristics or treatment influences, which all have a huge effect on the vital signs. This is probably also the reason why the model is not able to show more complex courses. Also the preprocessing contains a few inaccuracies (see above).
