# Supplementary material for: Short-term vital parameter forecasting in the intensive care unit: A benchmark study leveraging data from patients after cardiothoracic surgery
Source: PLOS Digit Health. 2024 Sep 12;3(9):e0000598. doi: 10.1371/journal.pdig.0000598 (PMC11392423; doi:10.1371/journal.pdig.0000598)
Supplement: Attachment — Submitted filename: response_to_reviewers.docx [file pdig.0000598.s009.docx]

Prof. Dr. med. Alexander Meyer

Department of Cardiothoracic and Vascular Surgery

Deutsches Herzzentrum der Charité

Augustenburger Platz 1

13353 Berlin, Germany

E-Mail: alexander.meyer@dhzc-charite.de

Telephone: +49 30 4593 1000

6 May 2024

Dear Dr. Barage,

we sincerely thank you for your interest in our paper on vital parameter forecasting in the intensive care unit and the opportunity to further improve it through the peer review process.

Please find attached a letter to each of the two reviewers, followed by detailed answers to each of their comments. Changes to the manuscript are listed directly below the response to each comment.

The only changes to the manuscript not directly linked to a reviewer comment are an additional statement in the acknowledgements section regarding research funding of one of our co-authors, and cosmetic changes to plots depicting the root mean squared forecast error, where we changed the color scheme and no longer display the ten-minute forecast horizon due to the addition of two new forecast models.

Please do not hesitate to contact us in case of any further questions or missing information from our side.

Yours sincerely,

Nils Hinrichs	and	Alexander Meyer

**Response to Reviewer 1**

Dear Sir or Madam,

we sincerely thank you for your detailed and well-thought-out review of our manuscript on short-term vital parameter forecasting in the intensive care unit.

Your comments have led to valuable additions and changes to our manuscript. Most notable are the addition of a new class of forecasting models (univariate neural networks), wider boundaries for vital parameters during data cleaning, a new way of summarizing model performances in a single metric through the mean absolute percentage error, and numerous clarifications added to the Materials and Methods section that will greatly benefit the audience. Your comments sparked fruitful discussions within our research group which go beyond this current manuscript.

Please find below our detailed responses to each of your points. Changes to the manuscript are also described for each point in turn.

If you require further clarifications or changes, please do not hesitate to ask.

Thank you again, and best wishes from Berlin.

Yours sincerely

Nils Hinrichs and Alexander Meyer

**Reviewer comment 1:** “… vital parameter forecasting in the ICU, it therefore remains to be established whether an increase in model complexity yields better forecasting performance.” The authors do not seem to use the optimal forecasting models: See: https://nixtlaverse.nixtla.io/neuralforecast/index.html
**Answer:** We thank you for bringing this state-of-the-art forecasting ecosystem to our attention! In combination with your comment 10 (training neural network models for one vital parameter at a time), this discovery led us to add to the families of models applied in the paper.

From the set of models offered by NeuralForecast, we identified N-BEATS and N-HiTS as very recent and very potent univariate forecasting models. Both have shown excellent performances on forecasting benchmarks.

We identified these two models as the most suitable by implementing seven models from NeuralForecast’s collection, training them on a randomly selected subset of 5,000 patients from the training set, and testing their performance on 500 randomly selected patients both from the internal and the external test sets. In both cases, N-BEATS and N-HiTS showed the best performance across vital parameters and forecast horizons (see graphs below). We summarized the performance using MAPE, as you suggested in comment 18.

NeuralForecast offers automatic hyperparameter optimization for their models. We used this functionality, and to guarantee a fair comparison between the models chose the default hyperparameter search space. All models were trained for the same amount of epochs (100) and with the same amount of potential hyperparameter choices (25).

Previously, we distinguished between two classes of models: univariate statistical models, and multivariate neural network models. As you correctly identified in comment 10, univariate neural networks were not part of our study. With the addition of N-BEATS and N-Hits, we now close this gap.

We found that these models perform better than univariate statistical models, but not quite as good as multivariate neural networks, a finding in line with our hypothesis at the onset of this project that increased model complexity could lead to better predictive performance.

**Changes to the manuscript:**

- 9 instead of 7 models (added N-BEATS, N-HiTS)
- 3 instead of 2 model classes (added univariate neural network models)
- 5 instead of 6 forecast horizons displayed in Figures 5 and 6 (dropped 10-minute horizon, otherwise plot was too crowded due to addition of two models)
- New color-scheme to better distinguish between 9 (instead of 7) models
- Exchanged Transformer for N-HiTS in Figure 2 to have a model from all model classes represented
- New table in the Materials and Methods section: Overview of forecasting models

**Reviewer comment 2:** “For compatibility of the two data sources, we down-sampled vital parameters in the internal single-center cohort to five-minute intervals using the median of the spanned values.”
This has the effect of making the vital parameters extremely smooth: A rapid change is particularly interesting for a parameter such as oxygen saturation, which is not recorded in this way.

**Answer:** We agree with your view that down-sampling has a smoothing effect, and we lose potentially interesting information about the vital parameter dynamics. However, this was done with the goal in mind to be able to externally validate our results on the eICU database. The vital parameters in the eICU database unfortunately are only available at the 5-minute frequency and were also down-sampled using the median.

We are of the opinion that the benefit of being able to externally validate our findings and thereby test their robustness outweighs the downside of the loss of information through down-sampling.

**Changes to the manuscript:** None

**Reviewer comment 3:** Have values from the future been used for this averaging? Was the interval [0.5] or [2.5, 7.5] used for the averaging? This could potentially provide the algorithm with information from the future.

**Answer:** We did not use future information for down-sampling. We kept the first observation as is and defined the next value as the 50th percentile of the following five observations. The following table shows a synthetic example of a time-series prior to down-sampling.

| Time | Value |
| --- | --- |
| 00:00:00 | x_1_ |
| 00:01:00 | x_2_ |
| 00:02:00 | x_3_ |
| 00:03:00 | x_4_ |
| 00:04:00 | x_5_ |
| 00:05:00 | x_6_ |
| 00:06:00 | x_7_ |
| 00:07:00 | x_8_ |
| 00:08:00 | x_9_ |
| 00:09:00 | x_10_ |
| 00:10:00 | x_11_ |

We keep the first timepoint and value x_1_ untouched. The next timepoint is 00:05:00, with the value Median(x_2_, x_3_, x_4_, x_5_, x_6_). The value at 00:05:00 therefore only depends on observations up to that point. The same holds true for the next value at 00:10:00, which is the median of x_7_, …, x_11_.

We added to the Materials and Methods section to highlight this important point.

**Changes to the manuscript:**

- Additional sentence in the Materials and Methods section, subsection Study populations

**Reviewer comment 4:** “Thus, each ICU admission corresponds to one unique patient and vice versa.”
Have you made sure to keep readmissions in only one data set? A patient who is in the data set several times should not appear in the test and the training set. Otherwise, the accuracy is fictitiously increased.

**Answer:** If a patient was readmitted to the ICU, we only included the first admission in our analysis. Therefore, a patient can only appear in the training, validation, or test set. We added a sentence to make this clear in the methods section.

**Changes to the manuscript:**

- Additional sentence in the Materials and Methods section, subsection Study populations

**Reviewer comment 5:** “Using pre-defined bounds of plausibility for each vital parameter (Table 1), we removed values exceeding these limits and considered them missing.” Was this done before or after smoothing?

**Answer:** This was done before resampling the internal dataset to the 5-minute frequency. Thus, values exceeding the limits were not included in the resampling. We thank you for raising this important point which was not properly explained in the methods section. We have now added a sentence to clarify this point.

**Changes to the manuscript:**

- Additional sentence in the Materials and Methods section, subsection Data cleaning

**Reviewer comment 6:** The boundaries seem very narrow. Especially with vital parameters, you should not delete outliers, as these sometimes have a particularly high value.

**Answer:** As per your suggestion, we widened the pre-defined limits to minimize the risk of losing values carrying significant information. The new limits are defined as follows:

|  | **Lower limit** | **Upper limit** |
| --- | --- | --- |
| **Systolic blood pressure (mmHg)** | 8 | 350 |
| **Diastolic blood pressure (mmHg)** | 8 | 150 |
| **Mean blood pressure (mmHg)** | 8 | 170 |
| **Central venous pressure (mmHg)** | -15 | 50 |
| **Heart rate (1/min)** | 10 | 330 |
| **Oxygen saturation (%)** | 40 | 100 |

We implemented these limits mostly because the sensors are prone to producing artefacts. For example, ECG lead displacement through patient movement leads to a recorded heart rate of zero and occurs frequently in our dataset. A further example are upward spikes in blood pressure measurements that occur when samples for blood gas analysis are taken, which repeatedly lead to mean systemic blood pressure values of 180 mmHg and beyond.

We kept 8 as the lower limit for blood pressure values, as this corresponds to the remaining hydrostatic pressure observed even without blood flow.

We increased the upper end of blood pressure measurements as per your suggestion.

Central venous pressure can now take values below zero, which can occur in cases of severe dehydration or patients sitting or standing in an upright position. For values below -15, we believe it is more likely to be an artefact than a valid measurement, and therefore exclude these values.

The new lower limit of oxygen saturation is 40, which corresponds to the value observed in desaturated venous blood.

**Changes to the manuscript:**

- Changes to Table 1 (Materials and Methods, subsection Data cleaning)
- All calculations were re-run with the new limits.

**Reviewer comment 7:** What about patients who have died in the ICU? Their vital signs should be below the lower limits.

**Answer:** For systolic, diastolic, and mean blood pressure, as well as oxygen saturation, the new limits reflect values that would occur in deceased patients (see comment 6). We had to keep 10 as the lower bound for the heart rate due to the artefacts arising from misplaced sensors which frequently yield a value of zero.

Most importantly though, it is not uncommon to stop monitor surveillance during end-of-life care to reduce noise, anxiety, and stress. Vital signs of patients, where therapy limitation was initiated, are therefore oftentimes not reflected in the datasets.

**Changes to the manuscript:** See Reviewer comment 6.

**Reviewer comment 8:** “Forecasts were always based on the entire observed past up to the current time point. Thus, the length of the forecast window remained fixed at 120 minutes, but the amount of past values on which predictions were based depended on the time point of prediction” How was this achieved? It is not trivial that a model can use different input lengths. Was it shortened or was the input padded? Does the model work from the first minute or only from a certain point in time?

**Answer:** We need to answer these questions separately for the different model classes deployed in the paper. In response to comments 1 and 10, we added a new class of models to the paper: univariate neural network models.

Univariate statistical models

We do not make forecasts with univariate models from the first timepoint. At the first five timepoints, no forecasts are made. Theoretically, the naïve model could make a prediction using only a single observation, but all other univariate models need a minimum number of observations to produce forecasts. The way we implemented it, we have an initial forecast for the timepoint 30 minutes after ICU admission. If a patient was admitted at 12:00, the first forecast is made at 12:25 and covers the timeframe from 12:30 to 14:25.

Since we re-trained all univariate models from scratch at every timestep, we were able to easily handle the different input lengths by simply appending the latest observation to the training data and re-training the model.

Multivariate neural network models

We used padding in cases where the ICU stay was shorter than 24 hours. In these cases, we added padding to the end of the time-series to have input vectors of fixed length. However, the forecasts produced at these timepoints were simply excluded, as they did not correspond to any observed values.

The GRU and the Transformer used in this study can generate predictions at every timestep of a time-series. We made sure that there is no leakage of future information. For the GRU, this was achieved by using a uni-directional architecture (contrary to a bi-directional architecture, which reads a time-series both backwards and forwards). For the Transformer, this was done by explicitly masking future values in the computation of the attention mechanism.

The multivariate models work from the first minute. However, to achieve even terms in the comparison of univariate and multivariate models, we discarded forecasts produced in the first five timesteps, since they could not be compared to any univariate forecasts.

Univariate neural network models

Univariate neural network models, implemented using NeuralForecast, are also capable of producing predictions from the first timepoint, but same as with multivariate models, we discarded forecasts produced in the first five timesteps.

During model training, NeuralForecast automatically handles the issue of varying input through padding. However, forecasts from padded elements are discarded.

At prediction time, we manually pass the fitted model more and more observations, and extract the forecast at each timepoint.

**Changes to the manuscript:** None

**Reviewer comment 9:** “Figure 1: Schematic illustration of the forecasting setup. Synthetic example of three successive forecasts produced by the same method for one vital parameter of a single patient. Layout of the figure inspired by [11].” The model appears to output the average value of the vital parameter but does not react to the individual change of the patient.

**Answer:** Figure 1 does not show real patient data. It depicts a synthetic example for illustrative purposes. The goal of the figure is to show how forecast errors for different horizons (minutes ahead) were computed, and how from timepoint to timepoint the observed past increases, whereas the forecast window remains fixed at 120 minutes. To illustrate the forecast errors, it was advantageous to use a synthetic example with considerable and visible error. We added further clarification to the figure caption.

**Changes to the manuscript:**

- Change to caption of Figure 1, Materials and Methods, subsection Forecast methodology

**Reviewer comment 10: “**In contrast, for the multivariate neural networks, we trained a single, immutable model on all patients and vital parameters observed in the training set” Have you tried to train the neuronal networks for just one vital sign?

**Answer:** As mentioned in response to comment 1, we added a new class of models to our analysis: univariate neural network models. As part of this class, we implemented N-BEATS and N-HiTS using the NeuralForecast software ecosystem.

**Changes to the manuscript:** See comment 1

**Reviewer comment 11: “**Per patient and vital parameter, we produced forecasts every five minutes starting, and ending with the penultimate timepoint. Thus, univariate models were given at least five past observations to train on, and all forecasts could be evaluated on at least one true value.” Does the model need 25 (5x5) minutes of history to work?

**Answer:** A detailed answer is given above in response to your comment 8, which raised the question of varying input lengths. In short: Univariate and multivariate neural network models work from the first observation. Univariate statistical models need at least some history to train on because they only have access to one time-series at a time. We therefore only produced forecasts from univariate models with at least five observations to train on.

**Changes to the manuscript:** None

**Reviewer comment 12:** “If at least 24 past observations were available for a given vital parameter, we used Box-Cox transformation[22] on the time series with a transformation parameter between zero and two, using the implementation in the forecast package[21].” What was done before 24 observations? If the other input parameters are scaled, the first 24 must also be scaled. How was the output scaled back?

**Answer:** Box-Cox transformations were only used in univariate models, which we re-trained from scratch at every timepoint using the entire time-series up to that point. (The multivariate models are flexible enough to not need this pre-processing step.)

If at a timepoint there were less than 24 prior observations available, we did not apply Box-Cox and left the time-series untouched on the original scale. The resulting forecasts were therefore also unscaled.

At a later timepoint where the time-series has at least length 24, the entire time-series including the first 24 entries was Box-Cox-transformed. The resulting forecasts then need to be re-transformed to the original axis. This could be achieved by storing the Box-Cox transformation parameter needed for re-transformation.

In summary, we either transformed the entire time-series used to train the univariate models or left it untouched. Within a time-series, we did not mix transformed and untransformed elements.

**Changes to the manuscript:**

- Additional sentence in the Materials and Methods section, subsection Univariate statistical models

**Reviewer comment 13:** MAE, MSME are dependent on the scaling of the data set, therefore this scaling is of great importance, otherwise it is difficult to compare the deviations with other publications. Which I think is an important point of this publication.

**Answer:** You are absolutely correct, the scaling of the data is crucial and comparisons are only viable if the forecasts are all on the same scale. We had already stated for uni- and multivariate neural network models that predictions were de-standardized, but we also added a corresponding sentence to clarify that univariate statistical model predictions were inverse-transformed to retain the original scale.

**Changes to the manuscript:** See Reviewer comment 12

**Reviewer comment 14:** “Vital parameters were standardized to exhibit mean zero and standard deviation one prior to model fitting, and de-standardized post prediction.” Which data set was used to create the standard scaler? Technically, only the training set should be used for this and the other sets should also be scaled with the scalers from the training set.

**Answer:** As you laid out, we used only the training set to identify the scaling parameters. This information was missing in the methods section, and we added it accordingly.

**Changes to the manuscript:**

- Additional sentences, Materials and Methods section, subsections Uni- and multivariate neural network models

**Reviewer comment 15:** “The models were designed to output forecasts for all 24 forecast horizons (5 through 120 minutes) simultaneously.” How was the variable length of the input time horizon considered?

**Answer:** For the most part, this has already been answered in response to comment 8. In short, univariate statistical models were re-trained with every new observation. Uni- and multivariate neural network models used padding to account for ICU stays shorter than 24 hours, but forecasts from padded elements were discarded.

**Changes to the manuscript:** None

**Reviewer comment 16:** “We used the HyperOpt search algorithm[26] with trials scheduled and ended prematurely in case of poor performance after at least 10 epochs using Hyperband scheduling[27]. Models were trained for up to 100 epochs with training data presented in batches of 128 patients. 50 different hyperparameter combinations were tried to find the most suitable model” Which methods did the authors use to minimise overfitting?

**Answer:** The answer to this question depends on the model, so we try to answer it separately for our three model classes.

Univariate statistical models

The naïve model, the Theta method and exponential smoothing have too little flexibility for overfitting to be a relevant issue.

We minimized overfitting of the ARIMA model by restricting the number of autoregressive of moving average model components to at most three.

The AR NNet model was restrained from overfitting by limiting the autoregressive order to 6 and including a weight decay term which shrinks the model parameters.

Multivariate neural network models

The most important precaution used to avoid overfitting was to evaluate hyperparameter choices (and eventually choose the best model specification) on the validation set. The validation had no overlap with the training set. Therefore, models which overfitted the training set performed poorly on the validation set, whereas models that fit the training set nicely without overfitting performed well on the validation set.

Apart from the model validation procedure, certain model architecture choices were also put in place to avoid overfitting. We used weight decay (L2 regularization) to shrink model parameters. By adding dropout layers, we also limited the model capacity to overfit the training data. Further, the decision to decide on a maximum number of epochs before model training can also be seen as a counter-step against overfitting, as this makes it impossible for models to indefinitely improve their performance on the training set.

Univariate neural network models

As described for multivariate neural network models, evaluating hyperparameter choices and model performances on validation data which is separate from the test data is the most important precaution against overfitting. We also limited the number of epochs during model training, and chose not to use any neural network layers with more than 128 hidden units. The N-HiTS model also allows dropout layers, which we applied.

**Changes to the manuscript**: None

**Reviewer comment 17:** “Neural network model parameters For the GRU model, hyperparameter tuning resulted in a single-layer model with hidden size 256, a dropout of 0.37, L2 regularization parameter of 6 · 10-6, which was fitted using a learning rate of 1.31 · 10-3. The optimal Transformer had 3 layers, model dimension 128, hidden size 256, 16 transformer heads, used fixed positional encoding, the rectified linear unit as activation function, a dropout of 0.04, L2 regularization parameter 4.45 · 10-5, and was fitted using a learning rate of 1.25 · 10-3.” This Information could be transferred to the supplement section.

**Answer:** As suggested, we transferred this section to the appendix, where we transformed it into a table containing vital parameters per model.

**Changes to the manuscript:**

- Removed subsection Neural network model parameters
- Added tables containing hyperparameter choices to the appendix

**Reviewer comment 18:** “Out of the 144 combinations of vital parameter and forecast horizon, the GRU exhibits the smallest RMSE 136 times (94.44%) in the internal test set, and 119 times (82.64%) in the external test set.” I think an overall MAP and a figure showing this information would be preferable.

**Answer:** This is an excellent suggestion, which we gladly applied. For the internal and external test sets, we added a figure each displaying the mean absolute prediction error across all vital parameters and forecast horizons. In accordance with comment 20, we added error bars to these plots as well.

**Changes to the manuscript:**

- New Figures 3 and 4, summarizing model performance using MAPE
- Dropped section cited in the reviewer comment counting how often models exhibit the best RMSE

**Reviewer comment 19:** “Figure 2: An example of forecasts produced by four different models at a single timepoint for a single patient for all six vital parameters. Times are shifted for additional de-identification of the patient. The ground truth is displayed in black.” The model does not seem to be able to predict the detailed change in vital signs, but only the trend value. What then is the advantage over a model that simply predicts the value for 2 hours in the future and then interpolates this value?

**Answer:** Predicting two hours into the future and interpolating is an interesting suggestion. However, it raises the question of how to optimally interpolate. Linear interpolation would be an option but is quite inflexible. It would also not allow for forecasts in the immediate future (e.g. 5 minutes ahead) to be *below* the current value at forecast time, and forecasts further ahead (e.g. 2 hours ahead) to be *above* the current value.

We can see from the plot that the models do not linearly interpolate. ARIMA, for example, tends to move quickly towards the level of the 2-hours-ahead forecast in the beginning, and then more slowly for forecasts approaching the 2-hour mark. This can be seen as exponential interpolation to the 2-hour forecast, with the interpolation speed depending on the trained ARIMA parameters.

N-HiTS and GRU also do not linearly interpolate, and – as can be seen from the N-HiTS forecast of central venous pressure in the new Figure 2 – have the capacity to produce flexible up and down movement in the forecasts.

An important thing to keep in mind is that at the time the forecast is produced, future interventions like medication applied within the next two hours are unknown but can greatly impact the development of the vital parameters. Producing rather smooth forecasts should therefore be seen as the model’s way of accounting for possible future developments, which in the ICU setting can encompass quite drastic and fast-approaching changes both in up- and downward direction.

**Changes to the manuscript:** None

**Reviewer comment 20:** “Figure 3: Root mean squared error (RMSE) per vital parameter for selected forecast horizons in the internal test set. Different forecasting models are indicated by color. The best performing model (in terms of RMSE) is indicated by *.” Error bars would be advantageous.

**Answer:** As suggested, we added error bars representing 95% confidence intervals. The intervals were estimated using bootstrapping.

**Changes to the manuscript:**

- Error bars added to all figures summarizing forecast errors
- Description of bootstrapping procedure added in Materials and Methods section, subsection Statistical analysis

**Reviewer comment 21:** What does ‘selected forecast horizons’ mean? Why wasn't the average of all patients in the test set taken?

**Answer:** ‘Selected forecast horizons’ refers to the fact that only the 5-, 15-, 30-, 60-, and 120-minute forecasts are displayed, although we produced forecasts for every five-minute interval up to 120. (In the previous version, the 10-minute forecast was also displayed, but as mentioned in response to comment 1, we dropped it after the addition of two new models.)

The values displayed were indeed calculated from all patients in the test set.

**Changes to the manuscript:** None

**Reviewer comment 22:** “Figure 5: Relative improvement on the root mean squared error (RMSE) of the naïve model per vital parameter for selected forecast horizons in the internal test set. Different forecasting models are indicated by color. The best performing model (in terms of RMSE) is indicated by *.” Personally, I don't find the information in this graphic essential.

**Answer:** The figures have been removed.

**Changes to the manuscript:**

- Removed Figures 5 and 6

**Reviewer comment 23:** “For example, for most patients lower end target values for mean blood pressure and oxygen saturation are 65 mmHg[28] and 92%[29], respectively. By training multivariate neural networks on close to 20,000 ICU admissions, our models were given ample chance to detect, and at prediction time reflect these patterns.” It would be very interesting to see if the model can predict lower end target values for mean blood pressure and oxygen saturation.

**Answer:** While we share the interest in this question, we feel that it is outside of the scope of this benchmark study. Predicting specific events like breaching a lower boundary is a classification problem, which needs to be analyzed using different tools than a forecasting problem. As we mentioned in the introduction of our paper, predicting specific events in the ICU is a well-established field of machine learning research, whereas pure forecasting research is currently underdeveloped. Predicting specific events via forecasting is an intriguing proposition, but would require extensive work to compare the results against event predictions from other methods like classification trees or neural networks specifically trained on the classification task at hand. We believe this is a topic for future research based on this benchmark paper.

**Changes to the manuscript:** None

**Reviewer comment 24:** “Given the recent success of Transformer architectures in tasks requiring the processing of sequential data[17, 31], our findings regarding the superiority of the GRU to the Transformer in ICU forecasting is somewhat surprising. While the Transformer is competitive and performs better than univariate statistical models in ICU” Why have you not tested any other Transformer models?

**Answer:** Our initial thought process when we implemented the transformer model was as follows. There are endless options for state-of-the-art forecasting models, but we are limited in the number of models we can implement and present to the reader. As we were aiming for a benchmarking paper, we decided to limit ourselves to one recurrent neural network and one transformer architecture, knowing that this does not cover the entire collection of any of these two classes of models, and neither does it cover potential combinations of the two approaches. Apart from comparing individual forecast models, we see a strength of our paper in the overall comparison of classes of forecasting models, of which we now compare three (thanks to your suggestions in comments 1 and 10). At the onset of the project, it was not clear whether state-of-the-art multivariate neural networks such as the Transformer or the GRU could even out-perform simple univariate statistical models. We therefore initially decided to implement only a one Transformer architecture.

Forecasting via transformers is also a very new field and much less mature than forecasting via statistical models or recurrent neural networks. There is still an ongoing debate whether the transformer architecture is even suitable for time series forecasting (for example, see Zeng et al. 2022: “Are Transformers Effective for Time Series Forecasting?”). Multiple Transformer-based forecasting architectures have however been proposed in the literature in recent years.

We used our experiment described in response to comment 1 to decide on the optimal Transformer architecture to implement as a multivariate model as a reference for the architecture by Zerveas et al. currently implemented in the paper. We compared a Vanilla Transformer, Informer, Autoformer and PatchTST and chose PatchTST based on the results presented below comment 1.

We implemented PatchTST based on the official github repository of the PatchTST paper (<https://github.com/yuqinie98/PatchTST>). We tuned the same hyperparameters as described for the previously implemented Transformer with the same search space. Additionally, we tuned the patch length (8, 16, 24 or 32) and the stride length (8, 16, 24 or 32), two parameters specific to the PatchTST architecture. The number of epochs (100), hyperparameter choices (50) and hyperparameter optimization routine (HyperBand scheduling) were as described in the paper.

The results are shown below (left for the external test set, right for the internal test set). Although clearly better than the Naïve model, its performance is not as good as that of the GRU or our previously implemented Transformer architecture.

For lack of evidence to suggest otherwise, we therefore decided to stick with our previous implementation. Further research will have to answer the question whether PatchTST or other architectures like Informer or Autoformer can somehow be unlocked to provide better forecasting results than the GRU.

**Changes to the manuscript:** None

**Reviewer comment 25:** “Our work exhibits four key limitations, which should be addressed in future work. First, our forecasts were based solely on the vital parameters in question.” Have you tried using more input parameters? This should be stated more clearly in the methodology.

**Answer:** We now state more clearly in the methods section that other data sources were not part of this study. We discuss this in quite some detail in the discussion section, where we also discuss that this does not alter the message of our benchmark paper.

Initially, we tried to estimate multivariate models with access to more input variables, but faced the following problems that led us to decide to keep to vital parameters as our only data source for this study.

We assume that the applied medication has the strongest immediate impact on the vital parameters among potentially available parameters. In our institution, medication is tracked in the electronic patient record, but only upon manual input by the medical staff. There is no guarantee that changes are uploaded immediately, and we know from experience that this can take several minutes and typos occur frequently.

In the multi-center eICU cohort, this problem is sure to be present as well. On top, medication is not tracked in a standardized way in the eICU database and in many cases, the exact amount of the active ingredient could not be found , because only the volume of the infusion solution but not the dosage was tracked..

Laboratory values are tracked in a more standardized way in the eICU database, but in our internal cohort, we have no way of determining when blood samples were taken, since only the timestamp of upload of the results into the electronic health record is stored.

Due to these complications, and due to our goal of establishing benchmarks to be improved upon in future work, we refrained from tackling these issues at this point. We hope to pick this up again in future work.

**Changes to the manuscript:**

- Additional sentence in the Materials and Methods section, subsection study populations

**Reviewer comment 26:** “While obtaining confidence intervals from statistical models like ARIMA and exponential smoothing is straightforward and implemented in frameworks like R’s forecast package[21], it is a much more complex endeavor for recurrent neural networks or Transformers, and requires the implementation of Bayesian modeling, quantile regression or repeated sampling techniques[32].” It is more complex, but very doable. You can also calculate a confidence interval from the number of errors.

**Answer:** We agree in principle that it is doable and are interested in tackling this issue. However, we feel this question deserves its own research paper. There are multiple ways of calculating confidence intervals, e.g. by modeling the entire distribution instead of the conditional mean, or as you suggested by estimating intervals from prediction errors. All methods come with different assumptions, advantages, and disadvantages. A paper diving into this topic would in our opinion need to address these points and perform a thorough evaluation of the resulting intervals which is beyond the scope of our benchmark paper for point forecast performance.

**Changes to the manuscript:** None

**General remarks by the reviewer:** The paper addresses important questions and is also methodologically sound, as the results are also successfully validated externally. It is also beneficial that the code has been published online.
**General remark 1:** In general, the quality of English could be improved. Some sentences are not easy to understand, and some technical terms could be replaced by more easily understandable words for the general medical audience.

**Answer:** We apologize for causing readers difficulties. It is our goal to present our findings in an easily understandable language. We went over the manuscript and made the following changes:

- Introduction: *plethora* is now *multitude*
- Introduction: *well-tuned* is now *well-designed*
- Materials and Methods: *hyperparameter tuning* is now *hyperparameter optimization*
- Materials and Methods: *post-processing* is now *manual adjustment*
- Materials and Methods: *Hyperparameter specifications* are now *hyperparameter choices*
- Materials and Methods: Hyperparameter trials are now *set up* instead of *scheduled, and Hyperband scheduling* is now the *Hyperband algorithm*
- Discussion: *deep learning* is now *neural networks*
- Discussion: *cross-learning* is now *simultaneously learning from many independent, yet related examples*
- Discussion: *content-agnostically fitted one time-series at a time* is now *fitted independently for each patient*

If you would be so kind and point out further technical terms that should be replaced and sentences that should be restructured in a more digestible way, we would be more than happy to do so.

**Changes to the manuscript:** See answer above.

**General remark 2:** Most important issue: All models do not really seem to react to the particular details of the vital parameters and only approximate the statistical average value of the respective vital parameter based on the current value. Only the vital sign data was used to predict the vital signs, thus the model cannot account for any medication, patient characteristics or treatment influences, which all have a huge effect on the vital signs. This is probably also the reason why the model is not able to show more complex courses.

**Answer:** We assume this refers to the four model outputs at a single point in time for one patient depicted in Figure 2. Clearly, all four depicted models in Figure 2 show much smoother behavior than the ground truth. The most important thing to note in this regard is that all forecasts depicted were produced at timepoint 06:00 based only on the information available up to this timepoint. While especially blood pressure (systolic, diastolic, and mean) values show significant up and down movements after 06:00, the following points need to be accounted for in the evaluation of the forecasts:

1) The patients in the dataset are undergoing continuous monitoring and medical treatment to stabilize their condition. At the time of prediction, the exact timing and extent of future therapy cannot be known as it might not be decided yet or depend on external factors like availability or experience of physicians.
2) Since the vital signs can display substantial variance, a model which tries to capture the full variability might occasionally produce very good forecasts, but would on other occasions deviate strongly from the true behavior, for example if it miscalculated the length of an upward or downward cycle and was subsequently misaligned with the ground truth. Models are therefore incentivized not to display highly varying patterns in their produced forecasts, but rather capture the most likely trend.

As depicted in Figures 3 - 6, on average, there are major differences between forecasting methodologies, which give a clear indication of which methodologies to advance and improve in future research.

**Changes to the manuscript:** None

**General remark 3:** Also the preprocessing contains a few inaccuracies (see above).

**Answer:** Please find our answers to questions regarding preprocessing below the relevant questions above.

**Changes to the manuscript:** See reviewer comments 2 – 6 and 12 – 14.

**Response to Reviewer 2**

Dear Sir or Madam,

we sincerely thank you for your detailed and well-thought-out review of our manuscript on short-term vital parameter forecasting in the intensive care unit.

Your comments have led to valuable additions and changes to our manuscript. Most notable are an addition to the limitation section discussing the operationalization of forecasts in the ICU, a more detailed description of the patient cohorts presented in this study, and a new approach in our handling of missing data. Your comments sparked fruitful discussions within our research group which go beyond this current manuscript.

Please find below our detailed responses to each of your points. Changes to the manuscript are also described for each point in turn.

If you require further clarifications or changes, please do not hesitate to ask.

Thank you again, and best wishes from Berlin.

Yours sincerely

Nils Hinrichs and Alexander Meyer

**General remarks by the reviewer:** Thank you for submitting this interesting research.
The study focuses on short-term forecasting of vital parameters in ICU patients post-cardiothoracic surgery. It compares various univariate statistical models and multivariate neural networks for predicting vital signs. The research finds multivariate neural networks, particularly GRU and Transformer models, outperform univariate models especially for longer forecast horizons. The study establishes comprehensive benchmarks for ICU vital parameter forecasting, suggesting the feasibility and utility of AI-driven models in clinical decision-making in ICU settings.
However, I believe this study contains the following issues to be considered by authors:

**Reviewer comment 1:** The major practical concern is that the predicted parameters are not easily utilized in real clinical setting - which actually should be the biggest limitation of all (to be in the limitation section) for this work. The evaluation of the prediction performance of vital signs by 4 different models suffer from the lack of visualization. For example, the Figure 2 shows a very long horizon of prediction of each model at the time of prediction - and each color flies out only based on the fixed time.

**Answer:** The use-case of accurately predicting vital parameters could potentially be of benefit to clinicians, in particular for anticipating pathological trajectories, i.e. in response to an intervention. However, the goal of this study was to establish a robust benchmark as a first step to accelerate research in this domain.

According to your suggestion, we added to the limitations section a fifth and final point regarding forecast visualization and operationalization in ICUs. In it, we specifically addressed your point regarding the forecast horizon, which we set to two hours in this study, although different lengths might be more suitable in different medical fields or even different stages of a patient’s ICU stay.

From the thousands of forecasts produced in this study, Figure 2 depicts a single example of forecasts produced at a single point in time for one patient for illustrative purposes. Aggregates across points in time and patients are depicted in later figures. We illustrated a subset of four models in Figure 2 because the comparison of forecasting techniques is at the core of our work. However, in clinical settings, only the best-performing model would be estimated displayed to the medical staff. Distinguishing between suitable and less suitable models was a key research question of this paper.

**Changes to the manuscript:**

- New paragraph in the limitations section.

**Reviewer comment 2:** The authors could show the temporal model performance by showing the model to be on the moving windows - hence visualizing the model accuracy (MAPE or MAE + standard deviation) in regression metrics better, as the time goes to the future.

**Answer:** We acknowledge that fact that visualizing temporal dynamics in this use case is of utmost importance. To guide the reader to this topic we 1) illustrated the forecasting setup and definitions of forecast errors of differing horizons at different points in time in Figure 1, and exemplified concrete model outputs in a representative single patient case in Figure 2.

Figure 1 displays how model accuracy is tracked across moving windows. However, metrics such as MAPE or MAE cannot be displayed in plots comparing vital parameters with true values, since they are averages across all patients and timepoints. As depicted in Figure 1, only deviations of forecasts from the ground truth can be displayed.

How the model accuracy goes down when forecasting further into the future is depicted in Figures 3 and 4, which clearly show how forecasting gets more and more difficult with increased forecasting horizon.

**Changes to the manuscript:** None

**Reviewer comment 3:** For readers, any of those 4 models seem to be suboptimally performing.

**Answer:** We assume this refers to the four model outputs at a single point in time for one patient depicted in Figure 2. Clearly, all four depicted models in Figure 2 show much smoother behavior than the ground truth. The most important thing to note in this regard is that all forecasts depicted were produced at timepoint 06:00 based only on the information available up to this timepoint. While especially blood pressure (systolic, diastolic, and mean) values show significant up and down movements after 06:00, the following points need to be accounted for in the evaluation of the forecasts:

3) The patients in the dataset are undergoing continuous monitoring and medical treatment to stabilize their condition. At the time of prediction, the exact timing and extent of future therapy cannot be known as it might not be decided yet or depend on external factors like availability or experience of physicians.
4) Since the vital signs can display substantial variance, a model which tries to capture the full variability might occasionally produce very good forecasts, but would on other occasions deviate strongly from the true behavior, for example if it miscalculated the length of an upward or downward cycle and was subsequently misaligned with the ground truth. Models are therefore incentivized not to display highly varying patterns in their produced forecasts, but rather capture the most likely trend.

As depicted in Figures 3 - 6, on average, there are major differences between forecasting methodologies, which give a clear indication of which methodologies to advance and improve in future research.

**Changes to the manuscript:** None

**Reviewer comment 4:** Authors might want to present quantitative difference between the model and real performances - supplying Bland-Altman plots could help as well.

**Answer:** We quantified the difference between models and the ground truth using the RMSE, and displayed it for a selection of forecast horizons in Figures 5 and 6.

Although we were at first intrigued by the idea of Bland-Altman plots, we chose not to add them for the following reasons:

1) Bland-Altman plots do pairwise comparison between two methods. In this paper, however, we compare more than a handful of methods on six vital parameters for forecast horizons from 5 to 120 minutes, and would therefore require an impractical amount of Bland-Altman plots.
2) Due to the vast amount of observations in our study, each Bland-Altman plots would be uninterpretable due to the six-digit number of points to be displayed.

We feel that Figures 3 - 6 are sufficient to support the goal of this research paper: To provide benchmarks for forecast performance, and to distinguish between suitable and less suitable forecasting approaches.

**Changes to the manuscript:** None

**Reviewer comment 5:** Also it would be beneficial for the paper to include the techniques and approaches used to model these dynamic changes, as well as an analysis of how these temporal variations impact the prediction outcomes.

**Answer:** Each forecasting model used in this study has its own specific way of modeling temporal dynamics, ranging from very simple (e.g. in the naïve model) to mathematically complex (e.g. Transformer). To keep the manuscript length at a digestible level, we refrain from going deep into the statistical details behind the applied models. In the Materials and Methods section, we state which methods we use, how we specifically configured them to our need, and supply the interested reader with appropriate sources on the mathematical details of the methods.

As for the impact of temporal variations on the prediction outcome, we assume that you are referring to specific events in the vital parameter time series of a patient (as opposed to temporal variations as a general feature of all time-series). We did not go into any details on this topic for the following reasons:

1) To make such an evaluation, we would need clear definitions of events in question. There is a multitude of temporal dynamics in vital parameters loosely associated with pathologies. However, exact definitions depend on the medical field and the specific use case. We feel that selecting specific definitions of temporal dynamics and analyzing their impact on forecast performance merits separate research projects.
2) Any such analysis would be specific to our cohort, post-operative cardiothoracic surgery patients. While we leverage this cohort for benchmarking and for a proof-of-concept, we do not want to limit this research paper to this cohort, and feel like this contribution to PLOS Digital Health should be kept as interesting as possible to readers from a multitude of medical fields.

**Changes to the manuscript:** None

**Reviewer comment 6:** Line 98 – 99: Has the study accounted for the potential noise in the data within the first 30 mins to 1 hour after ICU admission? This initial period can often include transitional dynamics that may not be representative of the patients’ stable physiological state.

**Answer:** As described in the Materials and Methods section, forecasts on the first five timepoints per patient are not part of the analysis. However, the data of this transitional period is not discarded, but is part of the input data used for predictions at later timepoints.

To analyze the impact of the initial timeframe after ICU admission, we calculated the mean absolute percentage error across all vital parameters, grouped by full hours since admission. The results are depicted below, first for the internal test set, second for external test set. The analysis is limited to horizons <= 30 minutes, because in the first hour, forecasts with longer horizons do not exist.

As you hypothesized, the first hour after ICU admission is the hardest to forecast. For all models, the performance is at its lowest point in this period. However, there are two possible factors driving this low performance early in the ICU stay: Transitional dynamics, but also the fact that there is simply less data for models to work with. The second point is supported by the fact that the performance gradually improves over the next hours, reaching its peak in the 8 to 12 hours range. Differentiating between these two effects is a complex endeavor.

All models in this study are supplied with the same patient data. While transitional dynamics during the first hour after ICU admission might not be representative of a patient’s eventual stable state, this does not illegitimize comparisons between different models. Contrary, we feel like this further adds to the need to identify suitable models, because patients go through different phases during their ICU stay, all of which need to be properly handled by a well-performing model.

We thank you for bringing up this valid and interesting point regarding the transitional dynamics. In the interest of keeping the manuscript length within reasonable limits and not further add to the already complex forecasting setup, we refrain from adding this sub-analysis to the manuscript and argue that it does not render any of our analyses less relevant of robust.

**Changes to the manuscript:** None

**Reviewer comment 7:** Line 109 – 111: Could the authors provide the percentage or extent of missing data encountered in this study? Understanding the volume of missing values is crucial for assessing the robustness of the imputation techniques and the reliability of the models’ predictions, especially in a complex ICU setting.

**Answer:** We now discuss the degree of missingness in the text and added a table with details to the Appendix. For the internal data, which we down-sampled to match the 5-minute frequency of the eICU database, we reported missingness both for the original and the down-sampled datasets.

As we mentioned in the Statistical analysis section, missing values were not counted towards the computation of the forecast performance. We thereby sought to limit the impact of missing data and imputation on our experiments.

**Changes to the manuscript:**

- Addition to the Results section (Study population)
- Additional table in the Supplemental materials

**Reviewer comment 8:** Further, both of ‘Forward-filling’ or ‘Median imputation’ might not account for individual patient variability and trends, which are critical in the ICU setting. This could affect the models’ ability to accurately predict outcomes. More sophisticated techniques like time-series imputation methods or predictive modeling could potentially provide more accurate ways to handle missing data.

**Answer:** We agree that the methods previously chosen for imputation were kept quite simple. Based on your suggestions, we improved the imputation strategy (see further down). However, for the following reasons, we believe that a robust and computationally simple imputation strategy is adequate for this research paper.

1) As mentioned in the statistical analysis section, missing values are not counted towards the forecast performance. Therefore, the imputation strategy does not affect error computation, but is limited to impacting forecasts through the inputs given to models.
2) Comparison of different forecasting models is a core question of our paper. We impute values before any models are fitted and therefore feed all models with the same data. Therefore, there is a “level playing field” when it comes to comparing the different models. In other words, however well or poorly we impute, all models deal with the same data, so the comparison of their performance is unaffected.
3) For our research question, any imputation strategy can only use the data up to the timepoint with missing information. Otherwise, information from the future (which we are trying to forecast) would leak into the past, resulting in unrealistic forecasting behavior. We are therefore limited to imputation strategies which use only past information.
4) In cases where a previous value is known, we previously used forward-filling, which is equivalent to forecasting with the naïve method. More sophisticated methods (you mentioned time-series imputation or predictive modeling) make use of forecasting models to impute from past information. However, at the onset of the project, we had no notion of which forecasting methods perform well at forecasting vital signs in the ICU, since the literature on the subject is scarce. Time-series imputation or predictive modeling would therefore have required knowledge which are only trying to generate with this paper.

Despite these points, we took your argument very seriously, that the individual patient variability was not adequately captured with our previous imputation strategy. We therefore adjusted it in the following way.

1) We implemented a model-based imputation strategy, which leverages relations between vital parameters (implemented in the scikit-learn software package as iterative imputing). For each vital parameter, regression models are fitted on the training set which model the relationship to all other vital parameters. In case of a missing value at a given timepoint *t*, these relationships are then exploited to insert the most likely value given the non-missing vital parameter values at timepoint *t* for the patient in question.
2) Missing values at the start of a patient’s ICU admission (where no previous value is known) are now implemented via the previously mentioned model-based approach. For example, if the first mean blood pressure measurement is missing but other vital parameters are recorded, the missing value can be imputed from a model that has been trained on the relationship of mean blood pressure to systolic and diastolic blood pressure (as well as central venous pressure, heart rate, and SpO2).
3) In cases where a previous value is known, we previously used unlimited forward-filling. We now limit forward-filling to at most three observations. If more than three subsequent observations are missing, we impute using the model-based approach starting with the fourth observation.

We believe these changes are a suitable compromise which keep the imputation strategy simple, while also improving the handling of individual vital sign dynamics.

While this change surely had an impact on individual forecasts, the change in the approach did not alter the overall message of this research paper.

**Changes to the manuscript:**

- Changes to the imputation strategy
- Changes to the Data cleaning section of the manuscript

**Reviewer comment 9:** Line 200 – 206: In the comparison between the internal cohorts and the external test set, it is observed that different statistical measures are used: for instance, the lower quartile for central venous pressure and the median for heart rates. It would be helpful to know if these differences were statistically significant.

**Answer:** The comparison of the vital parameter distributions in the different cohorts used in this study is an important issue. As you suggested, tests for statistical significance (usually reported via p-values) are the standard method. However, we feel that in our study they are not appropriate, because of the very large number of observations in our data. The sample size is a driving force in most test statistics, with a direct impact of the power of a test. As a result, when the sample size is large enough, even small differences between cohorts are accompanied by a small p-value. We would like to illustrate this using two examples from our study.

Let us assume we want to compare systolic blood pressure between two cohorts using the t-test. We estimated the standard deviation of the variable in the training set (originally for scaling purposes) and found it to be 23.92 mmHg. We consider a difference of 0.5 mmHg or less between the two groups to be of no clinical relevance.

However, if we compute the necessary sample size per group needed to detect this clinically negligible difference of 0.5 mmHg with standard parameters for significance level (5%) and power (80%), the required sample size is roughly 36 000 per group. For reference, there are close to 2 million observations in the eICU cohort, and close to 5 million in the internal test sets. We calculated that we have sufficient observations to reach a 5% significance level and 80% power to detect even a difference of only 0.07 mmHg between groups.

As a further example, to detect a difference in heart rate between two cohorts of 0.1 beats per minute, roughly 400 000 observations per group would suffice (using our estimate for the standard deviation of the heart rate of 16.25 beats per minute).

Consequently, adding p-values to Table 2, yields “<0.001” as the only entry. We therefore do not consider p-values to be an informative source for assessing differences between our cohorts.

However, we agree that our description of cohorts in Table 2 does not give interested readers a full picture of vital parameter distributions. We therefore graphically depicted the distribution of all six vital parameters used in this study across cohorts using violin plots in a Supplemental Figure, which we referenced in the text.

**Changes to the manuscript:**

- Added Supplemental Figure 1: Distribution plots of vital parameters across study cohorts.
- Referenced Supplemental Figure 1 in the Results section.
