# Supplementary material for: Short-term vital parameter forecasting in the intensive care unit: A benchmark study leveraging data from patients after cardiothoracic surgery
Source: PLOS Digit Health. 2024 Sep 12;3(9):e0000598. doi: 10.1371/journal.pdig.0000598 (PMC11392423; doi:10.1371/journal.pdig.0000598)
Supplement: Attachment — Submitted filename: response_to_reviewers_2.docx [file pdig.0000598.s010.docx]

Prof. Dr. med. Alexander Meyer

Department of Cardiothoracic and Vascular Surgery

Deutsches Herzzentrum der Charité

Augustenburger Platz 1

13353 Berlin, Germany

E-Mail: alexander.meyer@dhzc-charite.de

Telephone: +49 30 4593 1000

16 July 2024

Dear Dr. Barage,

We highly appreciate the opportunity to further improve our manuscript on vital parameter forecasting in the intensive care unit.

As requested, we reviewed the reference list. We found none of the cited papers to be retracted. Upon review, we updated the following citations: For reference 7, we added the full name of the conference where the paper was originally published. For references 9, 12 and 17, we removed “Epub ahead of print” upon checking that the papers were indeed published in the respective journal. Two new references (37 and 38) were added as part of the changes made in response to the reviewer comment on prediction intervals.

As this was also brought up by the reviewer, we checked the figure resolution, but, unless we are mistaken, found it to be in line with the journal requirements. Please let us know if changes are required and we will gladly comply as fast as possible.

Finally, in response to the reviewer comment on prediction intervals, we extended the relevant paragraph in the limitations section and wrote an in-depth answer to the reviewer, arguing that prediction intervals merit their own separate research due the multitude of potential approaches and the difficulty of measuring their success due to the inherent tradeoff of interval width and coverage rate. If you or the reviewer have any further questions or suggestions for improvement, we would be glad to hear them.

Yours sincerely,

Nils Hinrichs	and	Alexander Meyer

**Response to reviewer 1**

Dear Sir or Madam,

We greatly appreciate your verdict that the manuscript has improved. We share this sentiment and feel that this is in large parts due to your constructive and detailed feedback for which we would again like to sincerely thank you.

Regarding your comment on prediction intervals, we agree with you that quantifying the uncertainty associated with forecasts is of great importance and should be part of the product if or when vital parameter forecasts were to be made available to medical professionals in the ICU.

In your initial response, you laid out a potential way towards estimating prediction intervals by estimating them from observed errors. Indeed, a prediction interval for the *h*-steps-ahead forecast can be obtained via the formula $\left[ \hat{y_{T+h}}\pm1.96* \hat{\sigma_{h}} \right]$, where $\hat{y_{T+h}}$ refers to the forecast *h* steps into the future and $\hat{\sigma_{h}}$ to the estimated standard deviation which can potentially be derived from observed errors. There are two strong underlying assumptions behind this interval: a) that the residuals follow a normal distribution and b) that the variance of the residuals is constant for all past and future observations of the time-series (referred to as *homoscedasticity*). Under these assumptions, for statistical models like ARIMA or exponential smoothing, formulas for $\hat{\sigma}_{h}$ have been derived which take into account the forecast horizon *h*, the variance of the underlying time-series process, and the uncertainty associated with the estimation of the parameters of the model.[1]

If assumptions a) and b) are true, prediction intervals of the form described above offer a coverage rate of 95%. It would therefore not be of interest to compare prediction intervals of different models regarding their fixed coverage rate. Intervals would merely differ in their width, which depends on the residual variance. However, residual variance and mean squared error are closely related, and models with the lowest RMSE (as computed as part of our study) would also exhibit the narrowest prediction intervals. Under these conditions, we therefore argue that there would be no additional value in comparing prediction intervals of our models, as they all exhibit the same coverage rate, and the width of the intervals is directly related to the already studied point forecast quality.

The matter becomes much more interesting if assumptions a) and/or b) were not true. If only a) was not true, we could estimate empirical quantiles of our residuals and add them to the point forecasts to obtain intervals. However, since the 2.5^th^ or 97.5^th^ percentile cannot be estimated from a small number of residuals, this approach would only work after a run-in period much larger than the one we used for point forecast comparison, if we want patient-specific intervals. Assuming homoscedasticity, this approach should also yield coverage rates of approximately 95% for all models, and we would need to compare widths of intervals. Again, we argue that models with small forecast errors in terms of RMSE also yield the narrowest empirical error distributions and therefore narrowest prediction intervals from this approach.

However, in our specific context of vital parameters measured in an ICU following cardiothoracic surgery, we see no arguments for neither assumption a) nor b). Transitional effects immediately post-surgery, sudden derangements of the health status and effects of administered medication make the ICU such an interesting case study for forecasting, but go against the aforementioned assumptions. The interesting question in this context is therefore whether models can produce intervals of adaptive size, depending on the patient- and time-specific level of uncertainty. As we were able to show, modern neural network techniques show the best ability to produce point forecasts in this context, which we attribute to their flexibility and their ability to perform cross-patient learning, instead of treating each patient individually without further context. It would appear logical that the same models that produce the best point forecasts are also in a good position to produce good intervals. However, for prediction intervals in the context of deep learning on time-series data, we do not see any evidence that straight estimation of uncertainty from observed errors is the optimal solution. Recent publications on the matter do not even mention this possibility,[2, 3] presumably because of the strong assumptions underlying this approach. Although we agree that this simple approach would be a logical benchmark and could be included in a work studying the performance of prediction intervals, the majority of the academic focus seems to be on quantile regression, Bayesian methods, resampling techniques or conformal predictions (a model-agnostic way of deriving intervals from an independent calibration set, which in its original description relied on uncorrelated errors, but has recently been extended to the time-series domain). [2–4]

Apart from the methodologies behind the computation of prediction intervals, they are also more difficult to compare than point forecast performance. Less accurate models could potentially compensate for their shortcomings by producing wider intervals. The comparison of multiple approaches to interval forecasting therefore needs to account both for interval width and coverage at the same time. We feel that this would divert attention from the main message of our benchmark paper, which is the superiority of machine learning models over statistical models in our specific use case. Given this superiority, one could even argue that a follow-up paper on prediction intervals should discard poorly performing models, and focus on models with better point forecast quality, for which multiple ways of producing intervals could be compared in all necessary details.

Your remark has given us the opportunity to dive even deeper into the topic of prediction intervals. As a result, we altered the relevant paragraph of the limitations section. First, we fixed a misleading wording and now only speak of prediction intervals (instead of previously mixing in the term confidence interval). Second, we added that for statistical models, the derivation of intervals implemented in R’s forecast package relies on strong assumptions, as mentioned above. Third, we added conformal predictions as their own separate line of research on prediction intervals, since they have very recently gotten a lot of attention.[2, 4, 5] Finally, we added a sentence on the difficulty of comparing intervals due to the tradeoff between width and coverage.

As was the case for all your comments, we feel it has challenged us in a positive way and thus led to major improvement of our paper. We would be delighted to hear whether you feel that we were able to appropriately state our arguments and adjust the relevant section. If you feel like the section should be removed altogether to ensure readers are not distracted from the main message of the benchmark paper, or if you have an altogether different suggestion, we would be delighted to hear it.

Yours sincerely

Nils Hinrichs and Alexander Meyer

[1]	Hyndman RJ, Athanasopoulos G. *Forecasting : principles and practice, 3rd edition*. Heathmont, Vic.: OTexts: Melbourne, Australia, 2021.

[2]	Stankeviciute K, Alaa AM, van der Schaar M. Conformal Time-Series Forecasting. *35th Conference on Neural Information Processing Systems (NeurIPS 2021)*.

[3]	Alaa A, Schaar M. Frequentist Uncertainty in Recurrent Neural Networks via Blockwise Influence Functions. *International Conference on Machine Learning 2020*

[4]	Auer A, Gauch M, Klotz D, et al. Conformal prediction for time series with modern hopfield networks. *Adv Neural Inf Process Syst*; 36 (2024).

[5]	Xu C, Xie Y. Sequential predictive conformal inference for time series. *International Conference on Machine Learning 2022*
